# Structural and biochemical impact of C8-aryl-guanine adducts within the *Nar*I recognition DNA sequence: influence of aryl ring size on targeted and semi-targeted mutagenicity

**DOI:** 10.1093/nar/gku1093

**Published:** 2014-10-31

**Authors:** Michael Sproviero, Anne M.R. Verwey, Katherine M. Rankin, Aaron A. Witham, Dmitriy V. Soldatov, Richard A. Manderville, Mostafa I. Fekry, Shana J. Sturla, Purshotam Sharma, Stacey D. Wetmore

**Affiliations:** 1 Department of Chemistry and Toxicology, University of Guelph, Guelph, Ontario, Canada, N1G 2W1; 2 Department of Health Sciences and Technology, Institute of Food, Nutrition and Health, ETH Zürich, 8032 Zürich, Switzerland; 3 Pharmacognosy Department, Faculty of Pharmacy, Cairo University, Kasr El-Aini, Cairo 11562, Egypt; 4 Department of Chemistry & Biochemistry, University of Lethbridge, Lethbridge, AB, Canada, T1K 3M4

## Abstract

Chemical mutagens with an aromatic ring system may be enzymatically transformed to afford aryl radical species that preferentially react at the C8-site of 2′-deoxyguanosine (dG). The resulting carbon-linked C8-aryl-dG adduct possesses altered biophysical and genetic coding properties compared to the precursor nucleoside. Described herein are structural and *in vitro* mutagenicity studies of a series of fluorescent C8-aryl-dG analogues that differ in aryl ring size and are representative of authentic DNA adducts. These structural mimics have been inserted into a hotspot sequence for frameshift mutations, namely, the reiterated G_3_-position of the *Nar*I sequence within 12mer (*Nar*I(12)) and 22mer (*Nar*I(22)) oligonucleotides. In the *Nar*I(12) duplexes, the C8-aryl-dG adducts display a preference for adopting an *anti*-conformation opposite C, despite the strong *syn* preference of the free nucleoside. Using the *Nar*I(22) sequence as a template for DNA synthesis *in vitro*, mutagenicity of the C8-aryl-dG adducts was assayed with representative high-fidelity replicative versus lesion bypass Y-family DNA polymerases, namely, *Escherichia coli* pol I Klenow fragment exo^−^ (Kf^−^) and *Sulfolobus solfataricus* P2 DNA polymerase IV (Dpo4). Our experiments provide a basis for a model involving a two-base slippage and subsequent realignment process to relate the miscoding properties of C-linked C8-aryl-dG adducts with their chemical structures.

## INTRODUCTION

**Figure 1.**
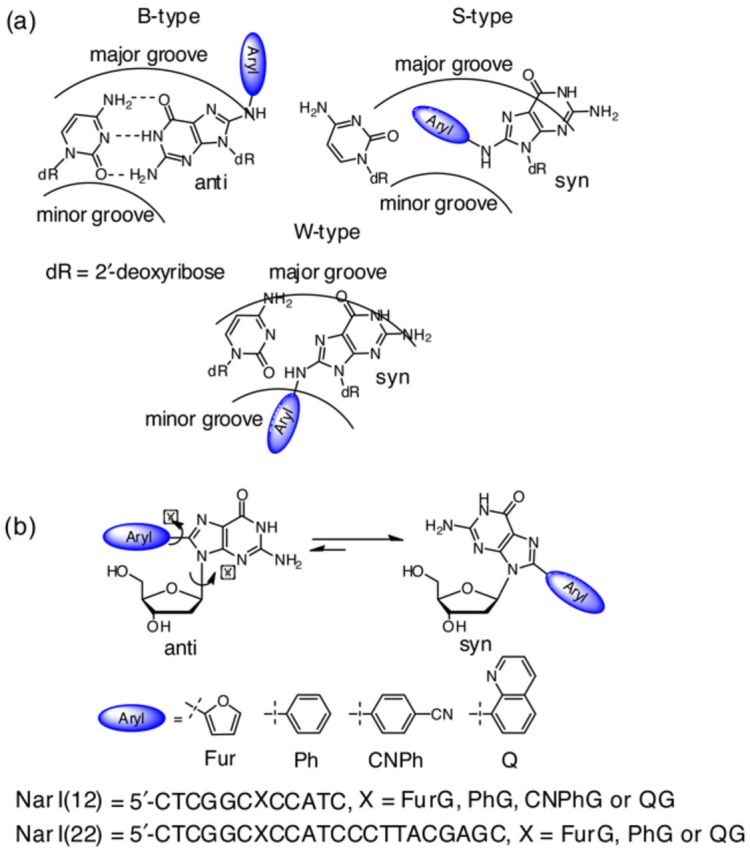
(a) Depictions of the three major conformations produced by N-linked C8-dG adducts. (b) Structures of C-linked C8-aryl-dG adducts and oligonucleotide sequences of *Nar*I(12) and *Nar*I(22). The dihedral angle χ (∠(O4′C1′N9C4)) defines the glycosidic bond orientation to be *anti* when *χ* = 180 ± 90º or *syn* when *χ* = 0 ± 90º; *θ* (∠(N9C8C10C11) for PhG, CNPhG and QG and ∠(N9C8C10O11) for FurG) defines the degree of twist between the nucleobase and the C8-aryl group.

The genetic integrity of a living cell is constantly assaulted by various chemical species reacting with DNA. A common means of DNA damage is the formation of bulky DNA adducts (addition products), which can initiate cancer when occurring at either protooncogene or tumor suppressor gene segments of the genome (1,2). Arylamines and heterocyclic amines (3) are known human carcinogens that undergo metabolic activation to produce bulky DNA adducts with C8-substituted 2′-deoxyguanosine (C8-dG) adducts being the major lesions detected *in vivo* (4). The resulting N-linked C8-dG adducts contain a flexible amine tether separating the dG component from the aryl ring and there is a strong established relationship between the resulting conformational heterogeneity and biological effects (5,6). The structurally related aromatic organic carcinogens, such as polycyclic aromatic hydrocarbons (PAHs) (7–9), arylhydrazines (10–12) and phenols (13–16), may also undergo bioactivation, producing aryl radical species that covalently attach to the C8-site of dG to produce carbon-linked C8-aryl-dG adducts. In these cases, the adducts are similar in structure to their N-linked counterparts in terms of aryl ring size and shape, but do not have an amine linkage, thereby altering the orientation of the modification in the DNA duplex as well as reducing conformational flexibility. Furthermore, in contrast to N-linked C8-dG adducts, there is limited understanding of the relationship between the structures of C-linked C8-dG adducts and their miscoding potential.N-Linked C8-dG adducts can adopt three distinct conformations depending on the orientation about the glycosidic bond (*anti* or *syn*) and the location of the bulky aryl ring within the duplex (Figure 1a) (5,6). In the B-type conformation, the adduct maintains Watson–Crick (W-C) hydrogen (H)-bonding with the opposing cytosine (C), which directs the C8-aryl moiety into the solvent-exposed major groove (17,18). In the base-displaced stacked (S-type) conformation, the base adopts the *syn* conformation and stacks the aryl ring between the neighbouring base pairs, which ruptures W-C H-bonding with the opposing C to displace it out of the helix (19–22). In the wedge (W-type) conformation (23,24), the C8-dG adduct also adopts the *syn* conformation, but steric clash at the lesion site, or Hoogsteen H-bonding with the opposing base, places the aryl moiety in the minor groove. The interplay between aryl ring size (18), adduct planarity (17) and DNA sequence (25,26) dictate conformational distribution, which are important factors that affect repair propensity (27,28) and mutational outcomes (29–33).

Carbon-linked C8-aryl-dG adducts demonstrate a relatively strong preference for the *syn* conformation (defined by the angle *χ*, Figure 1b) (34). In addition, C-linked adducts are non-planar and exhibit a high degree of twist between the nucleobase and C8-aryl group (defined by the angle *θ*, Figure 1) in order to reduce steric interactions that arise due to decreased flexibility (34). Similar to N-linked adducts, the formation of C-linked C8-dG adducts has been implicated in a wide variety of mutagenic outcomes. For example, the PAH benzo[a]pyrene (B[a]P) undergoes peroxidase-mediated oxidation to afford radical cations (7,8), where the potential involvement of the C8-B[a]P-dG lesion produces G → T and G → C transversion mutations in yeast (9). Additionally, arylhydrazines generate phenyl radicals, produce C8-phenyl-dG adducts (10–12) and are mutagenic in bacteria (35). Finally, the chlorophenolic mycotoxin ochratoxin A (OTA) gives rise to a C-linked C8-aryl-dG adduct *in vivo* (14,15) and deletion mutations have been observed in the kidney tissue of male rats exposed to OTA (36–38).

The progress of DNA synthesis by high-fidelity (replicative) DNA polymerases—*Escherichia coli* DNA polymerase I Klenow fragment exo^−^ (Kf^−^), and mammalian DNA pol α is strongly blocked by C8-phenyl-dG (PhG) (39). High-fidelity polymerases can fit one templating nucleotide in their active site, favouring accurate replication when correct W-C base pairing is established with the incoming dNTP (40). Due to this induced-fit mechanism of replication, bulky adducts in the template strand often stall or block the progression of replication by high-fidelity polymerases through distortion of the DNA duplex and polymerase active site. Such stalling *in vivo* is believed to be a signal for the recruitment of Y-family translesion polymerases for potential error-free extension past the bulky adduct after polymerase switching (40–43). Translesion polymerases have a solvent-exposed active site that is spacious enough to accommodate bulky DNA lesions, while facilitating low-fidelity DNA replication. Thus, potential mutagenic bypass of PhG that may occur *in vivo* is anticipated to rely on translesion polymerases.

The DNA polymerase IV from *Sulfolobus solfataricus* P2 (Dpo4) is a prototypical Y-family polymerase and is regarded as an excellent model for investigating how structural features of adducts determine lesion bypass efficiency and fidelity (40–43). Dpo4-catalyzed bypass of modified oligonucleotides containing N-linked C8-dG adducts may proceed in an error-free or error-prone manner (31,33). In non-iterated sequences, the C8-dG adduct produced by 2-amino-3-methylimidazo[4,5-*f*]quinoline (IQ) undergoes error-free bypass by Dpo4 (31), while extension past the C8-dG adduct produced by 2-aminofluorene (AF) leads to both full-length product and mis-elongated products containing targeted (at the lesion site, i.e. base substitution) and semitargeted (in the vicinity of the lesion site, i.e deletion) mutations (33). Extension past the C8-IQ-dG adduct with the lesion present in a reiterated (CG-dinucleotide repeat) sequence has also been assessed using Dpo4 and found to result in two-base deletion mutations (31). Thus, Kf^−^ and Dpo4 were employed in the present work to investigate how C-linked C8-aryl-dG adducts with different aryl rings impact replication by high-fidelity and translesion polymerases. The results were expected to demonstrate the mutagenic potential of such lesions, establish the impact of aryl ring size and permit comparison to their N-linked C8-dG counterparts.C-Linked C8-aryl-dG adducts are difficult to incorporate into oligonucleotides in a site-specific manner because they are sensitive to acid-catalyzed deglycosylation (44), thereby limiting the adaptation of standard solid-phase synthesis with 5′-*O*-DMT protection for this purpose (45). To overcome this limitation, we developed an efficient solid-phase protocol for inserting representative C8-aryl-dG adducts into the reiterated G_3_-position (X) of *Nar*I Type II restriction endonuclease recognition sequence (5′-CTCG_1_G_2_CXCCATC) (46). The *Nar*I sequence was used for *in vitro* mutagenicity studies of arylamines (31), and the reiterated G_3_-position (X) is a hotspot for frameshift mutations induced by N-linked C8-dG adducts (47) via a two-base slippage mechanism (48).

The C8-aryl-dG adducts (viz. C8-aryl = furyl (FurG), phenyl (PhG), 4-cyanophenyl (CNPhG) and quinolyl (QG), Figure 1b) chosen for this study differ in their ring size (5-membered ring in FurG versus 6-membered ring in PhG and CNPhG versus fused ring in QG), type of substituent (PhG versus CNPhG) and ring type (carbocyclic rings in PhG and CNPhG versus heterocyclic rings in FurG and QG). This series of adducts was designed on the basis of their structural similarities to natural carcinogen DNA-adducts, but having interesting fluorescence properties that we could exploit to define their conformations in DNA (49–51) and establish the relationship between adduct structure and mutagenicity *in vitro*. Among these adducts, PhG is a known adduct derived from the carcinogen phenylhydrazine (*vide supra*). CNPhG is a mimic for adducts arising from *para*-substituted aryl hydrazines, such as *para*-CH_3_-PhG, *para*-HOOC-PhG and *para*-H_3_COC-PhG (10–12). QG is a mimic for a portion of PAH lesions (e.g. C8-B[a]P-dG) (7,8) where additional phenyl rings are 2,3-fused to the C8-phenyl group, which extends the C8-substituent in a bent-fashion. In the present study, we performed optical experiments (ultraviolet (UV) thermal melting, circular dichroism (CD) and fluorescence) combined with molecular dynamics (MD) simulations to define the structural features of all four adducts within the *Nar*I(12) duplexes. Primer elongation experiments were then carried out using the *Nar*I(22) template containing the three adducts with different ring types (FurG, PhG and QG) to assess adduct size and shape on DNA replication *in vitro* by the polymerases Kf^−^ and Dpo4. The results of these studies demonstrate for the first time the relationships between C8-aryl ring size, adduct conformation and mutagenic outcome for C-linked C8-aryl-dG adducts.

## MATERIALS AND METHODS

### Materials

All adducted *Nar*I(12) and *Nar*I(22) oligonucleotide substrates were prepared on a 1 μmol scale using a BioAutomation MerMade 12 automatic DNA synthesizer using standard or modified β-cyanoethylphosphoramidite chemistry. Full synthetic details of modified phosphoramidites and electrospray ionization mass spectrometry (ESI-MS) analysis of modified *Nar*I oligonucleotides have been previously published (46). Unmodified oligonucleotides (*Nar*I(12), *Nar*I(22), complementary strands for *Nar*I(12) and 15mer primer) were purchased from Sigma Genosys and were purified by Sigma using polyacrylamide gel electrophoresis. *E. coli* pol I Klenow fragment exo^−^ (Kf^−^) and T4 polynucleotide kinase were purchased from New England BioLabs, while *Sulfolobus solfataricus* P2 DNA polymerase IV (Dpo4) was purchased from Trevigen Inc. Isotopically labelled adenosine triphosphate (ATP) ([γ-^32^P]-ATP) was purchased from PerkinElmer.

### X-ray diffraction study of QG

The free nucleoside was prepared by Suzuki–Miyaura cross-coupling of 8-Br-dG and quinoline-8-boronic acid in 2:1 H_2_O:CH_3_CN. The reaction mixture was diluted with 20 ml H_2_O and the pH adjusted to 6–7 with 1 M HCl (aq). The mixture was allowed to cool to 0°C for several hours before pale yellow crystals were recovered by vacuum filtration. A thick plate from the bulk crystalline product was studied at 150 K on a SuperNova Agilent single-crystal X-ray diffractometer with a microfocus Cu*K*_α_ (*λ* = 1.54184 Å) radiation source and Atlas Charged Coupled Device (CCD) detector. Diffraction intensity data were collected using ω-scan to the max 2θ of 148.7°. The unit cell parameters were refined using the entire data set. The structure was solved by direct methods and refined to *R* = 0.025 and GOF = 1.105. Details of the crystal structure refinement and full crystallographic information are given in the Supplementary Data and have been deposited with the Cambridge Crystallographic Data Centre (deposition no. 10135599). The formula of the crystal studied is QG*H_2_O (C_19_H_20_N_6_O_5_), FW = 412.41 (394.39+18.02); the structure is orthorhombic, space group *P*2_1_2_1_2_1_, unit cell dimensions (150 K): *a* = 6.68426(5), *b* = 9.28108(8), *c* = 29.6696(2) Å, *V* = 1840.62(2) Å^3^; *Z* = 4.

### UV thermal melting

All melting temperatures (*T*_m_) of *Nar*I(12) oligonucleotides were measured using a Cary 300-Bio UV-Vis spectrophotometer equipped with a 6 × 6 multicell Peltier block-heating unit using Hellma 114-QS 10 mm light path cells. Oligonucleotide samples were prepared in 50 mM phosphate buffer, pH 7, with 100 mM NaCl, using equivalent amounts (6.0 μM) of the unmodified or C8-aryl-dG modified *Nar*I(12) oligonucleotide and its complementary strand. The UV absorption at 260 nm was monitored as a function of temperature and consisted of forward-reverse scans from 10°C to 90°C at a heating rate of 1°C/min, which was repeated five times. The *T*_m_ values of the duplexes were determined using hyperchromicity calculations provided in the Varian Thermal software.

### CD measurements

Spectra were recorded on a Jasco J-815 CD spectropolarimeter equipped with a 1 × 6 multicell block thermal controller and a water circulator unit. Measurements were carried out in 50 mM phosphate buffer, pH 7, with 100 mM NaCl, using equivalent amounts (6.0 μM) of the unmodified or C8-aryl-G modified *Nar*I(12) oligonucleotide and its complementary strand. Quartz glass cells (110-QS) with a light path of 1 mm were used for measurements. Spectra were collected at 10°C between 200 and 400 nm, with a bandwidth of 1 nm and scanning speed at 100 nm/min. The acquired spectra were the averages of five accumulations that were smoothed using the Jasco software.

### Fluorescence studies of *Nar*I(12) oligonucleotides

All fluorescence spectra were recorded on a Cary Eclipse Fluorescence Spectrophotometer equipped with a 1 × 4 multicell block Peltier stirrer and temperature controller. *Nar*I(12) samples were prepared in 50 mM phosphate buffer, pH 7, with 100 mM NaCl. In each case, both excitation and emission spectra were recorded for the C8-aryl-G modified *Nar*I(12) oligonucleotide hybridized to its complementary strand at 10°C. All single-strand oligonucleotide samples were prepared to a final concentration at 6.0 μM, and duplex samples were prepared using equivalent amounts (6.0 μM) of the C8-aryl-G modified *Nar*I oligonucleotide and its complementary strand. All measurements were made using quartz cells (108.002F-QS) with a light path of 10 × 2 mm, and excitation and emission slit-widths were kept constant at 5 nm. All fluorescence excitation spectra were recorded at the emission wavelength (maximum) of the C8-aryl-G adduct, from 200 to 10 nm below the emission wavelength, while fluorescence emission spectra were recorded at the excitation wavelength (maximum) of the adduct, from 10 nm above the excitation wavelength to 600 nm.

### Computational details

MD simulations were performed on the *Nar*I(12) (5′−CTCG_1_G_2_CG_3_CCATC) oligonucleotide containing the adducted nucleoside (FurG, PhG, CNPhG or QG) at the G_3_-position. Initial structures were built using both *syn* and *anti* conformations of the adducted nucleosides. For FurG and QG, the *syn* and *anti* conformations were built using two different orientations of the asymmetric C8-ring system with respect to the nucleobase, which are related by an approximate 180° flip along the *θ* torsion angle. The starting DNA structure was built using the NAB module of AMBER 11 (52), and the C8-modification was introduced at the G_3_-position using Gaussview (53). The RED.v.III.4 (54) program was used to calculate the partial charges of the adducted nucleosides, while parameters for bonded terms were taken from the generalized AMBER force field (55) according to atom types assigned using ANTECHAMBER 1.4 (56). The parmbsc0 (57) modification to the parmm99 (58) force field was used for the natural nucleosides. Each DNA system was neutralized with 22 sodium ions and solvated with an 8 Å T1P3P (59) truncated octahedral water box. Initial minimization was performed on the water molecules and sodium ions with the DNA fixed, using a steepest descent algorithm for the first 500 steps, and a conjugate gradient algorithm for the next 500 steps. Subsequently, the entire energy of the system was minimized for 2500 steps using 1000 steps of steepest descent followed by 1500 steps of conjugate gradient minimization. The system was then heated from 0 to 300 K using Langevin temperature equilibration during which the DNA was weakly restrained with a force constant of 10 kcal mol^−1^ Å^−2^ to avoid large fluctuations within the DNA, and simulated for 20 ps at constant volume. Subsequently, a 40 ns constant pressure MD simulation was carried out on each system using the PMEMD module of AMBER 11 (52) or 12 (60). A representative structure was chosen for analysis from each simulation by clustering the entire corresponding simulation with respect to the *θ* and *χ* dihedral angles of the adducts using the ptraj module of AMBER. Free energies were calculated from each simulation trajectories within the MM-PBSA approximation (61). In these calculations, the molecular mechanics and solvation-free energy terms were evaluated using the Poisson–Boltzmann approach and 1000 snapshots (1 snapshot every 20 ps from the simulation trajectory), while the entropy term was evaluated from normal mode calculations using 100 snapshots (1 snapshot every 200 ps).

### Radiolabelling and annealing

T4 polynucleotide kinase and [γ-^32^P]ATP were used to label the 15mer primer strands at the 5′-end. The unmodified and modified DNA primer/template duplexes were prepared by annealing the 15mer primer and *Nar*I(22) template strands (50% excess of template strand) through heating the mixtures to 95°C for 10 min, followed by slow cooling to room temperature overnight.

### Single-nucleotide incorporation assays

Kf^−^ or Dpo4 were used to perform primer extension reactions on each previously labelled and annealed primer/template duplex in the presence of dCTP, dGTP, dATP or dTTP. Reactions were initiated by the addition of the dNTP (at a final concentration of 25, 50, 75 or 100 μM) to enzyme/DNA mixtures to give a final reaction volume of 10 μl. The final concentrations for Kf^−^ assays were 50 mM NaCl, 10 mM Tris-HCl (pH 7.9), 10 mM MgCl_2_, 1 mM dithiothreitol (DTT), 100 nM duplex and 1 nM Kf^−^ for the unmodified duplex, and 10 nM Kf^−^ for each of the modified duplexes. The final concentrations for Dpo4 assays were 50 mM NaCl, 50 mM Tris (pH 8.0), 2.5 mM MgCl_2_, 5 mM DTT, 100 μg/ml bovine serum albumin, 5% glycerol, 100 nM duplex and 10 nM Dpo4. Reactions were incubated at 37°C for 1 h with Kf^−^, and 30 min with Dpo4, followed by 4 μl transferred and mixed with 36 μl of loading dye (95% formamide, 20 mM ethylenediaminetetraacetic acid, 0.05% xylene cyanol and bromophenol blue) to terminate the reaction. Note that 4 μl of these quenched reactions were then subjected to 15% polyacrylamide gel electrophoresis in the presence of 7 M urea and incorporation products were visualized from a phosphorimaging screen using a Bio-Rad phosphorimager. Band quantification was performed using Quantity One® software. Density analysis was performed by circumscribing each band of interest, followed by the software generating density values for each. Using Excel, the relative intensity of each band was determined as a percentage by dividing the density of a single band by the total density of all bands in the specific lane and multiplying by 100.

### Full-length extension assays

Kf^−^ or Dpo4 were used to perform primer extension reactions on each previously labelled and annealed primer/template duplex in the presence of a 100 μM 4dNTP mix (25 μM each dNTP). Increasing concentrations of each enzyme was used for a final concentration of 5, 10, 20 nM of Kf^−^ or Dpo4, except a final concentration of 0.5, 1, 2 nM of Kf^−^ was used with the unmodified duplex. All other final concentrations in each reaction were the same as outlined for the single nucleotide incorporation assays. Reactions were initiated by the addition of the dNTP mix to enzyme/DNA mixtures to give a final reaction volume of 10 μl and were incubated at 37°C for 1 h with Kf^−^, or 30 min with Dpo4. Following incubation, reactions were quenched and incorporation products were resolved in the same manner as above.

## RESULTS

### *Nar*I(12) properties

#### UV thermal melting

Phosphoramidite chemistry was used to incorporate the C-linked C8-aryl-dG adducts into the G_3_-position of *Nar*I(12) (Figure 1b) (46). UV-derived thermal melting parameters for the modified *Nar*I(12) oligonucleotides are summarized in Table 1. The unmodified duplex had a *T*_m_ = 63.6°C under our experimental conditions. The C8-aryl-dG adducts paired opposite C resulted in universal destabilization of all duplexes (Δ*T*_m_ = −9.1°C to −18.6°C), which increased with increasing C8-aryl ring size, i.e. FurG < CNPhG ≈ PhG << QG. The benzo[a]pyrene radical cation-derived adducts (9) and PhG (39) are known to promote G insertion in the complementary strand, thereby introducing a G:G mismatch. When the modified *Nar*I(12) oligonucleotides were paired opposite strands containing an opposing G in order to analyse the degree of G:G mismatch stabilization, the C8-aryl-dG adducts had variable influences on duplex stability. Compared to the unmodified G:G mismatched duplex, the stability of duplexes with G paired opposite FurG (+3.6°C), CNPhG (+1.5°C) and PhG (+0.7°C) were stabilized, while with QG (−9°C) the mismatched duplex was destabilized. To assess the influence of π-stacking interactions in the absence of H-bonding, we paired adducts opposite the stable tetrahydrofuran (THF) model of an abasic site (62). The Δ*T*_m_ values ranged from QG (−5.8°C) < PhG (−3.5°C) < FurG (−3°C) < CNPhG (+1.6°C). The stabilization resulting from the electron-withdrawing cyano group may be rationalized on the basis of increased conjugation and enhanced duplex-stabilizing stacking interactions. In contrast, the extended π-surface of QG decreased duplex stability, but in this case, the large degree of twist between the quinolyl moiety and G may preclude favourable π-stacking interactions within the duplex.

**Table 1.** Thermal melting parameters of C8-aryl-dG modified *Nar*I(12).

5′-CTC-GGC-X-CCA-TC-3′				5′-CTC-GG-CX-CCA-TC-3′			
3′-GAG-CCG-N-GGT-CT-5′				3′-GAG-CC—-GGT-AG-5′(-2)			
*X*	*N*	*T*_m_ (°C)^a^	Δ*T*_m_^b^	*X*	*N*	*T*_m_ (°C)^a^	Δ*T*_m_^b^
G	C	63.6	–	G	THF	45.7	–
FurG	C	54.5	−9.1	FurG	THF	42.7	−3.0
PhG	C	47.8	−15.7	PhG	THF	42.2	−3.5
CNPhG	C	48.9	−14.6	CNPhG	THF	47.3	+1.6
QG	C	45.0	−18.6	QG	THF	39.9	−5.8
G	G	54.0	–	G	−2	39.4	–
FurG	G	57.6	+3.6	FurG	−2	36.8	−2.6
PhG	G	54.7	+0.7	PhG	−2	36.5	−2.9
CNPhG	G	55.5	+1.5	CNPhG	−2	38.8	−0.6
QG	G	45.0	−9.0	QG	−2	39.4	0.0

^a^*T*_m_ values of duplexes (6.0 μM) measured in 50 mM sodium phosphate buffer, pH 7, with 0.1 M NaCl, heating rate of 1ºC/min, errors are ±1ºC.

^b^Δ*T*_m_ = *T*_m_ (modified duplex) – *T*_m_ (unmodified duplex).

Hybridizing the modified *Nar*I(12) sequences with the truncated 10-mer sequence (–2) provides a bulged duplex that models the intermediate state involved in a slippage-mediated mechanism of mutation (48). Enhanced stability of the slipped mutagenic intermediate (SMI) is believed to promote −2 frameshift mutagenesis mediated by N-linked C8-dG adducts in the *Nar*I sequence (63–66). For example, the truncated *Nar*I(12) duplex containing the C8-IQ-dG lesion at G_3_ (*X*) exhibits a Δ*T*_m_ value of +10°C compared to the unmodified control in 0.1 M NaCl (63). Under similar conditions the N-linked C8-dG adduct of N-acetyl-2-aminofluorene (AAF) affords a Δ*T*_m_ value of +15°C (64,65). In the SMI, the N-linked C8-dG adducts adopt a *syn* conformation that promotes intercalation of the extended C8-aryl ring system (66). In contrast, the C-linked C8-aryl-dG lesions fail to stabilize the truncated *Nar*I(12) duplex compared to the unmodified control (Table 1). The *T*_m_ values increase in the order PhG (36.5°C) ≈ FurG (36.8°C) < CNPhG (38.8°C) < QG (39.4°C).

#### Circular dichroism

**Figure 2.**
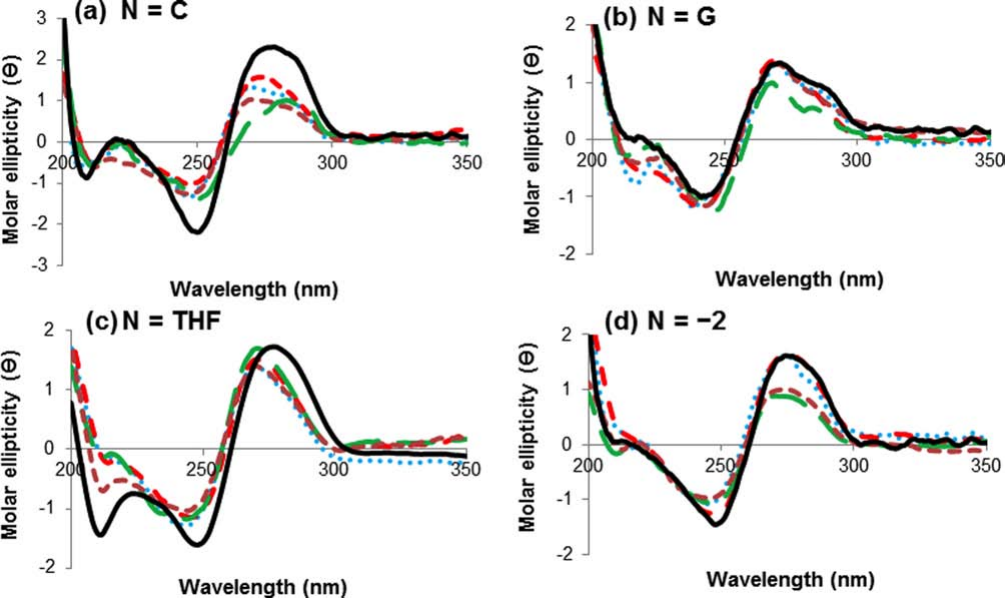
CD spectral overlays of *Nar*I(12) duplexes with X = G (solid black lines), X = FurG (dashed red lines), X = PhG (dashed brown lines), X = CNPhG (dotted blue lines) and X = QG (dashed green lines). All spectra of duplexes (6 μM) were recorded in 50 mM sodium phosphate buffer, pH 7, with 0.1 M NaCl at 10ºC.

The *Nar*I(12) duplexes were analysed using CD spectroscopy to determine the impact of the adduct on the global tertiary structures. All duplexes showed characteristics of B-form DNA (67), with positive (275 nm) and negative (244 nm) sigmoidal CD curves and a crossover at ∼260 nm (Figure 2). For *Nar*I(12) paired opposite C, modified duplexes displayed significant decreases in the positive CD band at 275 nm (Figure 2a). This spectral change has been observed during DNA denaturation (67), and in conditions that increase the winding angle of the DNA base pairs (68), with less optimized stacking interactions. Conversely, the amplitudes of the bands at 240 and 275 nm in the CD spectra of *Nar*I(12) duplexes in which the adducts were paired with G or THF were changed minimally from unmodified duplexes. This lack of perturbation suggests the global and local DNA structure at the site of the lesion is retained from the unmodified helix. For *Nar*I(12) hybridized with the truncated 10mer oligonucleotide, amplitudes at 275 nm were variable, with FurG and CNPhG adducted DNA and the unmodified duplex exhibiting similar peak heights in the CD spectra. The amplitude at 275 nm was diminished for PhG and QG modifications. In all spectra, it was not possible to resolve ellipticities due to the C8-aryl-dG modified bases.

#### DFT calculations and MD simulations

**Figure 3.**
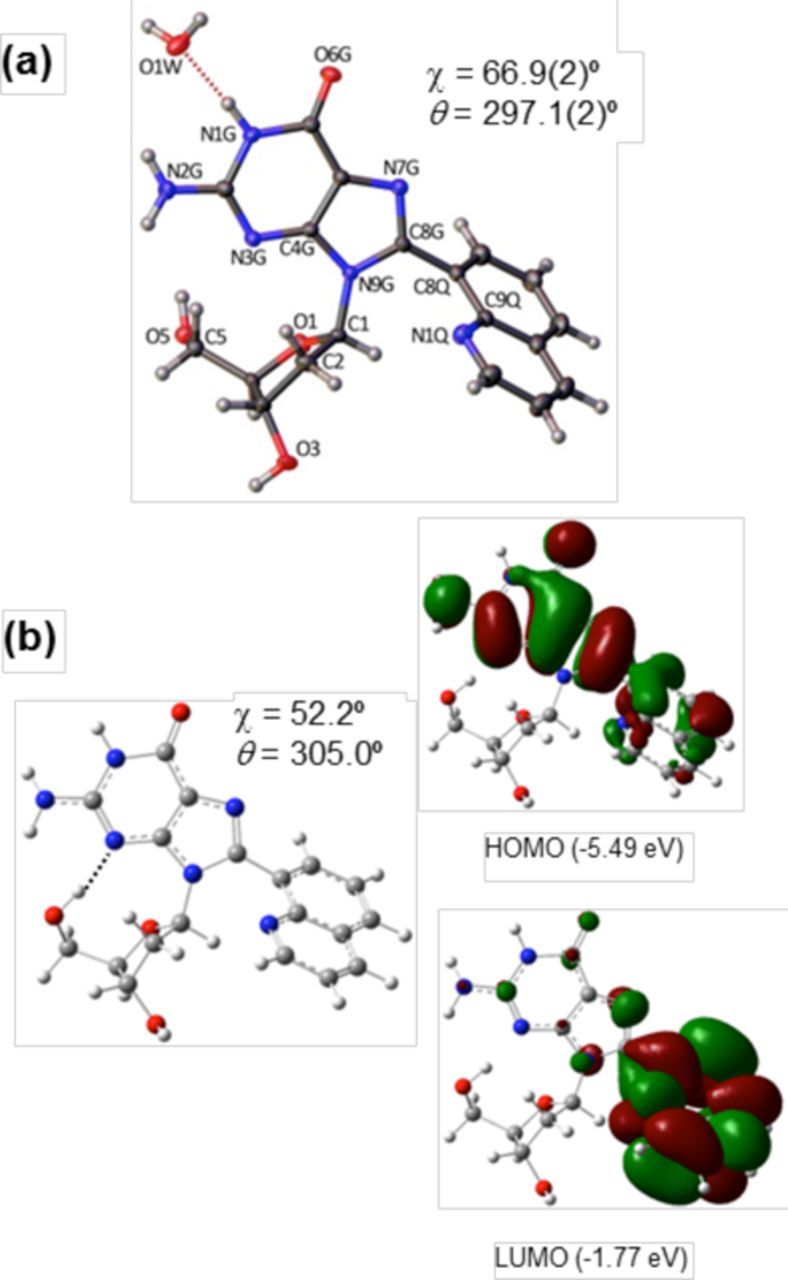
(a) ORTEP projection of QG monohydrate with torsion angles *χ* and *θ* provided. (b) B3LYP/6–31G(d) global minima of QG (O5′-H•••N3 H-bond, *χ* and *θ* provided) and orbital density plots with HOMO and LUMO energy levels (eV).

Structural parameters for the free nucleoside adducts were determined using DFT calculations at the B3LYP/6–31G(d) level. Fully optimized *anti* and *syn* minima and transition states on the potential energy surface for the PhG and QG nucleoside adducts are displayed in Supplementary Figures S1 and S2, whereas the corresponding data for FurG (49) and CNPhG (50) is published elsewhere. C8-Aryl ring size governs the degree of *syn* preference (*E*_anti_-*E*_syn_ = 19.3 for FurG versus 29.5 kJ mol^−1^ for QG) and the degree of twist (*θ*) between the nucleobase and the C8-aryl substituent (*θ* = 343.8º for FurG versus 305.6º for QG, Table 2). The molecular structure of QG observed in the crystal (ORTEP projection (69)) and the lowest-energy structure of QG calculated with DFT (B3LYP/6–31G(d) level) are compared in Figure 3. The conformations are in agreement (*syn*) with only minor variations in torsion angles *χ* and *θ*. The ability of the calculations to model the solid-state structure of QG, supports that the DFT data (Table 2) accurately reflects structural differences in the nucleoside adducts.

**Table 2.** Structural and optical properties of C8-aryl-dG nucleoside adducts.

Adduct	*E_anti_*-*E_syn_*^a^	*χ*/*θ*^b^	*μ*	*λ*_ex_,*λ*_em_^c^ (*Φ_fl_*^d^)	*λ*_ex_,*λ*_em_ (*Φ_fl_*)	*λ*_ex_,*λ*_em_ (*Φ_fl_*)
	kJ mol^−1^	Degrees	Debye	H_2_O^e^	CH_3_CN	CHCl_3_
FurG^f^	19.3	50.1/343.8	4.3	292, 384 (0.49)	292, 371 (0.18)	298, 366 (0.03)
PhG	25.1	66.8/41.2	4.8	277, 395 (0.44)	292, 384 (0.49)	289, 373 (0.22)
CNPhG^g^	26.9	68.4/37.9	8.9	308, 468 (0.04)	327, 454 (0.43)	325, 424 (0.35)
QG	29.5	52.2/305.6	4.3	313, 407 (0.03)	315, 384,510 (0.05)	318, 468 (0.19)

^a^Optimized at the B3LYP/6–31G(d) level, with values corresponding to a *syn*-conformation with a relative energy of 0 kJ mol^−1^.

^b^The dihedral angle *χ* (O4′C1′N9C4) defines the glycosidic bond orientation to be *anti* when *χ* = 180 ± 90º or *syn* when *χ* = 0 ± 90º; *θ* (∠(N9C8C10C11) for PhG, CNPhG and QG and ∠(N9C8C10O11) for FurG) defines the degree of twist between the nucleobase and the C8-aryl group.

^c^Excitation and emission maxima in nm.

^d^Determined using the comparative method with quinine bisulfate in 0.5 M H_2_SO_4_ (*Φ_fl_* = 0.55).

^e^Determined in aqueous 10 mM MOPS buffer, pH 7, *μ* = 0.1 M NaCl.

^f^Optical data in H_2_O and energy calculations for FurG taken from (49).

^g^Optical data in H_2_O and energy calculations for CNPhdG taken from (50).

MD simulations were carried out using different starting conformations of the (*anti* and *syn*) adducts at the G_3_-position in the *Nar*I(12) recognition sequence opposite C, G, THF or −2. The resulting conformations were ranked with respect to the calculated free energies. Detailed results from the MD simulations and the associated free energy rankings are provided in Supplementary Data.

#### Adducts paired against C

**Figure 4.**
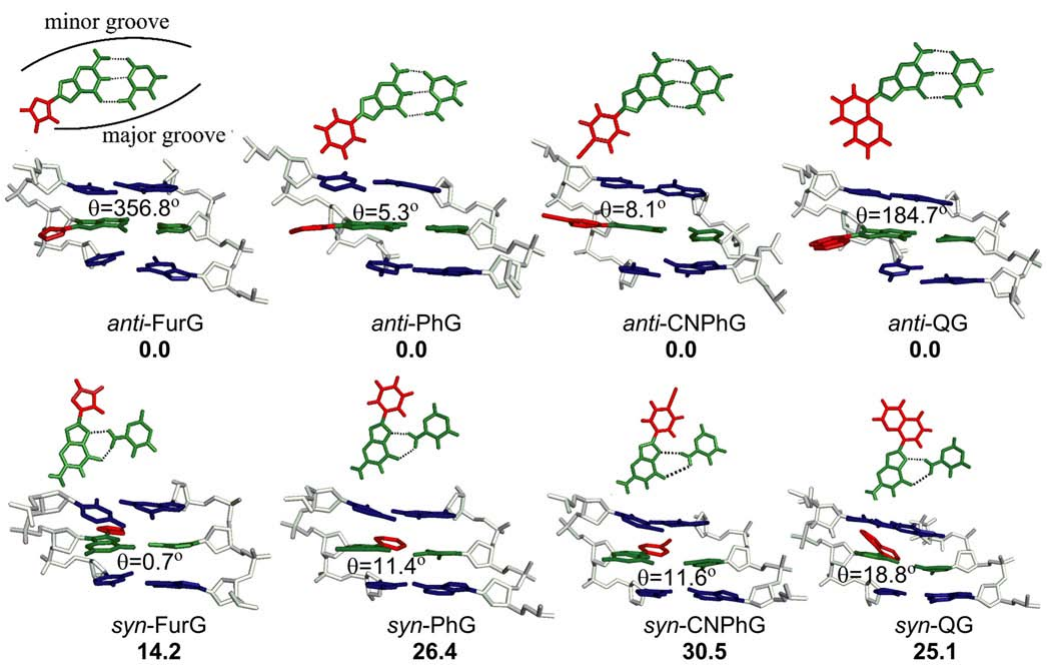
Representative structures corresponding to lowest energy *anti* and *syn* conformations for the studied adducts paired against C. The relative free energies (kJ mol^−1^) of two competing conformations are provided in bold. H-Bonding contacts are indicated by dashed lines.

Analysis of the MD trajectories reveals that the *anti* orientation of all adducts allows them to maintain W-C H-bonding with the complementary C (more than 98% occupancy of H-bonds; Supplementary Table S2 and SI), which places the C8-moiety in the major groove (Figure 4, Supplementary Figures S18a and b, S22a and b, S24a and b and S26a and b). However, depending on the size of the C8-moiety and the orientation of the *θ* angle, the *syn* conformations have different structural characteristics. Specifically, the *syn* orientations of all adducts can adopt two different conformations (details in Supplementary Data), out of which only one maintains Hoogsteen H-bonding with the opposing C thus displaying the W-type conformation (Figure 4, Supplementary Figures S18c and d, S22b, S24b and S26c and d). However, except for QG, the S-type structure was not observed for any other adduct. In addition, the observed S structure of QG was ranked energetically higher than the W conformation (details in Supplementary Data).

The calculated free energies (Supplementary Table S3) suggest that in the DNA duplex PhG, CNPhG and QG favour the *anti* conformation over the *syn* conformation(s), by at least 25 kJ mol^−1^. This preference indicates that the loss of W-C H-bonding upon adopting the *syn* conformation is not adequately compensated by relatively weaker Hoogsteen H-bonding and/or stacking interactions with the C8-moiety. On the other hand, the smaller *anti*/*syn* energy difference (14 kJ mol^−1^) calculated for FurG indicates greater accessibility of the *syn* conformation for this adduct (Figure 4). The enhanced stability of the *syn* conformation when FurG is paired opposite C can be accounted for with the greater persistence of two Hoogsteen H-bonds over the simulation time (∼70% occupancy of both bonds) compared to PhG (64% and 59% occupancy), CNPhG (65% and 44% occupancy) and QG (77% and 53% occupancy, Supplementary Table S2).

#### Adducts paired against G and THF

**Figure 5.**
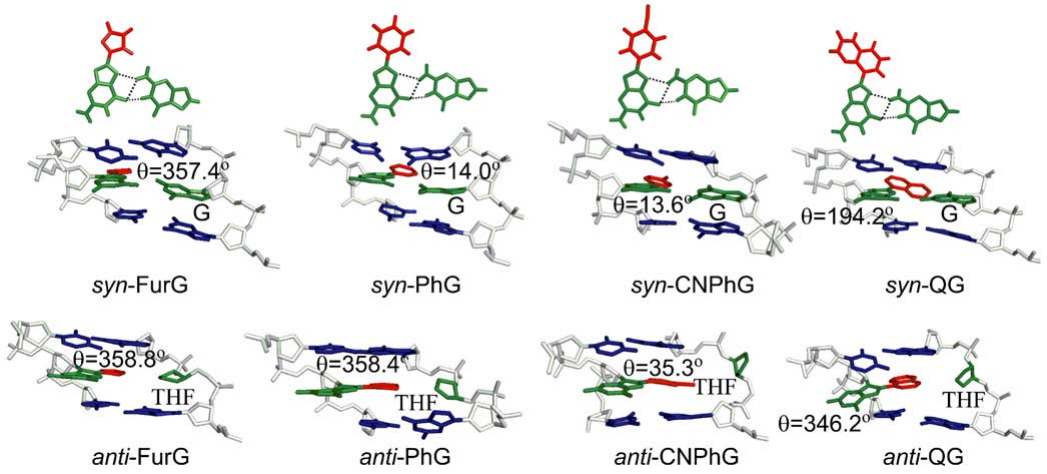
Representative structures corresponding to lowest energy *syn* conformations for the studied adducts paired against THF and G. H-Bonding contacts are indicated by dashed lines.

In order to understand the structural characteristics of guanine mismatch stabilization by the C-linked adducts, adducts in the *syn* orientation were paired against *anti-*G, while the *anti* orientations of the adducts were paired against *syn*-G to allow H-bonding interactions with the opposing G. Although both *syn* and *anti* conformations of the adducts accommodate Hoogsteen H-bonding with the opposing G (Supplementary Figures S19, S22candd, S24c and d and S27), the calculated free energies obtained from the simulations of the ensemble of structures indicate that when paired opposite G, all four adducts prefer a *syn* conformation by 24–113 kJ mol^−1^ depending on the identity of the C8-substituent (Figure 5, Supplementary Table S3).

The adducts in their *syn* and *anti* conformations were also paired against THF (Figure 5) as a model abasic site (62). In the *anti* conformations of all adducts, the C8-subsituent remains in the major groove and has little possibility of forming additional stacking interactions with the flanking bases (Supplementary Figures S20a and b, S23a, S25a and S28a and b). On the other hand, when paired in their *syn* conformations against THF, the adducts have greater propensity to intercalate their C8-substituents between the flanking base pairs within the DNA helix (Figure 5, Supplementary Figures S20c and d, S23b, S25b and S28c and d). The calculated relative free energies indicate that all adducts prefer the *syn* conformation against THF, mainly due to additional stacking interactions with the flanking bases.

#### Adducts paired against −2

**Figure 6.**
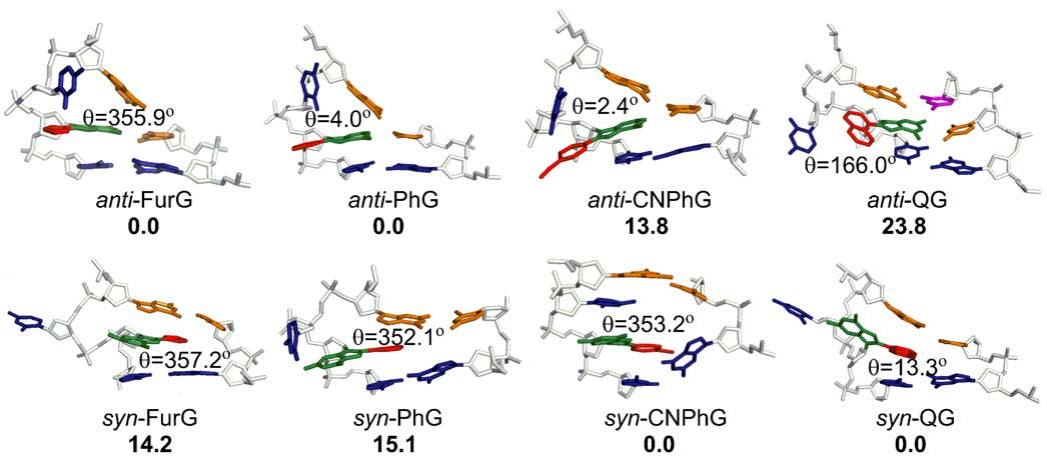
Representative structures corresponding to lowest energy *anti* and *syn* conformations for the studied adducts paired against −2. The relative free energies (kJ mol^−1^) of two competing conformations are provided in bold.

The *Nar*I(12) strands containing the adducts in the *syn* and *anti* conformations were also paired against the truncated 10-mer strand (−2, see Table 1 for 10-mer sequence). The free energy calculations indicate that FurG and PhG prefer the *anti* conformation within the 2-base bulge (Figure 6). However, the *syn* conformation ranks lower in energy in the case of CNPhG and QG (Figure 6, Supplementary Table S3). FurG and PhG adopt the *anti* conformation because it is stabilized by H-bonds between the C that is opposite the adduct and 5′ with respect to the bulge (Figure 6). Although such H-bonding interactions are also present in the *anti* conformation of CNPhG-adducted DNA, the corresponding *syn* conformation gains stability from additional stacking interactions between the adduct and the unpaired C on its 5′-side (Figure 6). QG prefers the *syn* conformation due to optimal stacking interactions between the C8-quinolyl moiety and the paired C at its 3′-side, and the paired G at its 5′-side (highlighted in orange, Figure 6). The unpaired C present at the 5′-side of QG is in an extrahelical position and does not participate in H-bonding or π-stacking interactions (5′-C highlighted in blue, Figure 6).

#### Fluorescence spectroscopy

The emissive properties of the C-linked C8-aryl-dG adducts were employed to further probe their conformational preferences in the *Nar*I(12) duplexes. In the nucleoside form, CNPhG (50) possess donor-acceptor (D-A) character and exhibits fluorescence that is quenched in water (i.e. *Φ_fl_* = 0.04 in H_2_O versus 0.43 in CH_3_CN, Table 2) and sensitive to solvent polarity (i.e. *λ*_em_ = 468 nm in H_2_O versus 424 nm in CHCl_3_, Table 2). The QG adduct also exhibits D-A character, as exemplified by the orbital density plots (Figure 3) in which the LUMO has significantly greater density on the quinolyl moiety, which is consistent with D-A character (50). The QG adduct also exhibited dual emission in CH_3_CN at 384 and 510 nm following excitation at 315 nm (Table 2). A rationale for the emissive properties of QG in H_2_O, CH_3_CN and CHCl_3_ is provided in Supplementary Figure S34. In contrast, FurG and PhG lack D-A character and are strongly emissive in water (*λ*_em_ ∼ 390 nm, *Φ_fl_* ∼ 0.5) but exhibit emission wavelengths that are weakly sensitive to solvent polarity. Since the relative exposure of the adduct to the bulk solvent (water) will depend on its conformation in DNA (Figure 1a), the unique fluorescent properties of these adducts will help determine adduct positioning within the *NarI*(12) duplexes.

Emission and excitation parameters of the adducted *Nar*I(12) oligonucleotides in the single-strand state, and upon hybridization to the various complementary strands are given in Table 3. Fluorescence emission spectra of the single strands and duplexes are displayed in Supplementary Figure S35. For FurG and PhG the wavelengths of emission in the duplex structures did not differ significantly from the single-strand emission wavelength (Table 3, Δ*λ*_em_). These characteristics are not informative for predicting *syn*/*anti* conformational equilibria. However, the relative emission intensity (*I*_rel_) in the duplex versus the single strand for both FurG and PhG exhibit changes that are informative. Specifically, both adducts display quenched emission intensity when paired opposite C and G. Free nucleosides that lack D-A character exhibit quenched emission with increased solvent viscosity (49). Compared to the single-strand environment, the adducts will experience increased rigidity in the duplex due to increased π-stacking and H-bonding of the G moiety to the opposing base in the helix. Many fluorescent nucleobase analogues show quenched emission in oligonucleotides and duplex structures that have been ascribed to base-stacking effects (70,71).

**Table 3.** Photophysical parameters of C8-aryl-dG modified *Nar*I(12).

*X*	*N*	*λ*_ex_	Δ*λ*_ex_	*λ*_em_	Δ*λ*_em_	Δ*ν*	*I*_rel_
		(nm)^a^	(nm)^b^	(nm)	(nm)	(cm^−1^)^c^	(em)^d^
FurG	/^e^	312	/	381	/	5804	/
FurG	C	314	2	381	0	5600	0.45
FurG	G	312	0	382	1	5873	0.48
FurG	THF	305	-7	383	2	6677	2.21
FurG	−2	306	-6	380	-1	6364	2.97
PhG	/	290	/	395	/	9166	/
PhG	C	302	12	394	-1	7732	0.38
PhG	G	300	10	391	-4	7758	0.28
PhG	THF	297	7	398	3	8544	0.79
PhG	−2	296	6	395	0	8467	0.80
CNPhG	/	334	/	464	/	8388	/
CNPhG	C	332	-2	462	-2	8476	1.04
CNPhG	G	335	1	462	-2	8206	2.28
CNPhG	THF	335	1	453	-11	7776	2.00
CNPhG	−2	333	-1	457	-7	8148	3.92
QG	/	321	/	477	/	10 189	/
QG	C	322	1	475	-2	10 003	0.41
QG	G	324	3	480	3	10 031	1.55
QG	THF	327	6	467	-10	9168	2.24
QG	−2	329	8	468	-9	9027	4.92

^a^All spectra of single-strand *Nar*I(12) and duplexes (6 μM) were recorded in 50 mM sodium phosphate buffer, pH 7, with 0.1 M NaCl at 10ºC.

^b^Change in excitation or emission maximum for duplex versus single strand.

^c^Stokes’ shift (Δ*ν*) is calculated as (1/*λ*_ex_ – 1/*λ*_em_).

^d^*I*_rel_ = *I*_duplex_/*I*_single-strand_

^e^/ indicates optical properties of the modified base in the single strand.

When paired opposite THF and −2, FurG exhibited increased emission intensity (*I*_rel_ ∼ 2–3), while PhG displayed similar emission intensity to the single strand (*I*_rel_ ∼ 0.8). In these duplexes, the emissive nucleobase should have greater flexibility due to the lack of H-bonding interactions with an opposing base. In the −2 duplex, MD simulations favour an *anti* conformation for both FurG and PhG (Figure 6) and indicate diminished π-stacking interactions compared to the full-length complement. Thus, the lack of H-bonding interactions coupled with reduced π-stacking in these duplex structures would decrease adduct rigidity and lead to enhanced emission intensity.

In the single strand, CNPhG emits at 464 nm with a Stokes’ shift of 8388 cm^−1^ (Table 3). Paired opposite C, CNPhG exhibits emission intensity almost identical to the single strand (*I*_rel_ = 1.04) with a comparable Stokes’ shift. These observations are consistent with exposure of the cyanophenyl moiety to the aqueous environment. For CNPhG paired opposite G and THF, the MD simulations strongly favour the *syn* conformation. CNPhG can form Hoogsteen H-bonding with the opposing *anti*-G, which positions the cyanophenyl moiety in the minor groove. Opposite THF, the absence of an opposing base locates the C8-moiety towards the interior of the helix and enhances stacking interactions with the neighbouring bases (Figure 5). Thus, CNPhG paired opposite THF would be expected to exhibit a greater blue-shift in emission wavelength compared to CNPhG paired opposite C and G, as it is more sequestered from the aqueous environment. Inspection of the photophysical data (Table 3) is consistent with the MD simulations. The Stokes’ shift for CNPhG paired opposite THF is strongly diminished due to the blue-shift in emission wavelength (Δ*λ*_em_ = −9 nm compared to CNPhG paired opposite C and G). Finally, for CNPhG paired opposite −2, the calculations favour the *syn* conformation, which places the cyanophenyl moiety in the interior of the helix (Figure 6). The emission wavelengths for CNPhG paired opposite −2 are similar to the corresponding wavelengths for *syn*-CNPhG paired opposite THF (Table 3). However, CNPhG is more emissive within the 2-base bulge (*I*_rel_ = 3.92), due to the loss of effective π-stacking interactions compared to the full-length duplex.

In the single strand, QG emits at 477 nm in *Nar*I(12) with a Stokes’ shift of 10 189 cm^−1^ (Table 3). This emission was ascribed to a charge-transfer state. Paired opposite C, the emission of QG was strongly quenched (*I*_rel_ = 0.41) with relatively little change in Δ*ν* compared to the single strand. The calculations strongly favour the *anti* conformation for QG paired with C (Figure 4) and the fluorescence data is consistent with exposure of the quinolyl moiety to the aqueous environment, which quenches emission intensity (Table 2). With QG opposite G, MD simulations predict a *syn* conformation with the bulky quinolyl group positioned in the minor groove (Figure 5). Opposite THF (Figure 5) and −2 (Figure 6), QG is expected to adopt the *syn* conformation with the quinolyl moiety within the π-stack sequestered from the aqueous solvent environment. In these duplexes, the emissive QG exhibits a significant decrease in Stokes’ shift (Table 3) due to the blue-shift in emission wavelength, which is consistent with its placement in a non-polar environment.

### Primer extension of *Nar*I(22)

#### Extension by Klenow exo^−^(Kf^−^)

**Figure 7.**
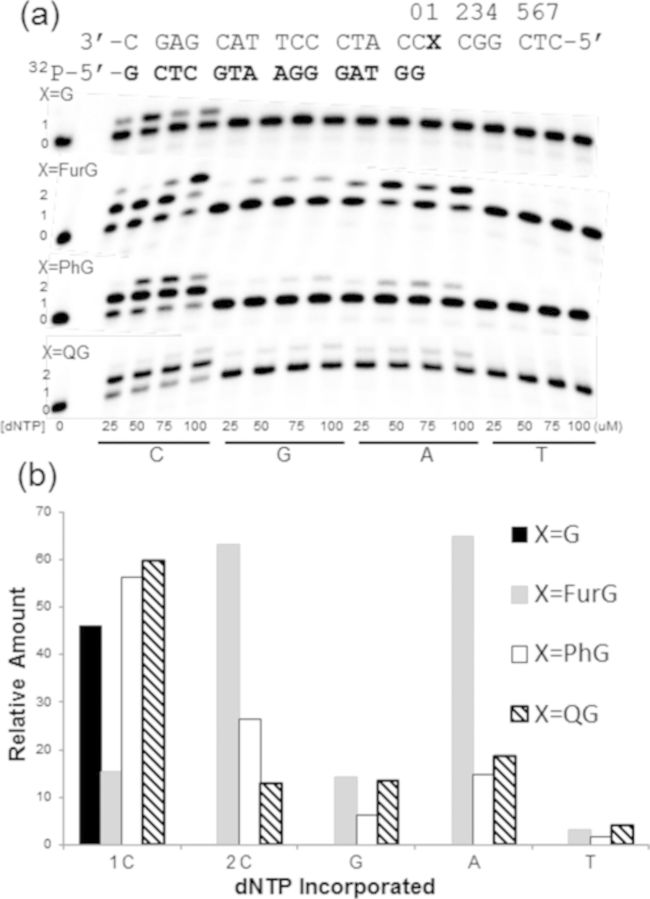
(a) Single-nucleotide incorporation primer extension assays with increasing concentrations of individual dNTPs as indicated under each lane. 1 nM Kf^−^ was incubated with undamaged (X = G) template, while 10 nM Kf^−^ was incubated with adducted (X = FurG, PhG or QG) templates for 1 h. (b) Highest relative frequency of nucleotide incorporation over the range of dNTP concentrations (25–100 μM), X = G (solid black), X = FurG (grey), X = PhG (white) and X = QG (black lined).

To determine the miscoding potential of C-linked C8-dG adducts, single-nucleotide insertion assays were performed with the *Nar*I(22):15mer template:primer and Kf^−^ in the presence of 25–100 μM individual dNTP (Figure 7). For the unmodified *Nar*I(22) template (X = G), only the correct base C was incorporated. However, for the *Nar*I(22) template modified to contain the smallest adduct (X = FurG), the polymerase inserted C twice. Thus, in the presence of 100 μM dCTP, we observed 63% of the product containing two C's, relative to 15% of the single C insertion product and 22% unreacted primer. When extension past N-linked C8-dG adducts at the G_3_-position of *Nar*I was examined previously (33), incorporation of a second C has been categorized as a so-called two-base slippage phenomenon and implies pairing of CC with GG one position removed to the 5′-side of the adduct site (positions 3 and 4 in the template strand, Figure 7a). A similar magnitude incorporation of one A (65%) and small amounts of G (14%) and T (3%) were also observed. For the templates containing X = PhG and QG, the two-base slippage phenomenon with incorporation of two C bases was also observed, but to a lesser degree (PhG, 26%; QG, 13%). PhG and QG also diminished the extent of misincorporation of A by Kf^−^ (Figure 7b).

**Figure 8.**
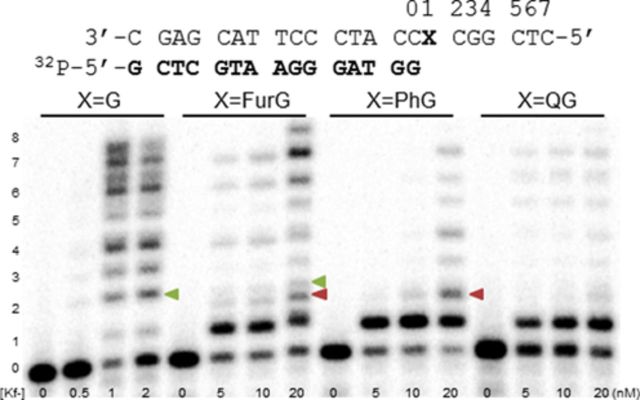
Full-length extension of *Nar*I(22):15mer template:primer (X = G, FurG, PhG or QG) by Kf^−^. Increasing concentrations, indicated under each lane, of Kf^−^ was incubated with the DNA substrates for 1 h in the presence of 25 μM of each dNTP. Green triangles indicate base incorporation products that migrate with unmodified template, while red triangles indicate base incorporation products with different mobilities on the gel.

In the presence of all four dNTPs (Figure 8), extension past the C8-aryl-dG adducts became increasingly blocked as the size of the C8-aryl substituent increased. For each adduct, extension one-base past the adduct site was clearly more hindered than incorporation opposite the adduct. This observation is in agreement with previous reports that bulky N-linked C8-dG adducts provide significant kinetic barriers to extension by Kf^−^ following base insertion opposite the lesion (72). For example, relative extension frequencies for inserting one base after a C8-AF-G:C or C8-AAF-G:C at the primer terminus are ∼0.032 and 1.4 × 10^−6^ relative to a value of 1 for G:C (72). For the C-linked C8-dG adducts, the smallest adduct FurG was extended most efficiently by Kf^−^, resulting in a +7 band for the full-length 22mer extension product (Figure 8). A +8 band was also observed and was ascribed to blunt-end extension, which is independent of the polymerase employed and nature of the template strand (modified or unmodified) (73). For extension past FurG, additional partial extension bands were also detected that migrated with different mobilities than those arising from the control unmodified *Nar*I(22) template. For example, at the second incorporation site the unmodified template exhibits a single product indicated by the green triangle (Figure 8). However, at the same position, two products were detected for extension past FurG. The major product labelled with the red triangle migrated faster on the gel than the product observed for the unmodified template. The same product peak (labelled with red triangle) was also detected for extension past PhG. The observed mixture of products for extension past FurG and PhG suggests that these lesions promote both error-free and error-prone extension by Kf^−^ (33).

#### Translesion synthesis by Dpo4

**Figure 9.**
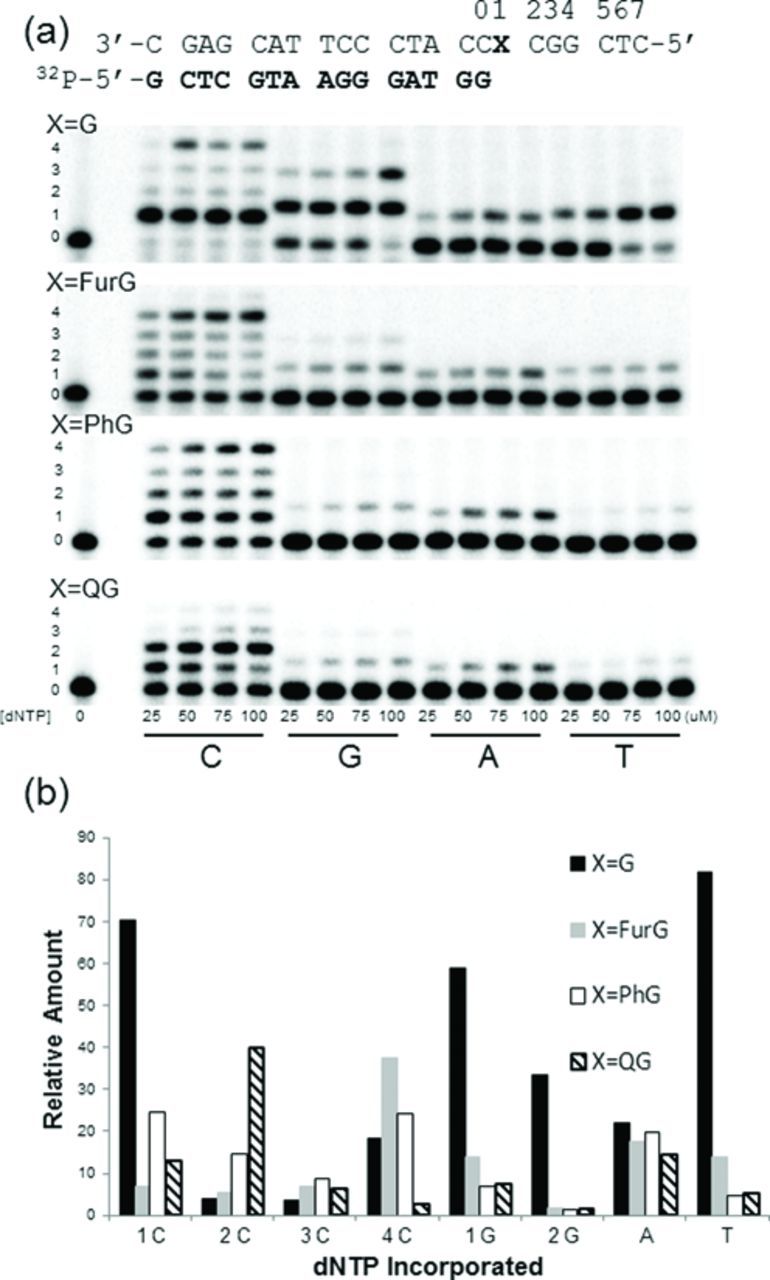
(a) Single-nucleotide incorporation primer extension assays with increasing concentrations of individual dNTPs as indicated under each lane. Dpo4 (10 nM) was incubated with the undamaged (X = G) as well as the adducted (X = FurG, PhG QG) *Nar*I(22):15mer template:primer for 30 min. (b) Highest relative frequency of nucleotide incorporation over the range of dNTP concentrations (25–100 mM), X = G (solid black), X = FurG (grey), X = PhG (white) and X = QG (black lined).

Given that the C-linked C8-dG adducts strongly block extension by the high-fidelity Kf^−^, it was desirable to examine replication of the *Nar*I(22) templates using Dpo4 as a model lesion-bypass DNA polymerase. In single-nucleotide insertion assays (Figure 9), Dpo4 exhibited low fidelity with the unmodified *Nar*I(22) template (X = G), on the basis of significantly misincorporating bases opposite G. The single-base insertion products and their abundance relative to unreacted primer were: G (59%), or GG (34%), T (82%) or A (22%), in the presence of dGTP, dTTP or dATP, respectively. When provided only with the correct option dCTP, the polymerase primarily inserted a single C (71%). However, bands for incorporation of 2, 3 or 4 C bases were also observed. The low fidelity observed for Dpo4, even with the unmodified *Nar*I(22) template, is consistent with its established low geometric selection for correct base pairs (74).

The extent of polymerase misincorporation of G and T decreased considerably between the modified *Nar*I(22) templates compared to the unmodified control (Figure 9b). Weak bands for misincorporation of G (also weak band for a second G), A and T were observed (5–17%). The presence of C-linked C8-aryl-dG adducts in the template also promoted a large extent of multiple incorporations of C by Dpo4, suggesting promotion of slippage compared to the unmodified template. Both FurG and PhG promoted incorporation of the 4 C's, while the bulkier QG adduct promoted incorporation of two C's.

**Figure 10.**
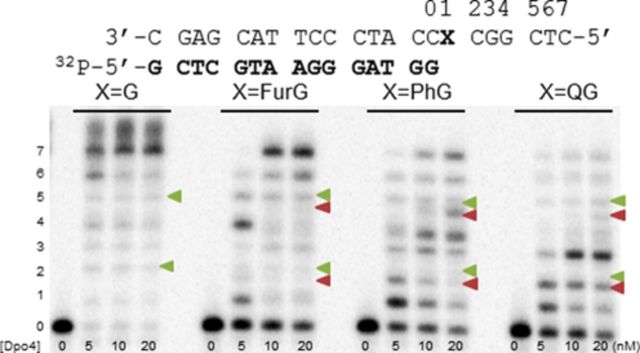
Full-length extension of *Nar*I(22):15mer template:primer (X = G, FurG, PhG or QG) by Dpo4. Increasing concentrations of Dpo4, indicated under each lane, were incubated with the substrates for 30 min in the presence of 25 μM of each dNTP. Green triangles indicate base incorporation products that migrate with unmodified template, while red triangles indicate base incorporation products with different mobilities on the gel.

In the presence of all four dNTPs (Figure 10), Dpo4 was able to extend past the lesion site, whereas Kf^−^ was clearly blocked (Figure 8). Full-length extension was observed for the modified template containing FurG. However, PhG and especially QG inhibited the ability of Dpo4 to extend the primer to full-length 22mer product. For the bulkiest QG lesion, primer extension was strongly blocked following insertion of three bases to afford an 18mer product (33% for +3 incorporation band). As noted in full-length extension by Kf^−^ (Figure 8), mutagenic extension by Dpo4 (33) was indicated by additional truncation bands that migrated with different mobilities (highlighted by red triangles) than the truncation bands arising from the unmodified *Nar*I(22) template (highlighted by green triangles).

## DISCUSSION

### Conformational preferences of C-linked C8-aryl-dG adducts within *Nar*I(12) duplexes

The MD simulations predict that all C-linked C8-aryl-dG adducts exhibit an *anti* preference paired opposite C (Figure 4), despite the strong *syn* preference of the free nucleosides (Table 2). This conformation places the C8-aryl ring in the major groove exposed to the aqueous environment. The fluorescence data for CNPhG and QG support this model, as both D-A adducts exhibit emissive characteristics within the duplexes that are similar to the single strand, suggesting that both adducts are exposed to water. Thus, increasing C8-aryl ring size decreases duplex stability when the adducts are paired opposite C (Table 1) because the larger adducts are more lipophilic and have greater intrinsic *syn* preference (Table 2). Therefore, the smallest adduct (FurG) is the most flexible and can better access the *syn* conformation when paired opposite C (Figure 4). The low-energy *syn* conformations are W-type (Figure 1a) with the C8-aryl moiety in the minor groove (Figure 4), which are structurally similar to the favoured *syn* conformations of the adducts mismatched with G (Figure 5). In both duplexes (matched and mismatched) the C8-aryl group is either too small or too twisted with respect to the nucleobase to effectively intercalate into the DNA helix and extrude the opposing base into an extrahelical position. This lack of effective intercalation is also reflected in the inability of the adducts to stabilize the duplexes when paired opposite THF and within the 2-base bulge (Table 1). Our results demonstrate that the conformational features of the C-linked C8-aryl-dG adducts within the *Nar*I sequence clearly differ from those established for the polycyclic N-linked C8-dG counterparts (5,19–22).

### Rationale for Dpo4 slippage mediated by C-linked C8-aryl-dG adducts

**Figure 11.**
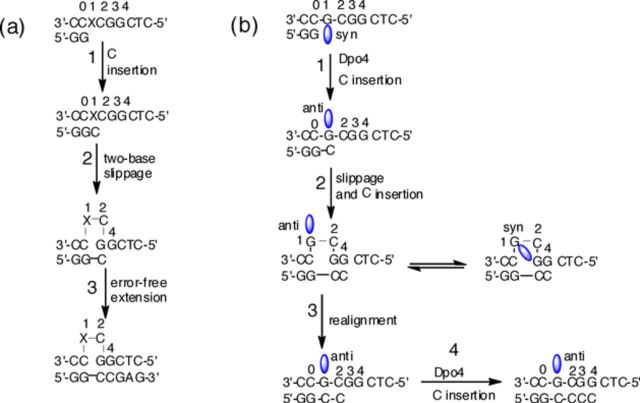
(a) Proposed 2-base slippage mechanism for frameshift mutation induced by N-linked C8-dG adducts in the reiterated X-site of the *Nar*I sequence. (b) Proposed Dpo4 slippage mechanism induced by C-linked C8-dG adducts within the *Nar*I sequence.

At the reiterated G_3_-position of the *Nar*I sequence the current model for 2-base slippage (Figure 11a) induced by N-linked C8-dG adducts involves initial insertion of C opposite the C8-dG adduct (X, step 1), that is followed by 2-base slippage (step 2) and base-pairing of the inserted C with G at position 3 in the template strand (Figure 11a, inserted primer bases are highlighted in bold and numbering of the template strand is provided) (48). In this model, the slowed progression of DNA replication due to the presence of damaged DNA bases is thought to permit the inserted C to switch base-pairing positions and generate the 2-base bulged SMI. In a *syn* conformation, the aryl moiety of the N-linked C8-dG adduct can intercalate into the truncated duplex to stabilize the 2-base bulged SMI (63–66). The polymerase extends the SMI to produce the 2-base deletion product (step 3) that is two bases shorter than the expected full-length extended oligonucleotide (31).

For the C-linked C8-aryl-dG adducts at the G3-position of *Nar*I(22), Dpo4 inserted multiple C bases (Figure 9), which strongly suggested a slippage mechanism as outlined in Figure 11b. In the starting *Nar*I(22):15mer template:primer, we anticipate the adducts to be present in a *syn* conformation, given their strong *syn* preference (Table 2) and that there is no opposing base (MD simulations and emission spectra favour the *syn* conformation for the adducts paired opposite THF in *Nar*I(12), Figure 5, Supplementary Table S3). Insertion of C (step 1) requires that the C-linked C8-aryl-dG adduct adopt the *anti* geometry, which forces the polymerase to flip the adduct from its stable *syn* conformation into the energetically destabilized *anti* conformation. The barrier for this process is expected to depend on C8-aryl ring size (see *E_anti_*-*E_syn_*, Table 2) and this conformational change is expected to stall DNA replication compared to replication of the unmodified template. The stall in replication caused by the C-linked C8-aryl-dG adducts would allow for slippage and production of the 2-base bulge SMI (step 2), as proposed for the N-linked C8-dG adducts (Figure 11a) (48).

Following 2-base bulge formation, the polymerase can insert a second C opposite G at position 4 in the template strand (included in step 2, Figure 11b). The resulting 2-base bulged duplexes are anticipated to exist as an equilibrium mixture of the *anti* or *syn* conformations. Our MD simulations suggest that both FurG and PhG energetically favour the *anti* geometry within the 2-base bulge, while the bulkier QG favours the *syn* conformation (Figure 6, Supplementary Table S3). Thus, we propose that the SMI with FurG and PhG can undergo a realignment process (step 3) to permit additional C incorporation by Dpo4 across from positions 3 and 4 in the template strand (step 4). The MD-simulated structures of the 2-base bulged duplexes with FurG and PhG suggest that realignment would be facile because both adducts are in the correct *anti* geometry to W-C base pair with C in the extended primer and neither adduct stabilizes the 2-base bulge (Table 1). This proposal provides a rationale for incorporation of four C bases in the single-nucleotide incorporation assays by Dpo4 for X = FurG and PhG (Figure 9). The end result is the formation of a C:C mismatch at position 2 of the template strand. Dpo4 has a tendency to generate C:C mismatches, especially when the C in the template has a 5′-neighbouring G (74), as present in *Nar*I(22).

In contrast, realignment of the primer:template when the adduct QG is present is anticipated to be disfavoured because QG energetically favours the *syn* conformation within the SMI (Figure 6, Supplementary Table S3). Thus, QG is not expected to undergo realignment, as the QG adduct would need to flip from its favoured *syn* conformation in the SMI into an unstable *anti* geometry. Consistent with this model, only two C bases were inserted by Dpo4 under single-nucleotide incorporation conditions with X = QG (Figure 9), which is classical evidence for 2-base slippage (31).

### Biological implications: C- versus N-linked C8-G adducts

The vast literature concerning the N-linked C8-dG adducts demonstrate that all of the derivatives with potent mutagenicity have polycyclic structures (75). None of the single-ringed aniline derivatives are potent mutagens (76). Polycyclic N-linked adducts, such as C8-AAF-dG (19), C8-AF-dG (20,21) and C8-IQ-dG (22), can adopt the S-type conformation (Figure 1a) when paired opposite C in duplex DNA, which is regarded as a pro-mutagenic conformation (5,6). For the N-linked C8-aniline-dG adduct the aniline ring system is too small to effectively intercalate and so the base readily adopts the *anti* conformation when paired opposite C in duplex DNA and favours the B-type structure with minimal distortion to the helix (18). The B-type structure is also favoured for the C-linked C8-aryl-dG adducts paired opposite C (Figure 4). However, C-linked C8-dG adducts possess a strong *syn* preference (Table 2) and C8-aryl ring size governs their conformational flexibility between *syn* (W-type) and *anti* (B-type) structures (Figure 4). In the present study, the smallest adduct tested (FurG) exhibited the easiest accessibility to the W-type structure (Figure 4). Consequently, FurG produced the greatest levels of A and G incorporation by Kf^−^ (Figure 7) and G incorporation by Dpo4 (Figure 9), as it can more readily adopt a pro-mutagenic *syn* conformation in which the Hoogsteen H-bonding face of G can base pair with G (Figure 5) and A (77). Thus, the *syn* conformational preference of C-linked C8-dG adducts provides a rational for their ability to induce G → T and G → C transversion mutations (9,39).

Polycyclic N-linked C8-dG adducts at the reiterated G_3_-position of the *Nar*I sequence are known to generate −2 frameshift mutations (31,48) which correlates with their ability to stabilize a 2-base SMI (63–66). In contrast to their N-linked counterparts, none of the C-linked C8-dG adducts studied herein stabilized the SMI compared to the unmodified control (Table 1). However, a key mechanistic feature of slippage is the ability of the lesion to stall progression of DNA synthesis (48). The C-linked C8-dG adducts effectively blocked full-length extension by Kf^−^ (Figure 8), which suggests recruitment of lesion-bypass polymerases, such as Dpo4. Extension by Dpo4 resulted in a modified slippage phenomenon that suggested a realignment process to produce a C:C mismatch distal to the adduct site (Figure 11b). The tendency of C-linked C8-aryl-dG adducts to promote slippage at reiterated sequences during translesion synthesis by Y-family polymerases may also represent a basis for their mutagenic properties.

## CONCLUSIONS

The current study has allowed us to conclude the following: (i) at the reiterated G_3_-position of a 12mer *Nar*I oligonucleotide (*Nar*I(12)), carbon-linked C8-aryl-dG adducts prefer to adopt an *anti* conformation paired opposite C, despite the strong *syn* preference of the free nucleosides. Forcing the C8-aryl-dG adduct into the *anti* conformation strongly decreases duplex stability in the order of increasing C8-aryl ring size. Thus, the smallest FurG lesion is the most flexible and exhibits greater accessibility to the *syn* conformation when paired opposite C. (ii) When the modified *Nar*I(12) sequences were hybridized with the truncated 10-mer sequence (–2), i.e. mimicking a SMI, none of the C-linked C8-dG adducts stabilized the truncated duplex compared to the unmodified control, suggesting the inability of these lesions to induce –2 frameshift mutations. (iii) Primer extension reactions with an adducted *Nar*I(22) template:15mer primer catalyzed by the high-fidelity replicative polymerase *E. coli* pol I Klenow fragment exo^−^ (Kf^−^), indicated that increased C8-aryl ring size inhibits error-prone synthesis by Kf^−^. Thus, the greatest levels of A and G misincorporation by Kf^−^ occurred when the smallest adduct FurG was present in the template. This observation is consistent with its greater conformational flexibility (interchange between *syn* and *anti* structures) compared to the other C-linked C8-aryl-dG adducts with larger C8-aryl substituents. In the *syn* conformation the Hoogsteen H-bonding face of G can base pair with G and A, which provides a rational for the ability of C8-aryl-dG adducts to induce G → T and G → C transversion mutations. (iv) When DNA synthesis was performed by Dpo4, there was a strong tendency for template slippage to occur, blocking full-length extension by the polymerase. For FurG and PhG in the template, we propose that the template:primer complex realigns following 2-base slippage to create a C:C mismatch downstream of the adduct site. Overall, our results suggest that C-linked C8-aryl-dG adducts promote G → T and G → C transversion mutations due to their syn conformational preference and effectively stall DNA replication to permit slippage of the primer at reiterated sequences to afford mutations in the vicinity of the adduct (semi-targeted mutations). These observations provide a chemical basis for the mutagenic potential of C8-aryl-dG adducts.

## SUPPLEMENTARY DATA

Supplementary Data are available at NAR Online.

SUPPLEMENTARY DATA
