# Supplementary material for: Structural and biochemical impact of C8-aryl-guanine adducts within the NarI recognition DNA sequence: influence of aryl ring size on targeted and semi-targeted mutagenicity
Source: Nucleic Acids Res. 2014 Oct 31;42(21):13405–21. doi: 10.1093/nar/gku1093 (PMC4245952; doi:10.1093/nar/gku1093)
Supplement: SUPPLEMENTARY DATA [file supp_42_21_13405__index.html]

Structural and biochemical impact of C8-aryl-guanine adducts within the NarI recognition DNA sequence: influence of aryl ring size on targeted and semi-targeted mutagenicity — Structural and biochemical impact of C8-aryl-guanine adducts within the NarI recognition DNA sequence: influence of aryl ring size on targeted and semi-targeted mutagenicity — SUPPLEMENTARY DATA 

# Structural and biochemical impact of C8-aryl-guanine adducts within the *Nar*I recognition DNA sequence: influence of aryl ring size on targeted and semi-targeted mutagenicity

## SUPPLEMENTARY DATA

**Files in this Data Supplement:**

- SUPPLEMENTARY DATA
