# Supplementary material for: Structural and biochemical impact of C8-aryl-guanine adducts within the NarI recognition DNA sequence: influence of aryl ring size on targeted and semi-targeted mutagenicity
Source: Nucleic Acids Res. 2014 Oct 31;42(21):13405–21. doi: 10.1093/nar/gku1093 (PMC4245952; doi:10.1093/nar/gku1093)
Supplement: SUPPLEMENTARY DATA [file supp_gku1093_nar-02740-h-2014-File002.pdf]

Supplementary Data for:

Structural and Biochemical Impact of C8-Aryl-Guanine Adducts within the *Nar*I Recognition DNA Sequence: Influence of Aryl Ring Size on Targeted and Semitargeted Mutagenicity

Michael Sproviero, Anne M. R. Verwey, Katherine M. Rankin, Aaron A. Witham, Dmitriy V. Soldatov, Richard A. Manderville, Mostafa I. Fekry, Shana J. Sturla,* Purshotam Sharma, and Stacey D. Wetmore**

Table of Contents:

1. **Figures S1,S2.** Selected bond lengths and dihedral angles for the minima and transition states of the PhG and QG adduct……………………………………………………………...S2
2. **Table S1.** χ and θ dihedral angles for the starting structures used for MD simulations..S4
3. **Figures S3-S9.** Initial (energy minimized) structures used for MD simulations…...…...S6
4. **Figures S10-S17.** Radar plots for the percent distribution of the χ and θ dihedral angles throughout the 40 ns trajectories…………………………………………………………..S13
5. **Table S2**. Occupancies for the hydrogen bonds between the adduct and the opposing base for the studied adduct conformations over the duration of the MD simulations…...…..S21
6. **Figures S18-S29.** Representative structures from MD simulations……………....…...S23
7. **Table S3.** Free energy analysis of MD simulations of different adduct conformations.S35
8. **Figures S30, S31.** Backbone RMSD for MD simulations on the studied adducts in different conformations in the *Nar*I duplex…………………………………………….S37
9. **S32.** Mol2 file for the QG nucleoside adduct…………...……………….…………….S39
10. **S34.** Detailed results from MD simulations…………………………………………S40
11. **Figure S34.** Emission spectra of QG and rationale…..………………………………...S49
12. **Figure S35.** Fluorescence emission spectra of C8-aryl-G modified *Nar*I(12)…………S50
13. **S35.** Crystal structure analysis of QG monohydrate……….……………...…………S51

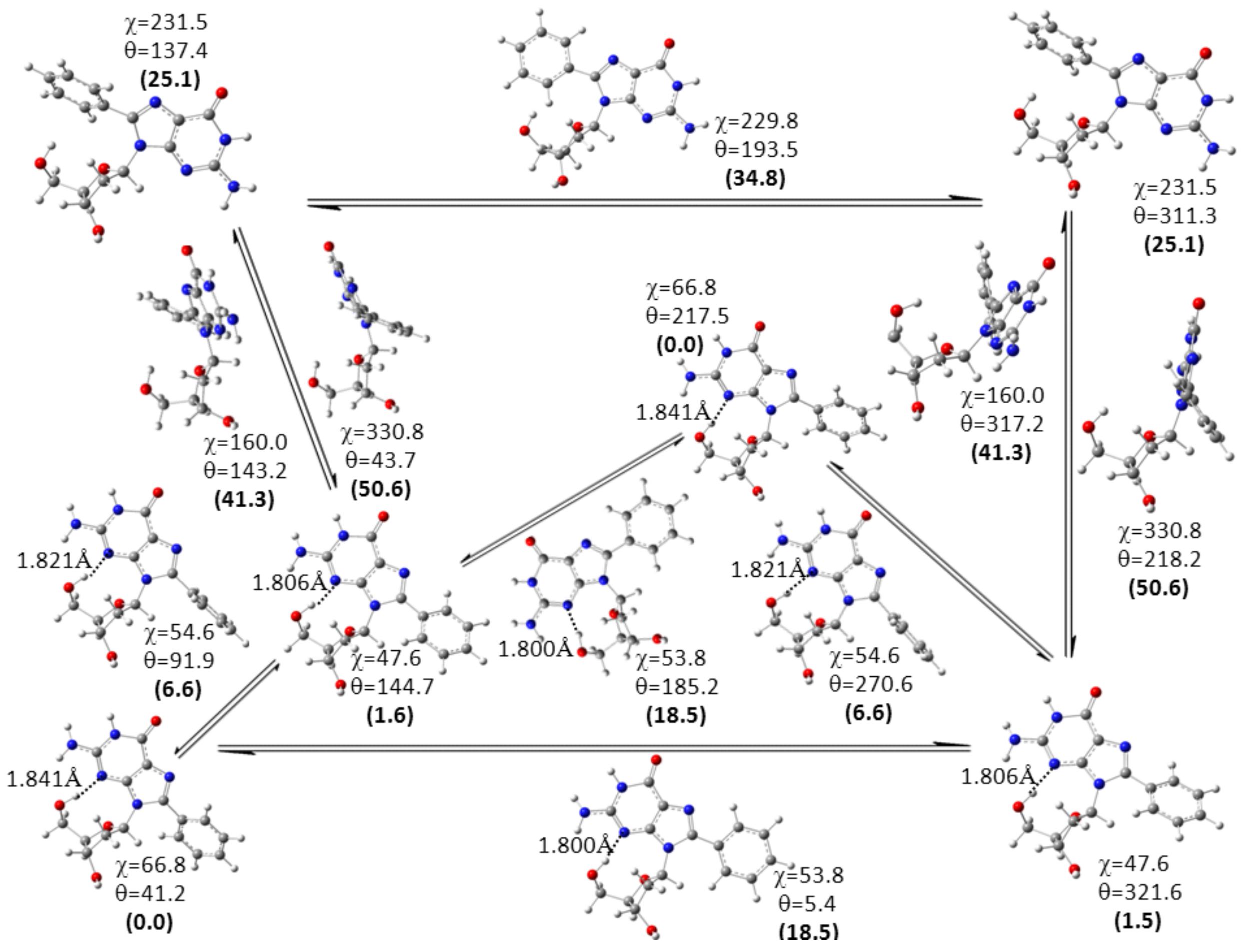

Figure S1. Selected B3LYP/6-31G(d) bond lengths (Å) and dihedral angles (χ and θ, deg.) for the minima and transition states of the PhG adduct. (ZPE corrected relative energies from B3LYP/6-311+G(2df,p) single-point calculations provided in parenthesis, kJ mol^{-1}).

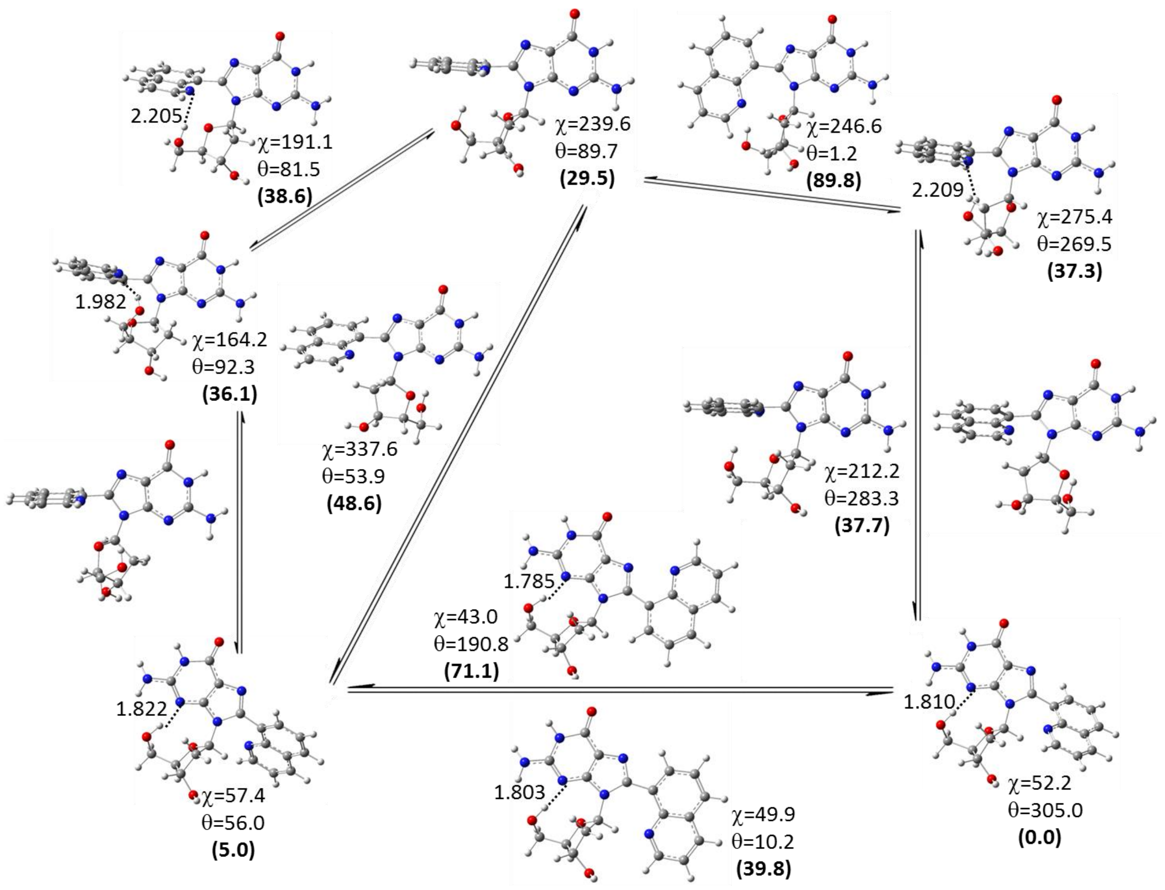

Figure S2. Selected B3LYP/6-31G(d) bond lengths (Å) and dihedral angles (χ and θ, deg.) for the minima and transition states of the QG adduct. (ZPE corrected relative energies from B3LYP/6-311+G(2df,p) single-point calculations provided in parenthesis, kJ mol^{-1}).

Table S1. χ and θ dihedral angles for the starting structures used for MD simulations

adduct	opposite base	adduct conformation	before minimization χ (deg.)	θ (deg.)	after minimization χ (deg.)	θ (deg.)
FurG	*anti*-C	*anti* (θ~0°)	265.8	340.2	236.4	2.1
		anti (θ~180°)	265.8	161.2	237.2	179.0
		syn (θ~0°)	100.1	340.2	44.1	358.4
		syn (θ~180°)	100.1	180.0	43.8	176.9
PhG	*anti*-C	*anti*	269.8	20.2	270.8	22.5
		syn	93.2	41.0	70.1	9.3
CNPhG	*anti*-C	*anti*	269.8	20.2	272.3	24.2
		syn	93.2	357.5	77.5	14.2
QG	*anti*-C	*anti* (θ~0°)[a]	206.8	356.6	217.4	19.7
		anti (θ~180°)	265.8	162.3	225.9	195.0
		syn (θ~0°)	100.1	12.4	67.9	17.1
		syn (θ~180°)	100.1	198.7	79.1	194.4
FurG	*syn*-G	*anti* (θ~0°)	265.8	340.2	240.1	359.9
		anti (θ~180°)	265.8	174.1	239.6	181.5
	anti-G	*syn* (θ~0°)	88.3	340.2	69.1	0.3
		syn (θ~180°)	88.3	13.7	79.0	186.1
PhG	*syn*-G	*anti*	272.7	345.7	75.1	13.7
	anti-G	*syn*	82.4	345.7	237.3	359.5
CNPhG	*syn*-G	*anti*	272.7	345.7	245.4	349.4
	anti-G	*syn*	82.4	345.7	88.8	350.4
QG	*syn*-G	*anti* (θ~0°)[a]	279.0	0.5	271.8	345.4
		anti (θ~180°)	265.8	153.4	252.8	169.6
	anti-G	*syn* (θ~0°)	88.3	22.2	75.4	19.9
		syn (θ~180°)	88.3	180.0	82.5	186.1
FurG	THF	*anti* (θ~0°)	230.8	0.8	242.2	1.0
		anti (θ~180°)	230.8	180.0	244.2	177.7
		syn (θ~0°)	40.0	0.0	40.8	1.0
		syn (θ~180°)	40.0	180.0	39.8	180.0
PhG	THF	*anti*	228.2	5.3	223.6	5.6
		syn	48.8	5.3	41.5	350.3
CNPhG	THF	*anti*	226.6	8.1	232.9	2.9
		syn	38.0	8.2	42.3	355.3
QG	THF	*anti* (θ~0°)	220.1	0.0	211.5	23.0
		anti (θ~180°)	220.1	184.7	223.1	187.5
		syn (θ~0°)	31.0	0.1	31.6	3.0
		syn (θ~180°)	31.0	180.0	29.2	180.6
FurG	-2	*anti* (θ~0°)	252.0	0.0	236.0	0.0
		anti (θ~180°)	252.0	180.0	231.9	177.3
		syn (θ~0°)	68.5	1.5	56.9	2.4
		syn (θ~180°)	68.5	180.0	70.9	179.8
PhG	-2	*anti*	252.0	82.4	243.0	350.2

		syn	75.4	41.0	77.9	10.0
CNPhG	-2	*anti*	252.0	82.4	236.5	348.6
		syn	75.4	41.0	49.9	19.6
QG	-2	*anti* (θ~0°)	219.5	17.1	213.7	24.4
		anti (θ~180°)	219.5	206.1	210.8	200.1
		syn (θ~0°)	68.5	17.2	56.3	198.8
		syn (θ~180°)	68.5	180.0	57.6	16.0

[a]Upon introduction of the C8-quinoyl moiety to DNA to incorporate *anti*-QG (θ ~ 0°) against C or G using Gaussview, large steric clashes occurred, which yielded highly distorted structures after energy minimization. For this reason, simulations for these conformations were carried out by rebuilding the initial structure by changing θ ~ 180° to θ ~ 0° in the structure from the last frame of the simulation of the respective *anti*-QG (θ ~ 180°) conformation, while retaining the χ dihedral angle.

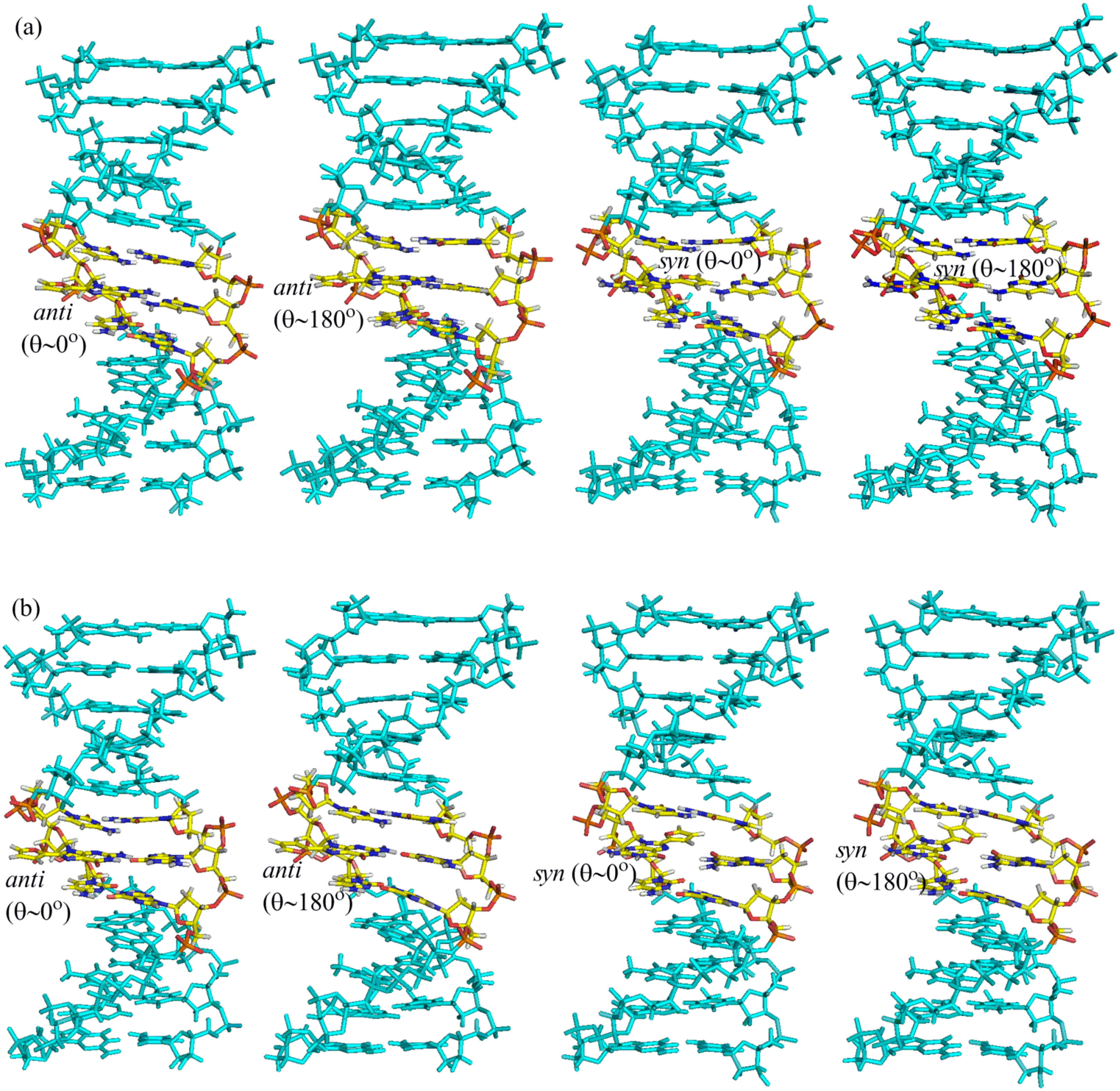

Figure S3: Initial (energy minimized) structures used for MD simulations of the FurG adduct in the *Nar*I helix paired against (a) the complementary C and (b) a G mismatch.

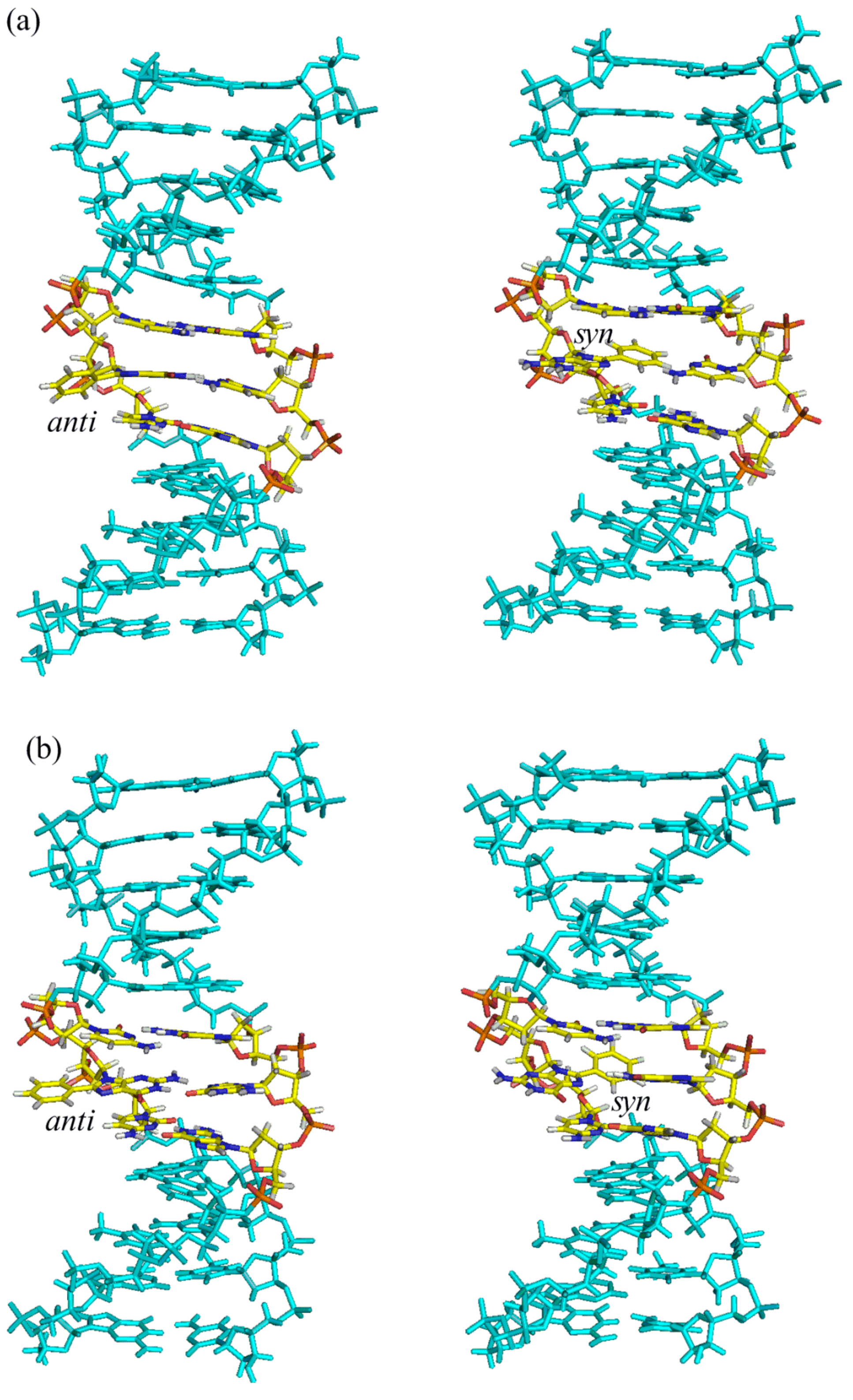

Figure S4: Initial (energy minimized) structures used for MD simulations of the PhG adduct in the *Nar*I helix paired against (a) the complementary C and (b) a G mismatch.

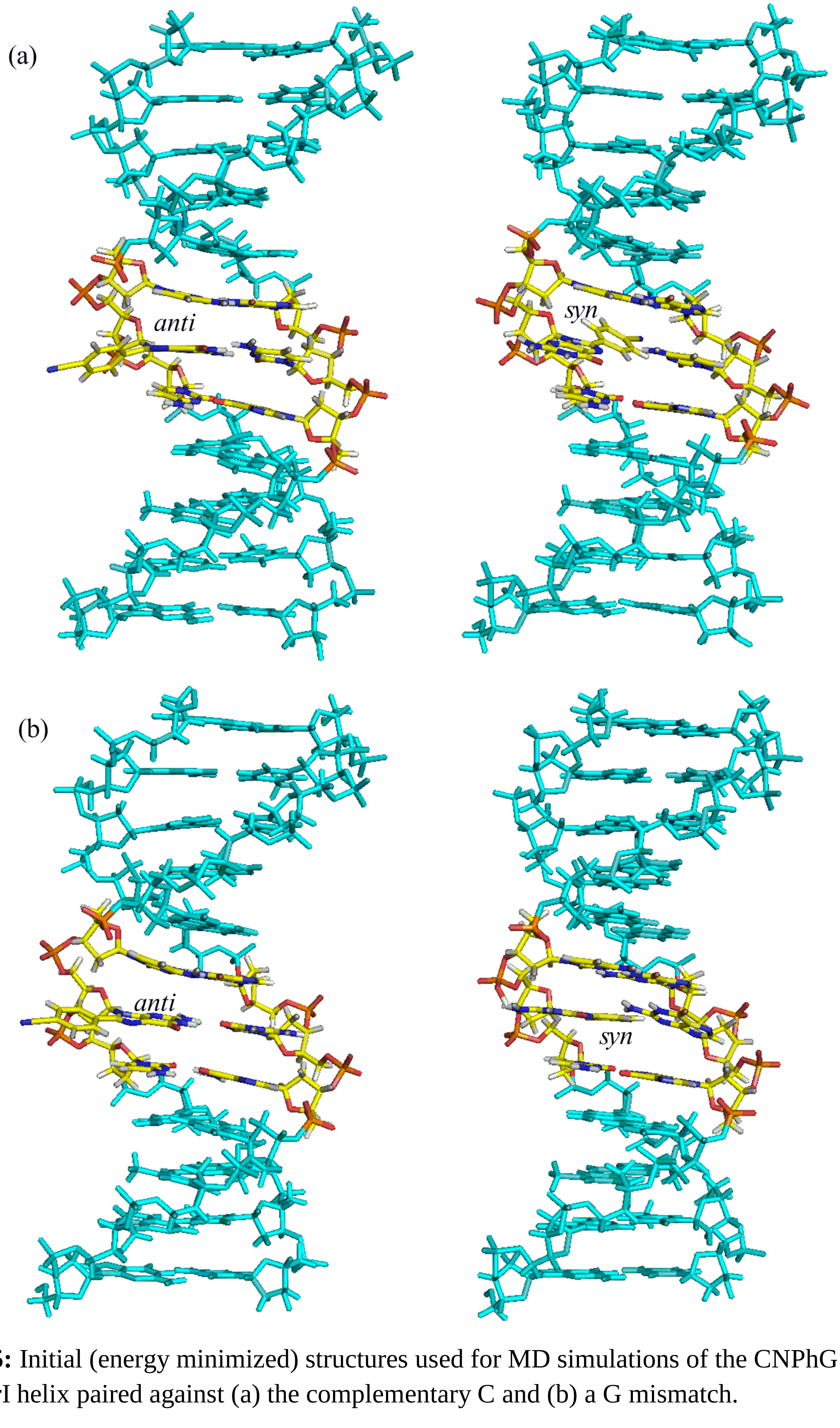

Figure S5: Initial (energy minimized) structures used for MD simulations of the CNPhG adduct in the *Nar*I helix paired against (a) the complementary C and (b) a G mismatch.

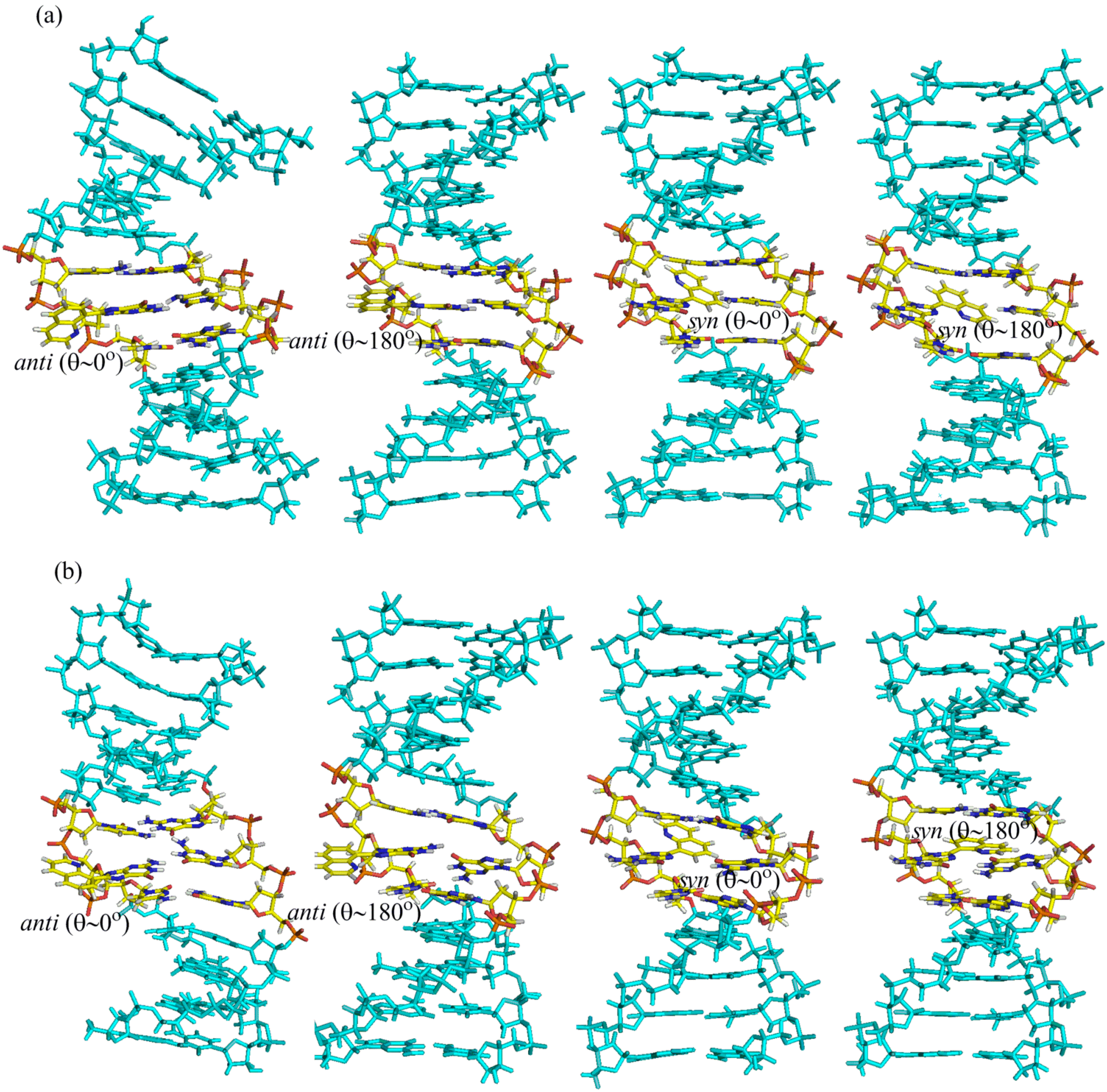

Figure S6: Initial (energy minimized) structures used for MD simulations of the QG adduct in the *Nar*I helix paired against (a) the complementary C and (b) a G mismatch.

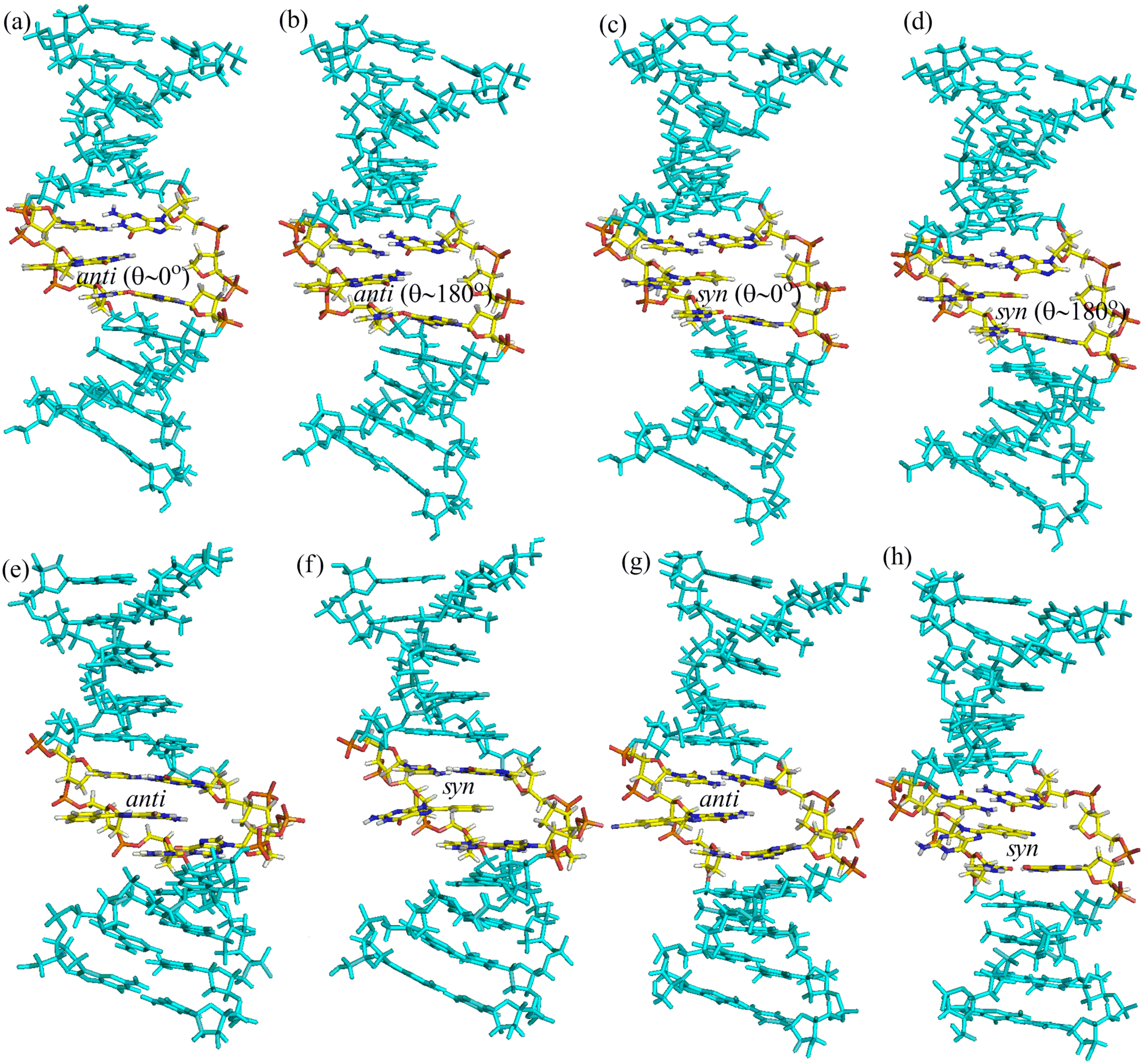

Figure S7: Initial (energy minimized) structures used for MD simulations of the (a-d) FurG, (e and f) PhG and (g and h) CNPhG adducts in the *Nar*I helix paired against THF.

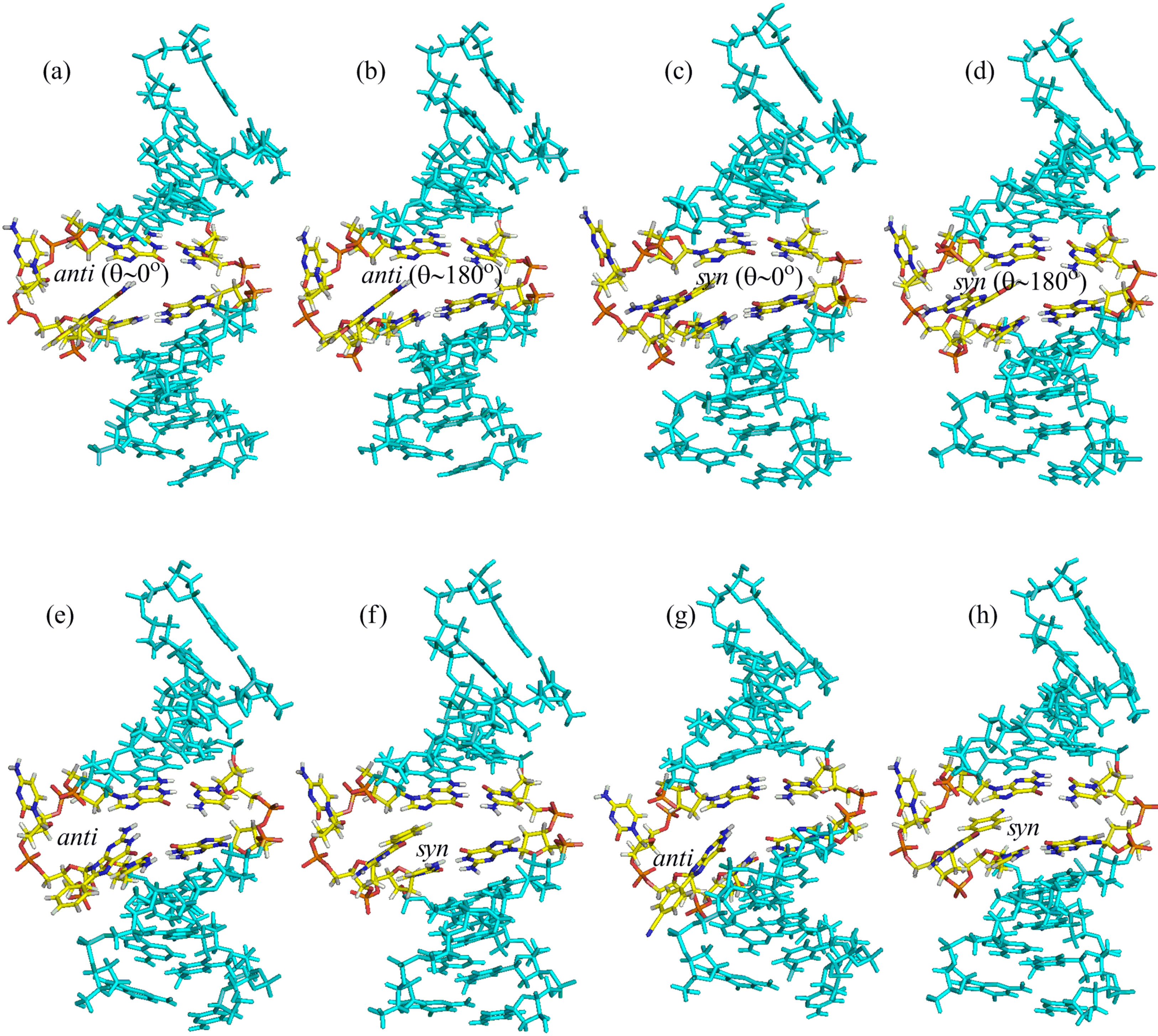

Figure S8: Initial (energy minimized) structures used for MD simulations of the (a-d) FurG, (e and f) PhG and (g and h) CNPhG adducts in the *Nar*I helix paired against -2 base deletion.

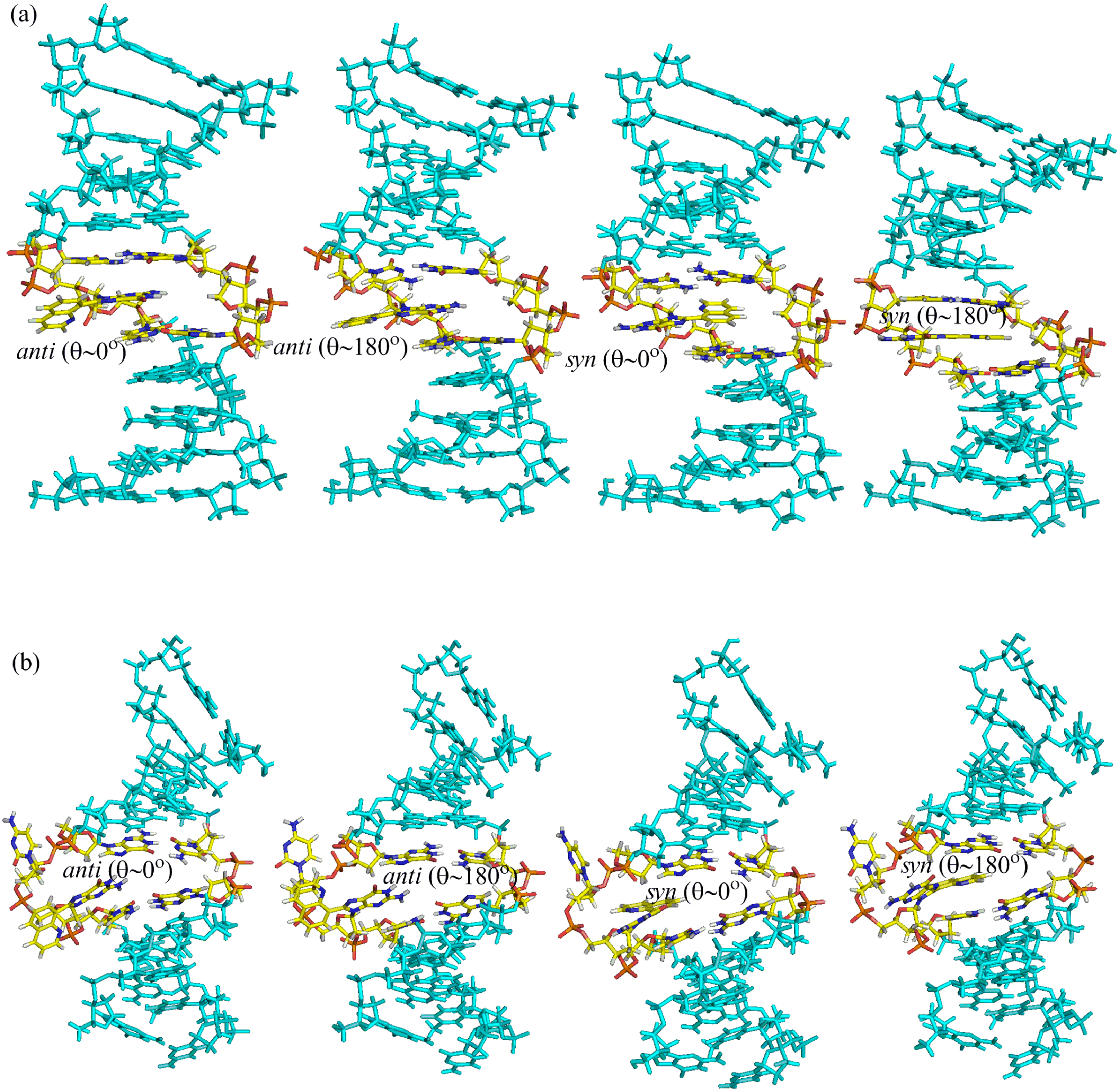

Figure S9: Initial (energy minimized) structures used for MD simulations of the QG adduct in the *Nar*I helix paired against (a) THF and (b) -2 base deletion.

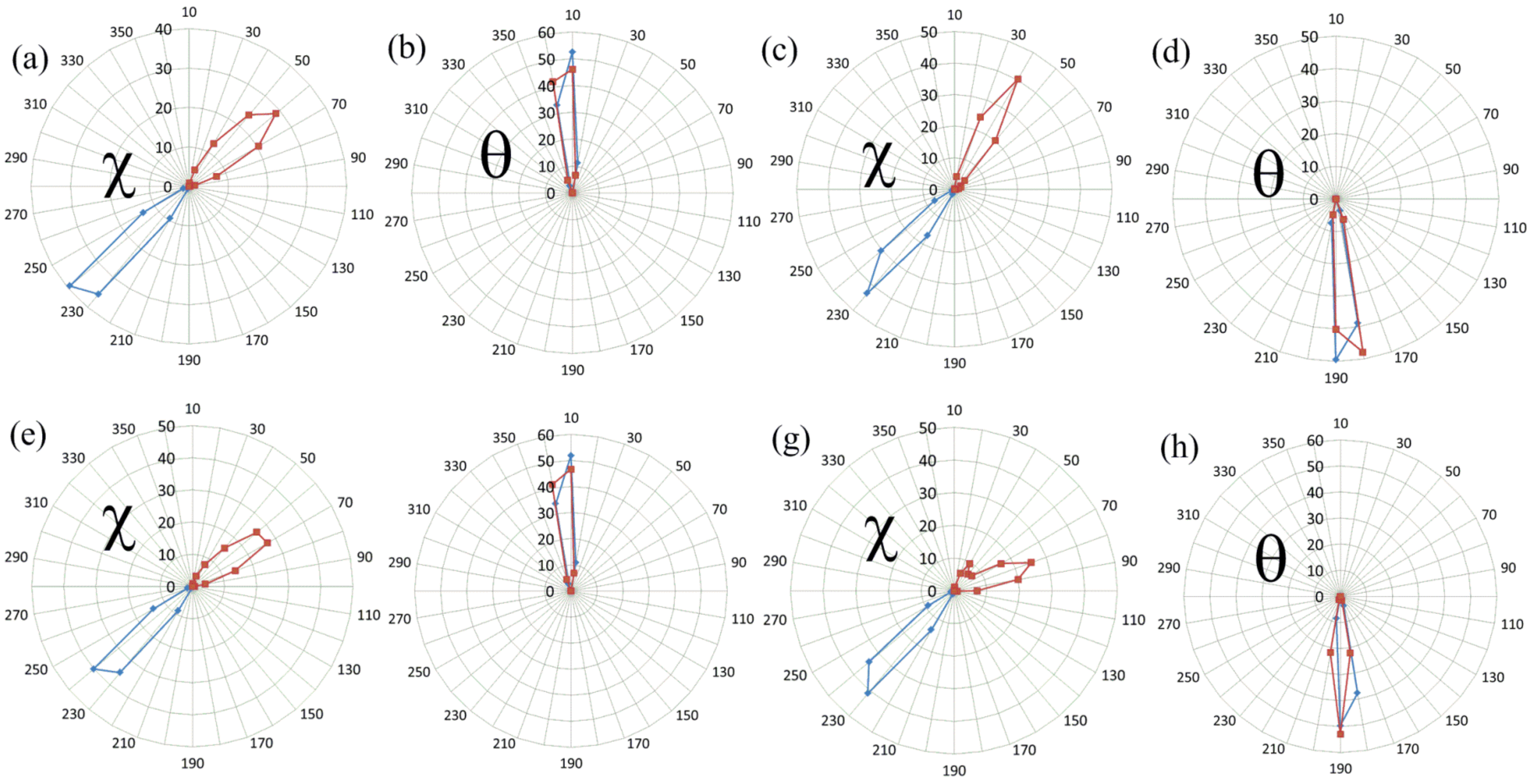

Figure S10: Radar plots for the percent distribution of the χ (deg.) and θ (deg.) dihedral angles throughout the 40 ns trajectories for the FurG adduct paired in the *syn* (red) and *anti* (blue) conformations against complementary C (a-d) and G mismatch (e-h). Figures a, b, e and f correspond to simulations with θ ~ 0°, whereas figures c, d, g and h correspond to simulations with θ ~ 180°.

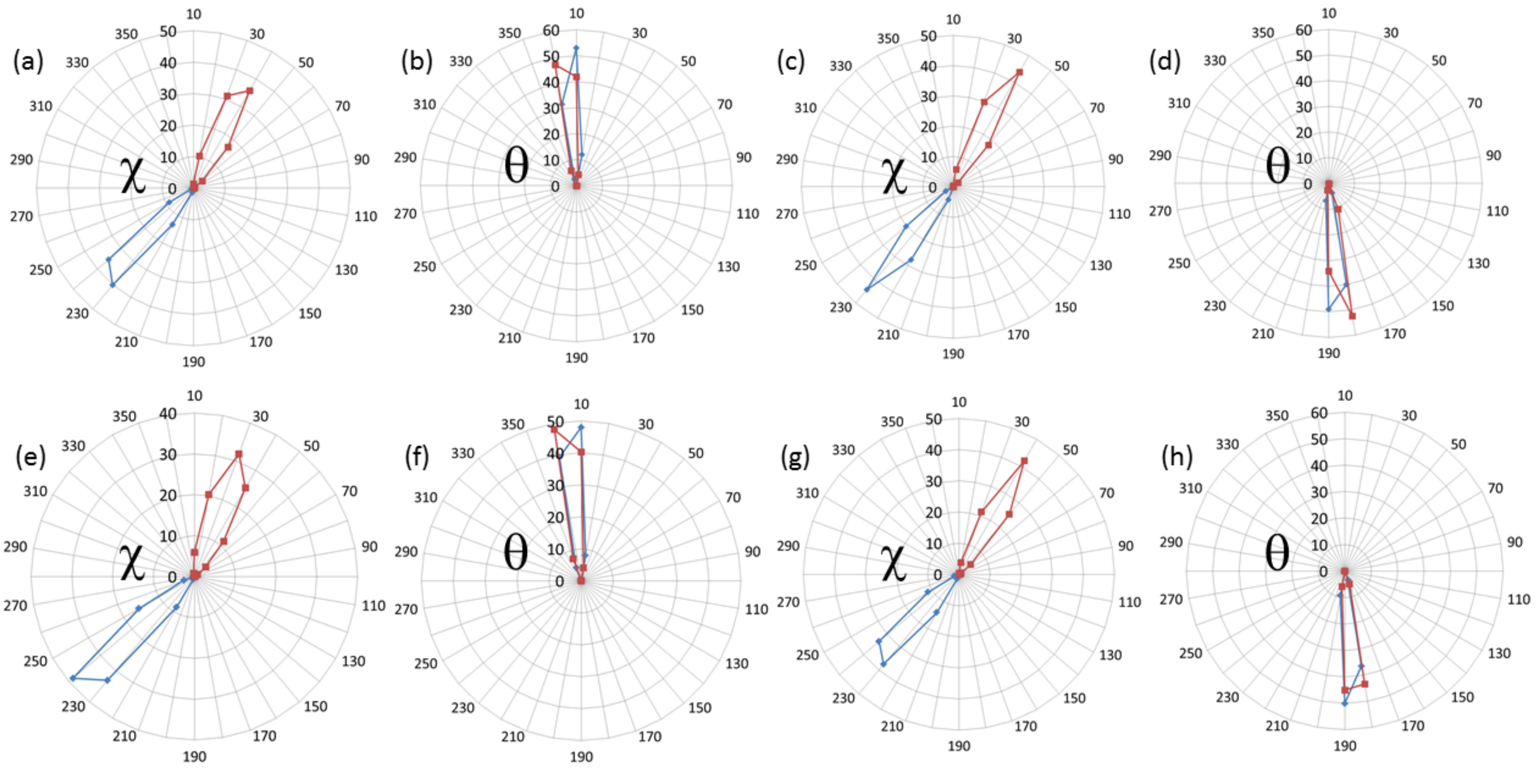

Figure S11: Radar plots for the percent distribution of the χ (deg.) and θ (deg.) dihedral angles throughout the 40 ns trajectories for the FurG adduct paired in the *syn* (red) and *anti* (blue) conformations against complementary THF (a-d) and -2 base deletion (e-h). Figures a, b, e and f correspond to simulations with θ ~ 0°, whereas figures c, d, g and h correspond to simulations with θ ~ 180°.

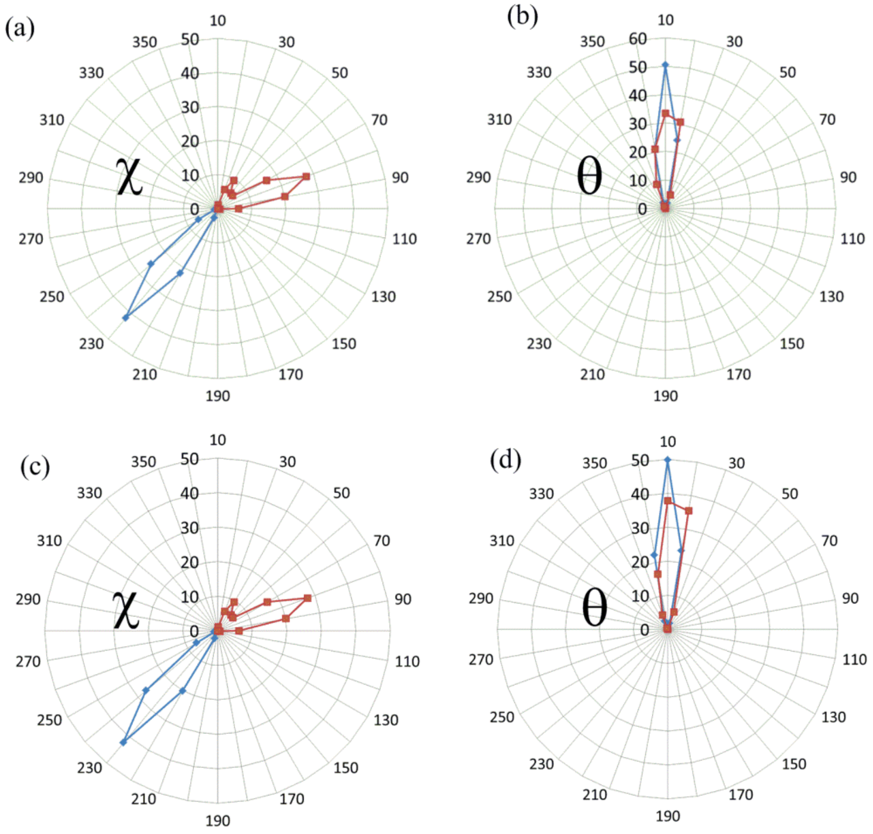

Figure S12: Radar plots for the percent distribution of the χ (deg.) and θ (deg.) dihedral angles throughout the 40 ns trajectories for the PhG adduct paired in its *syn* (red) and *anti* (blue) conformations against complementary C (a and b) and G mismatch (c and d).

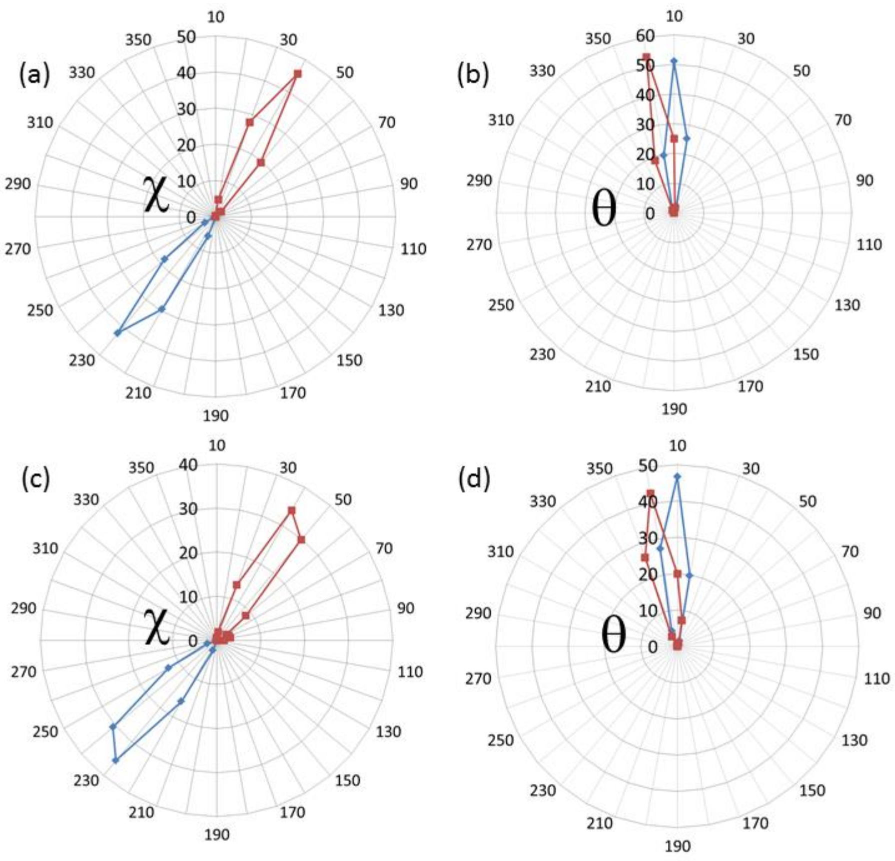

Figure S13: Radar plots for the percent distribution of the χ (deg.) and θ (deg.) dihedral angles throughout the 40 ns trajectories for the PhG adduct paired in its *syn* (red) and *anti* (blue) conformations against THF (a and b) and -2 base deletion (c and d).

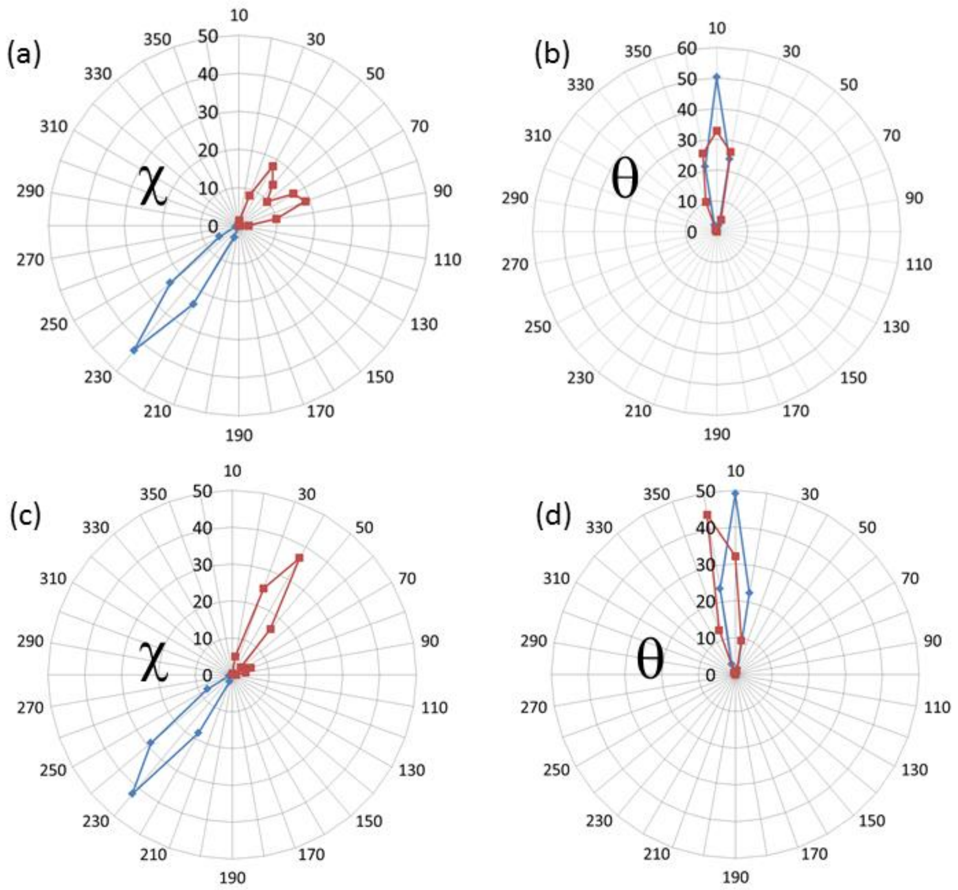

Figure S14: Radar plots for the percent distribution of the χ (deg.) and θ (deg.) dihedral angles throughout the 40 ns trajectories for the CNPhG adduct paired in its *syn* (red) and *anti* (blue) conformations against complementary C (a and b) and G mismatch (c and d).

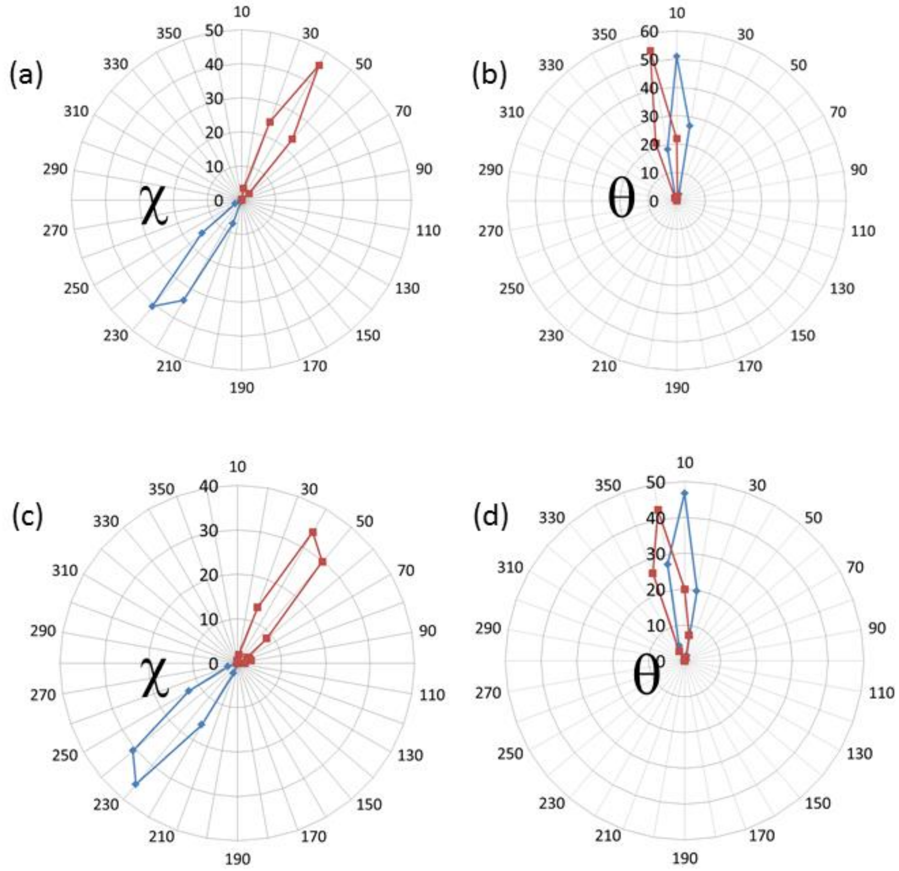

Figure S15: Radar plots for the percent distribution of the χ (deg.) and θ (deg.) dihedral angles throughout the 40 ns trajectories for the CNPhG adduct paired in its *syn* (red) and *anti* (blue) conformations against THF (a and b) and -2 base deletion (c and d).

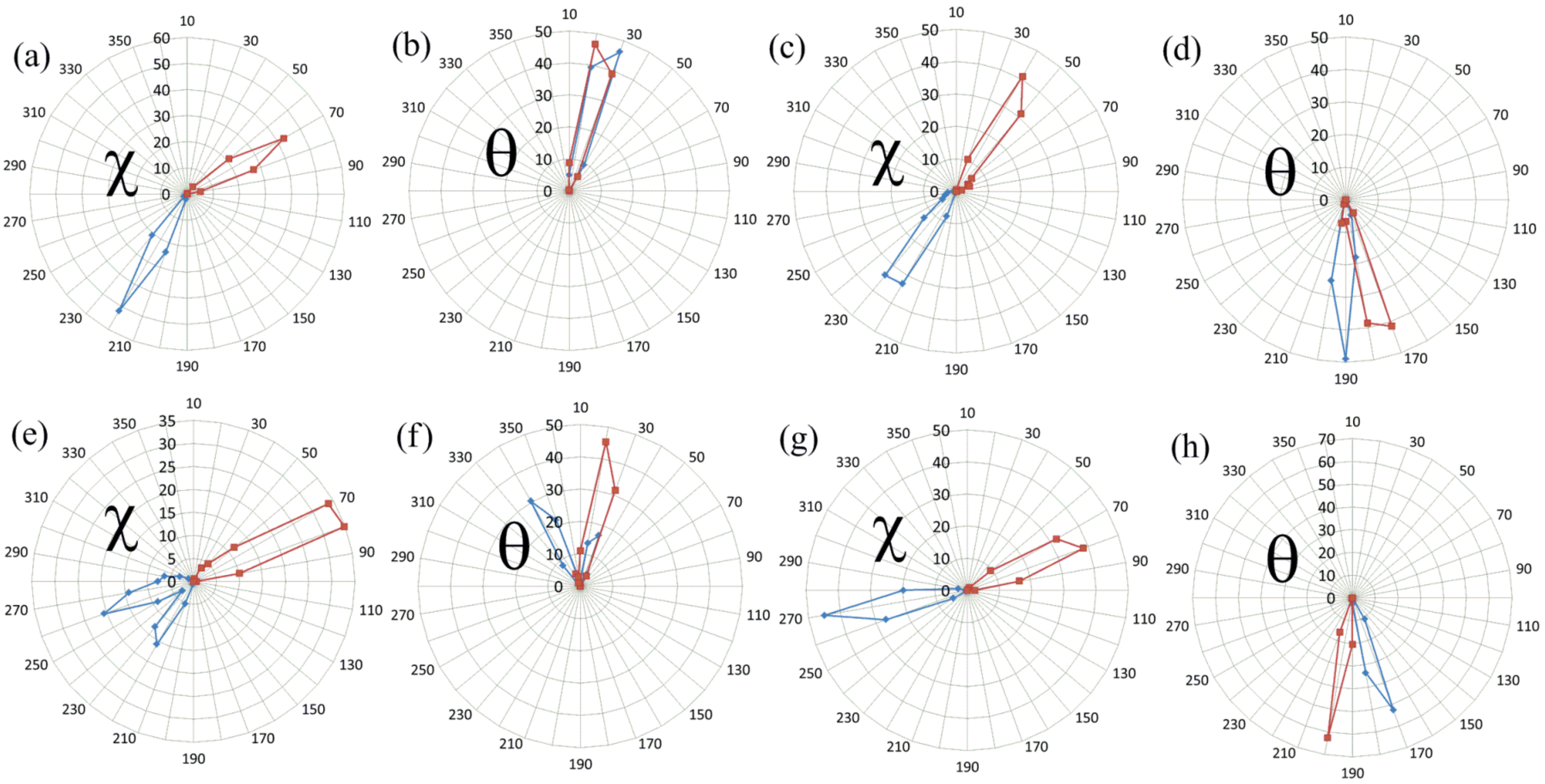

Figure S16: Radar plots for the percent distribution of the χ (deg.) and θ (deg.) dihedral angles throughout the 40 ns trajectories for the QG adduct paired in its *syn* (red) and *anti* (blue) conformations against complementary C (a-d) and G mismatch (e-h). Figures a, b, e and f correspond to simulations with θ ~ 0°, whereas figures c, d, g and h correspond to simulations with θ ~ 180°.

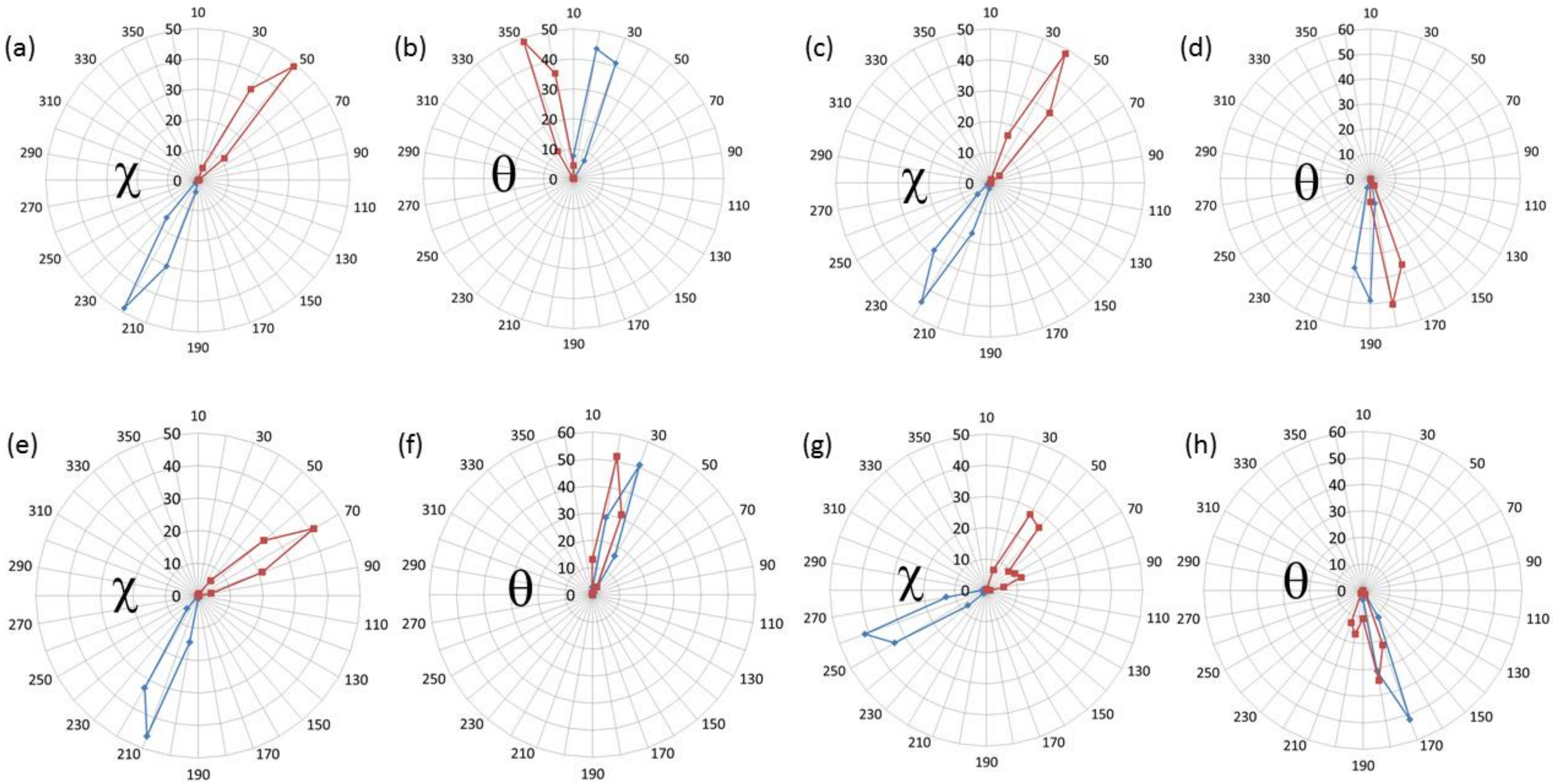

Figure S17: Radar plots for the percent distribution of the χ (deg.) and θ (deg.) dihedral angles throughout the 40 ns trajectories for the QG adduct paired in its *syn* (red) and *anti* (blue) conformations against THF (a-d) and -2 base deletion (e-h). Figures a, b, e and f correspond to simulations with θ ~ 0°, whereas figures c, d, g and h correspond to simulations with θ ~ 180°.

Table S2. Occupancies for the hydrogen bonds between the adduct and the opposing base for the studied adduct conformation(s) over the duration of the MD simulations[a]

adduct conformation	opposite base conformation	bond	% occupancy
anti–FurG (θ~0°)	*anti*-C	N2-H(FurG)…O2(C)	99.9
		N1-H(FurG)…N3(C)	99.9
		N4-H(C)…O6(FurG)	98.6
anti–FurG (θ~180°)	*anti*-C	N2-H(FurG)…O2(C)	99.9
		N1-H(FurG)…N3(C)	99.9
		N4-H(C)…O6(FurG)	98.7
syn–FurG (θ~0°)	*anti*-C	N4-H1(C)…N7(FurG)	79.8
		N4-H2(C)…O6(FurG)	70.1
syn–FurG (θ~180°)	*anti*-C	N4-H2(C)…O^{ring}(FurG)	32.3
anti–PhG	*anti*-C	N2-H(PhG)…O2(C)	99.9
		N1-H(PhG)…N3(C)	99.9
		N4-H(C)…O6(PhG)	98.8
syn–PhG	*anti*-C	N4-H1(C)…N7(PhG)	63.5
		N4-H2(C)…O6(PhG)	59.2
anti–CNPhG	*anti*-C	N2-H(CNPhG)…O2(C)	99.9
		N1-H(CNPhG)…N3(C)	99.9
		N4-H(C)…O6(CNPhG)	98.7
syn–CNPhG	*anti*-C	N4-H1(C)…N7(CNPhG)	64.5
		N4-H2(C)…O6(CNPhG)	44.2
anti–QG (θ~0°)	*anti*-C	N2-H(QG)…O2(C)	99.9
		N1-H(QG)…N3(C)	99.9
		N4-H(C)…O6(QG)	98.4
anti–QG (θ~180°)	*anti*-C	N2-H(QG)…O2(C)	99.9
		N1-H(QG)…N3(C)	99.9
		N4-H(C)…O6(QG)	98.7
syn–QG (θ~0°)	*anti*-C	N4-H1(C)…N7(QG)	77.0
		N4-H2(C)…N7(QG)	52.5
anti–FurG (θ~0°)	*syn*-G	N2-H(FurG)…N7(G)	97.5
		N1-H(FurG)…O6(G)	52.6
		N1-H(FurG)…N7(G)	52.3
anti–FurG (θ~180°)	*syn*-G	N2-H(FurG)…N7(G)	96.6
		N1-H(FurG)…O6(G)	45.6
		N1-H(FurG)…N7(G)	61.9
syn–FurG (θ~0°)	*anti*-G	N1-H(G)…O6(FurG)	72.8
		N2-H(G)…O6(FurG)	72.1
		N2-H(G)…N7(FurG)	55.3
syn–FurG (θ~180°)	*anti*-G	N1-H(G)…O6(FurG)	83.3
		N2-H(G)…N7(FurG)	81.2
anti–PhG	*syn*-G	N2-H(PhG)…N7(G)	92.7
		N1-H(PhG)…O6(G)	49.9

		N1-H(PhG)…N7(G)	52.3
syn–PhG	*anti*-G	N1-H(G)…O6(PhG)	26.1
		N2-H(G)…O6(PhG)	79.7
		N2-H(G)…N7(PhG)	49.5
anti–CNPhG	*syn*-G	N2-H(CNPhG)…N7(G)	97.7
		N1-H(CNPhG)…O6(G)	61.2
		N1-H(CNPhG)…N7(G)	45.9
syn–CNPhG	*anti*-G	N2-H(G)…O6(CNPhG)	77.5
		N2-H(G)…N7(CNPhG)	26.8
anti–QG (θ ~ 0°)	*syn*-G	N2–H(QG)…O6(G)	89.8
anti–QG (θ ~ 180°)	*syn*-G	N2–H(QG)…O6(G)	92.6
		N1–H(QG)…O6(G)	55.5
syn–QG (θ ~ 0°)	*anti*–G	N2–H(G)…O6(QG)	72.5
		N2–H(G)…N7(QG)	56.3
		N1–H(G)…O6(QG)	78.5
syn–QG (θ ~ 180°)	*anti*–G	N2–H(G)…O6(QG)	99.3
		N2–H(G)…$N^{Q\text{-}ring}$(QG)	92.8
		N1–H(G)…O6(QG)	30.7

[a]Donor–acceptor (X–Y) distance cutoff of 3.4 Å and X–H–Y angle cutoff of 120° was used for calculating hydrogen bond occupancies. Data provided for H-bonds that persist for greater than 20% of the simulation time.

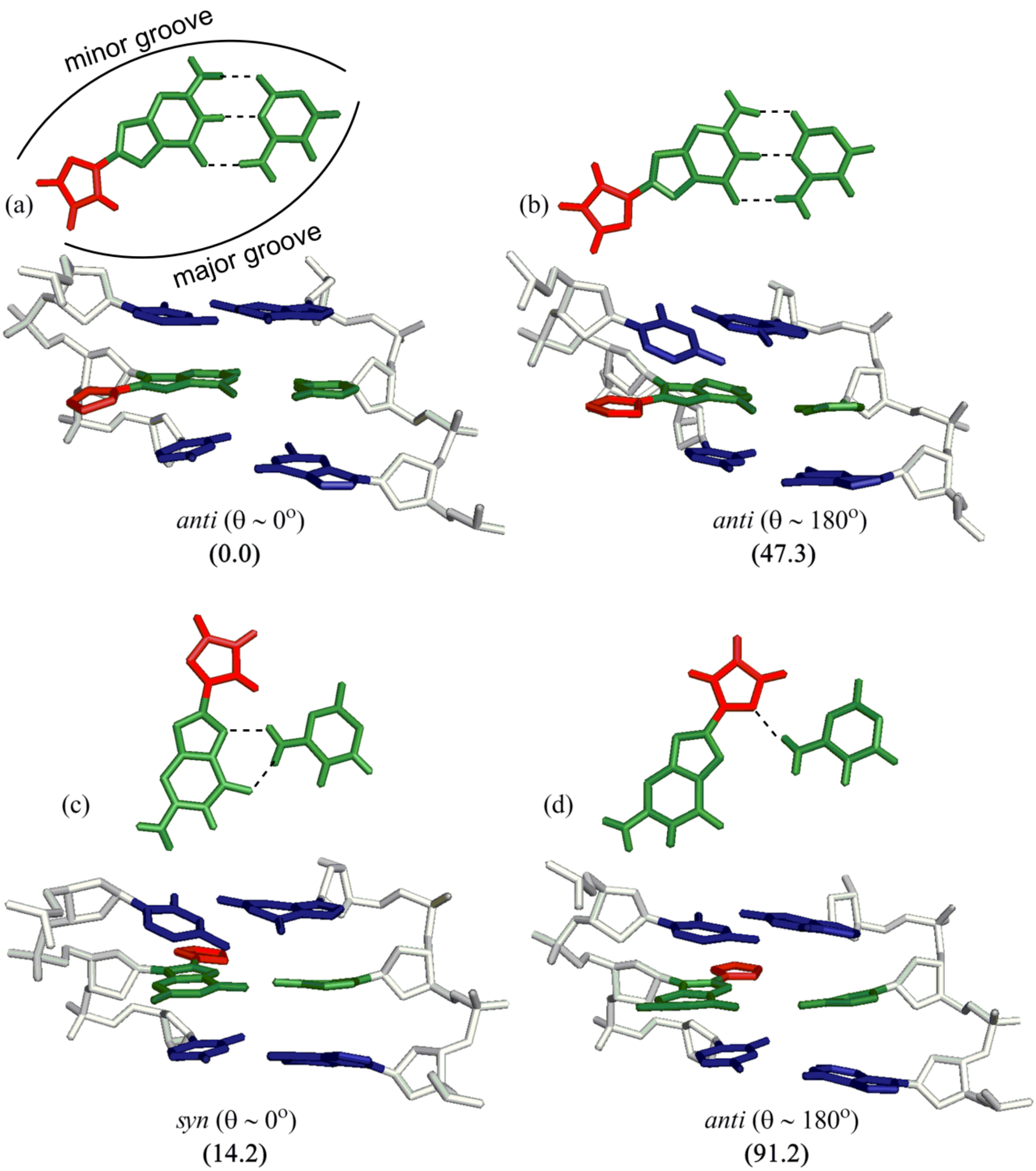

Figure S18. Portions of representative structures showing the orientation of the FurG adduct placed at G^3 position and paired against cytosine in the *Nar*I recognition sequence. The hydrogen bonding pattern of the adduct is also shown explicitly for each of the structures. The corresponding free energy rankings are given in parentheses (bold) against each structure.

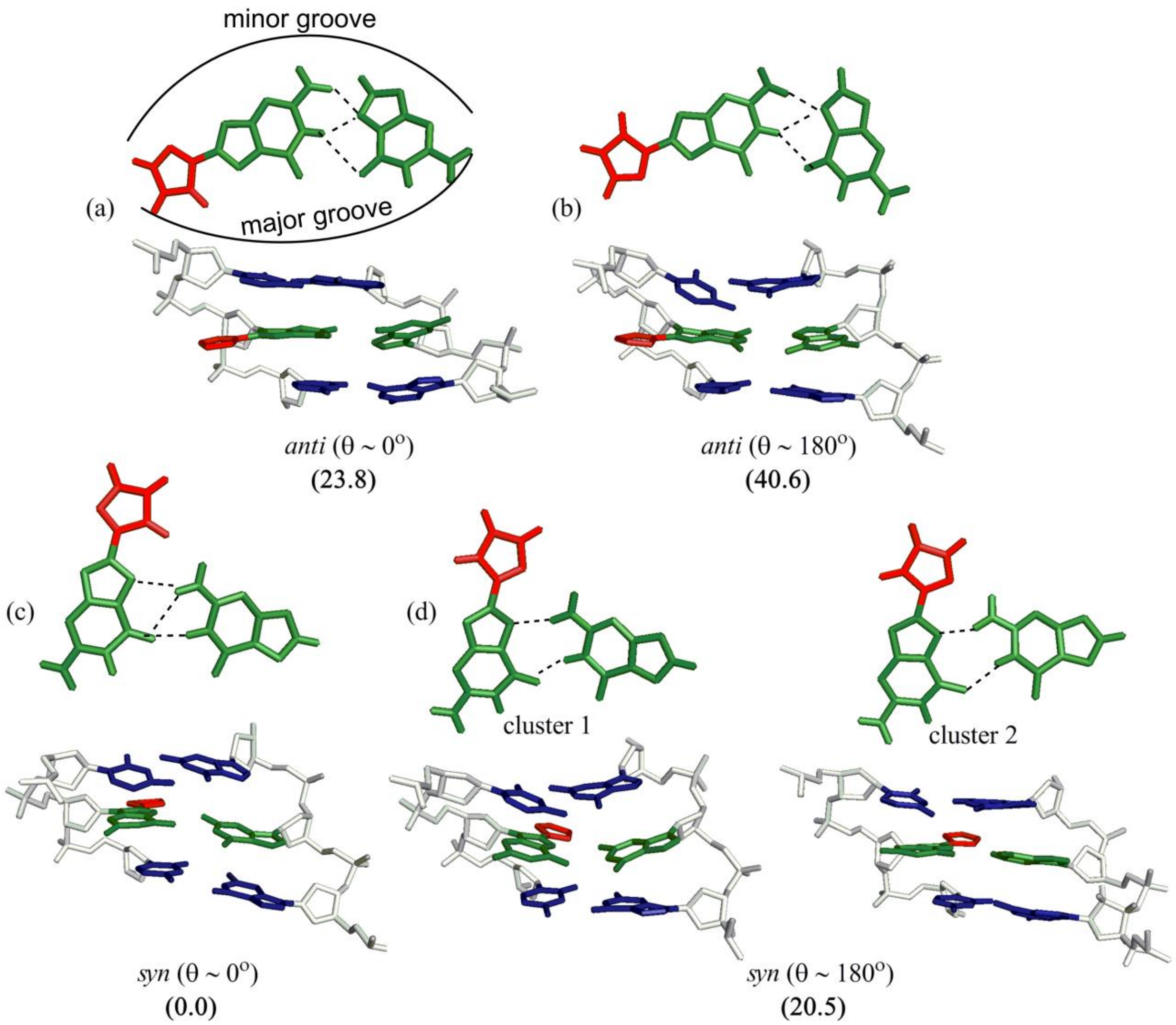

Figure S19. Portions of representative structures showing the orientation of the FurG adduct placed at G^3 position and paired against guanine in the *Nar*I recognition sequence. The hydrogen bonding pattern of the adduct is also shown explicitly for each of the structures. The corresponding free energy rankings are given in parentheses (bold) against each structure.

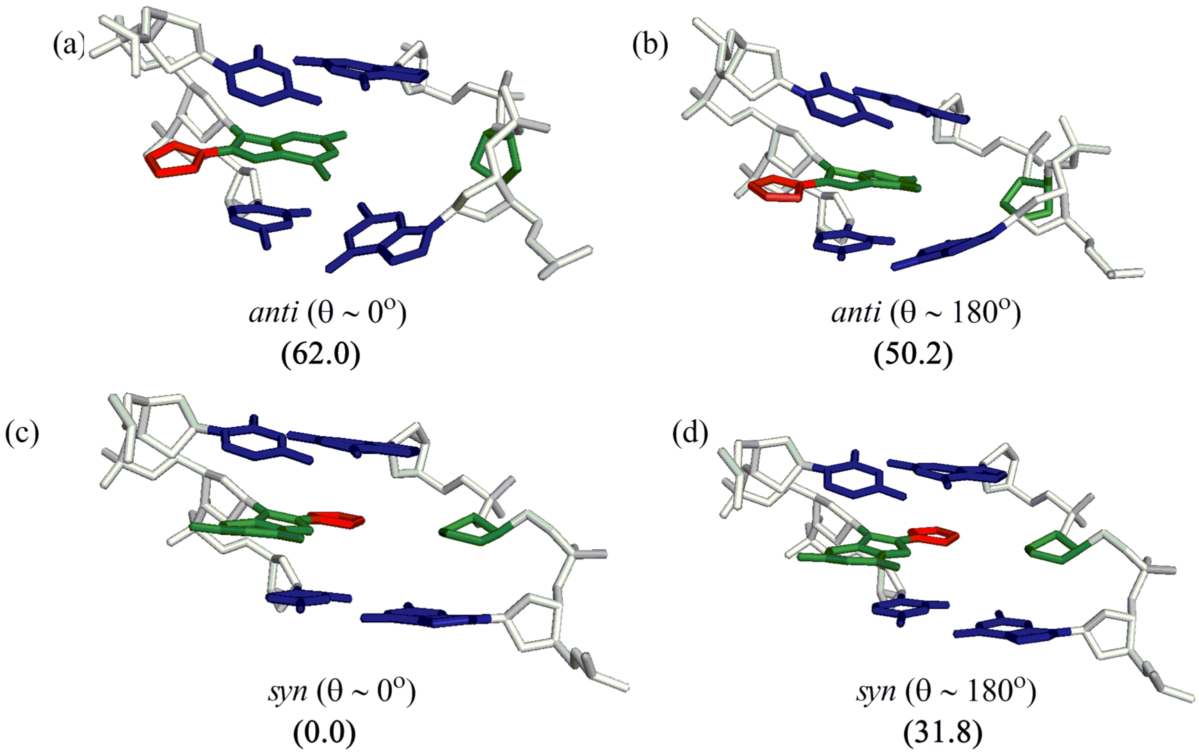

Figure S20. Portions of representative structures showing the orientation of the FurG adduct placed at G^3 position and paired against THF in the *Nar*I recognition sequence. The corresponding free energy rankings are given in parentheses (bold) against each structure.

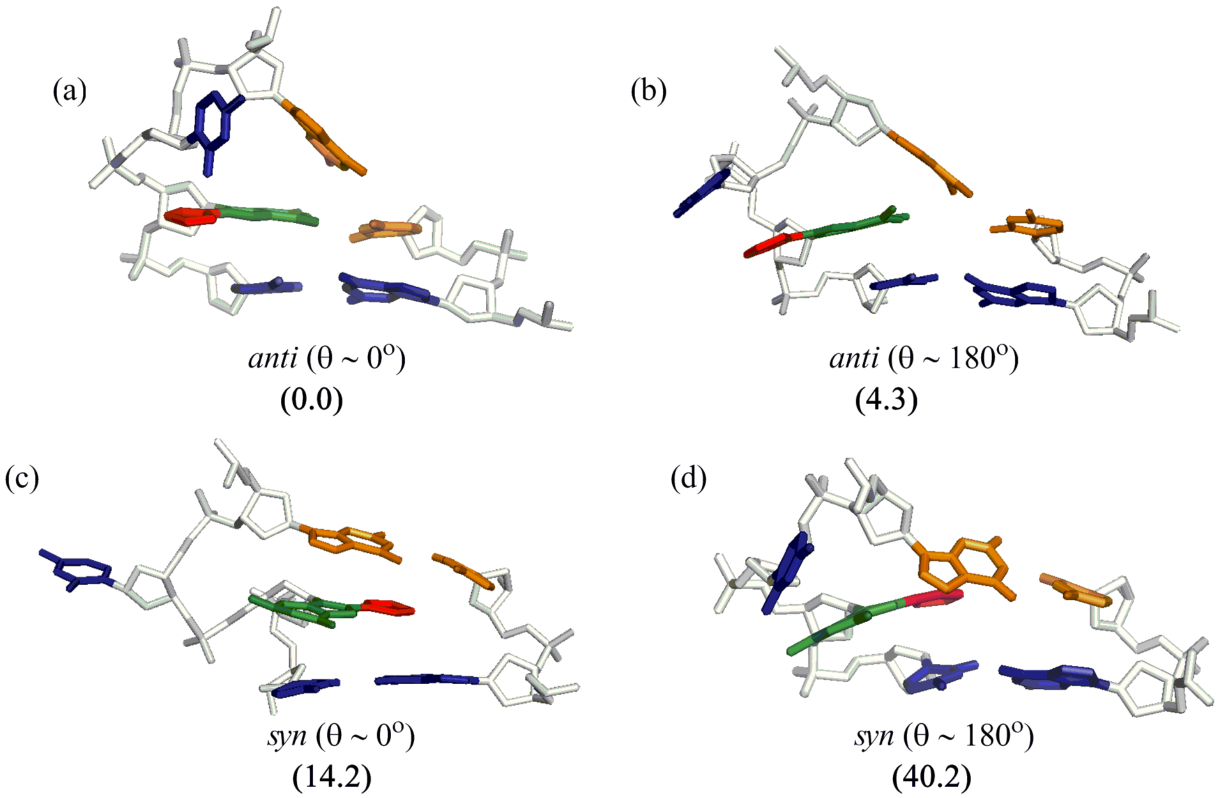

Figure S21. Portions of representative structures showing the orientation of the FurG adduct placed at G^3 position and paired against -2 base deletion in the *Nar*I recognition sequence. The corresponding free energy rankings are given in parentheses (bold) against each structure.

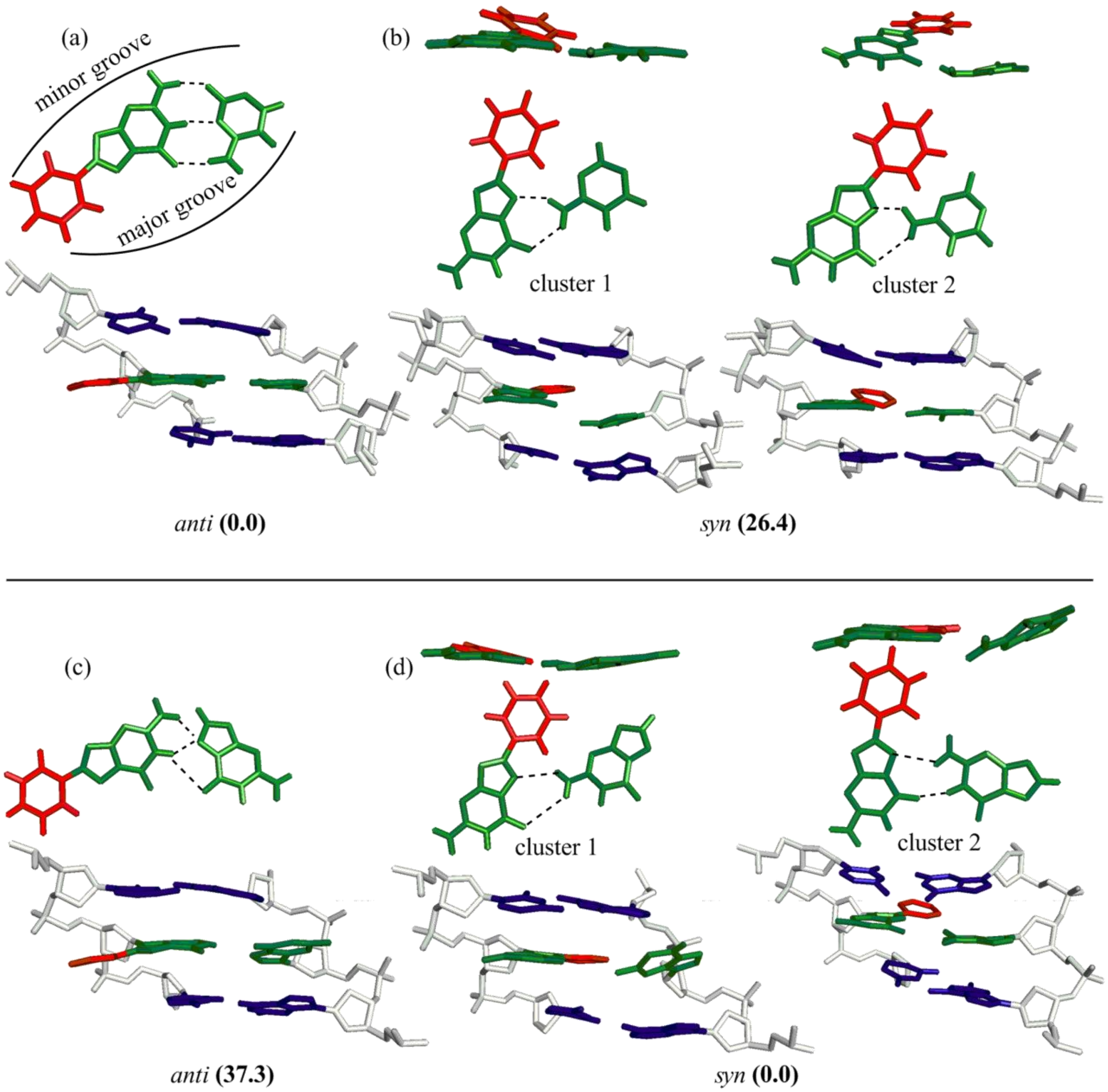

Figure S22. Portions of representative structures showing the orientation of the PhG adduct placed at G^3 position and paired against (a) cytosine and (b) guanine in the *Nar*I recognition sequence. The hydrogen bonding pattern of the adduct is also shown explicitly for each of the structures. The corresponding free energy rankings are given in parentheses (bold) against each structure.

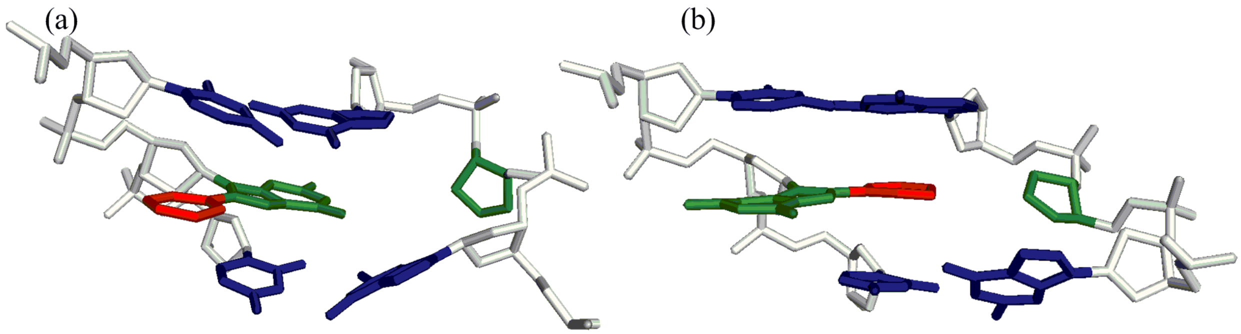

anti **(37.2)** *syn* **(0.0)**

Figure S23. Portions of representative structures showing the orientation of the PhG adduct placed at G^3 position and paired against THF in the *Nar*I recognition sequence. The corresponding free energy rankings are given in parentheses (bold) against each structure.

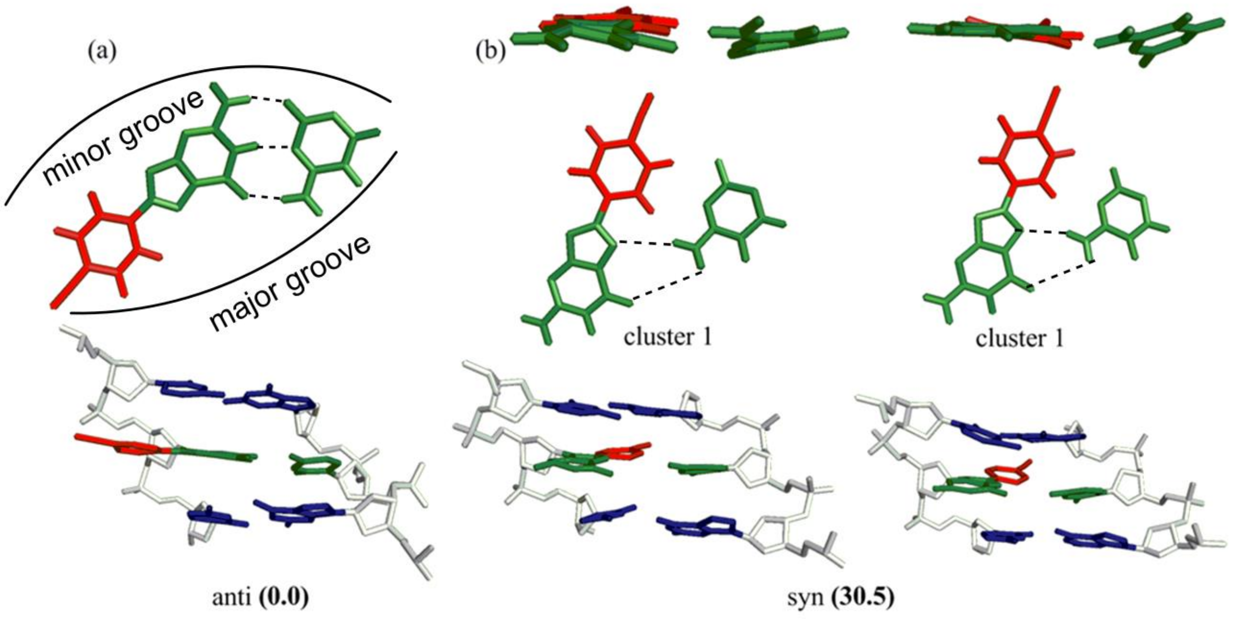

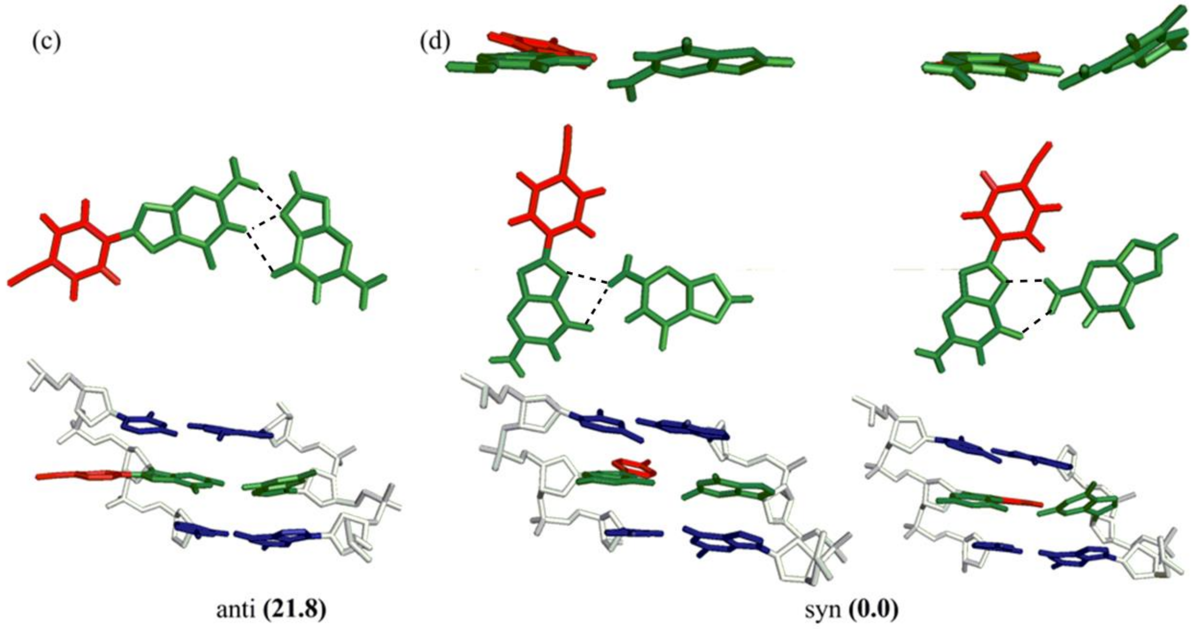

Figure S24. Portions of representative structures showing the orientation of the CNPhG adduct placed at G^3 position and paired against (a) C and (b) G in the *Nar*I recognition sequence. The hydrogen bonding pattern of the adduct is also shown explicitly for each of the structures. The corresponding free energy rankings are given in parentheses (bold) against each structure.

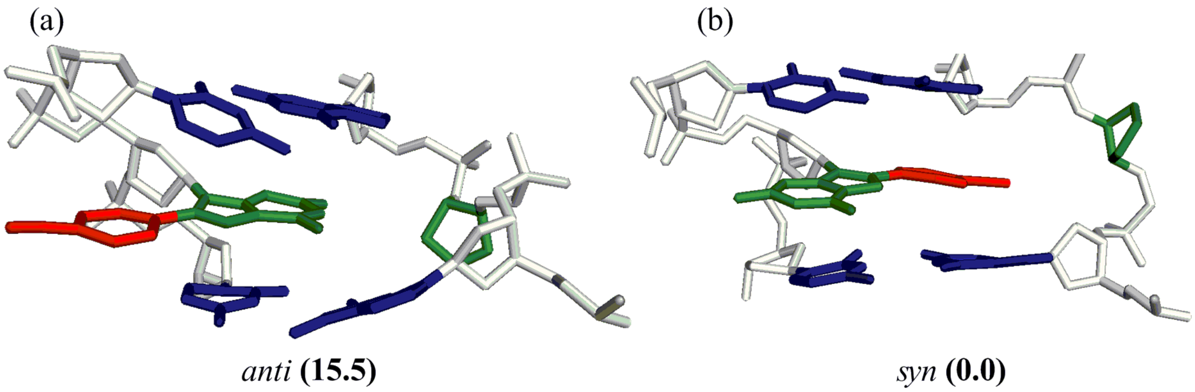

Figure S25. Portions of representative structures showing the orientation of the CNPhG adduct placed at G^3 position and paired against THF in the *Nar*I recognition sequence. The corresponding free energy rankings are given in parentheses (bold) against each structure.

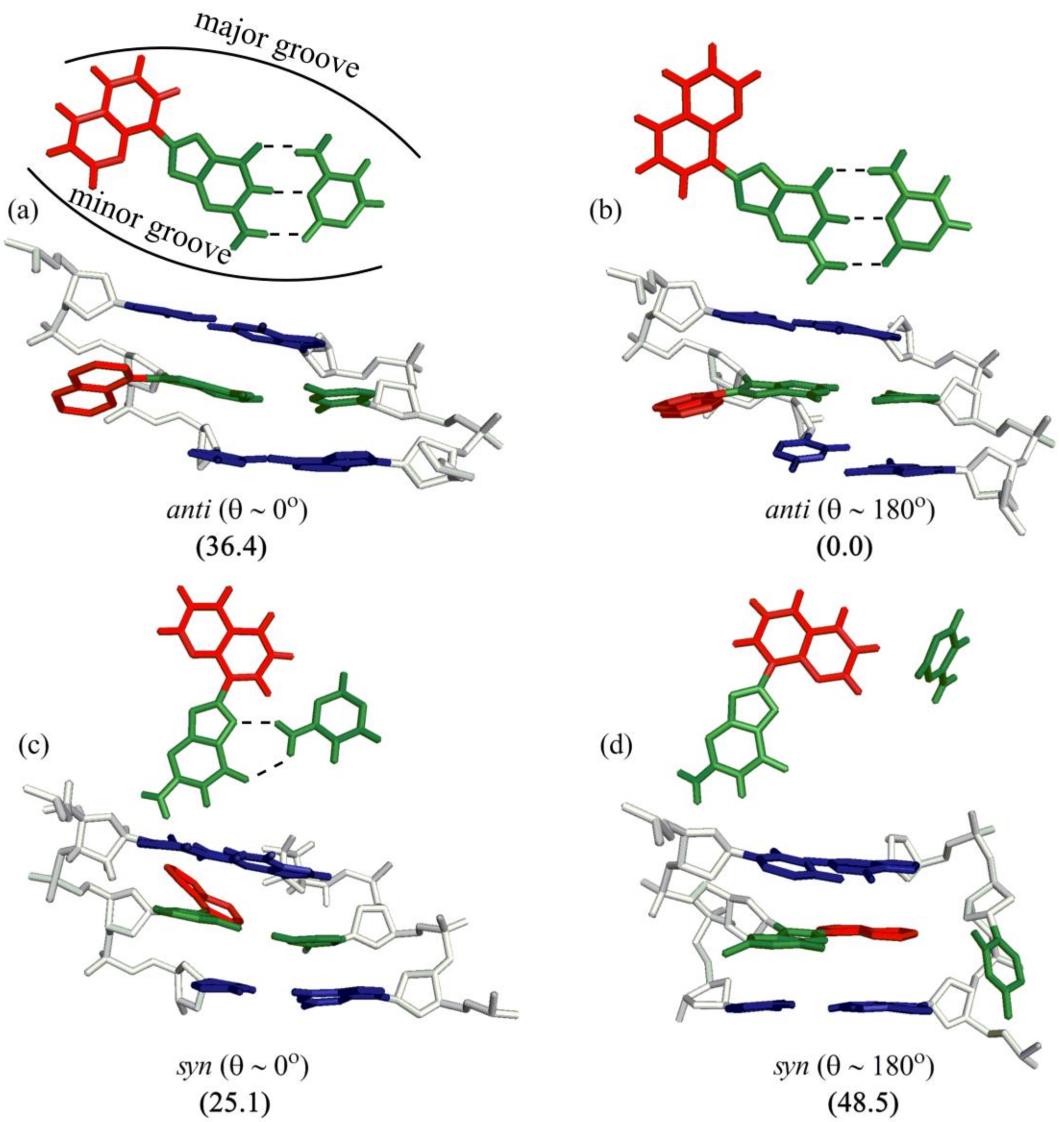

Figure S26. Portions of representative structures showing the orientation of the QG adduct placed at G^3 position and paired against C in the *Nar*I recognition sequence. The hydrogen bonding pattern of the adduct is also shown explicitly for each of the structures. The corresponding free energy rankings are given in parentheses (bold) against each structure.

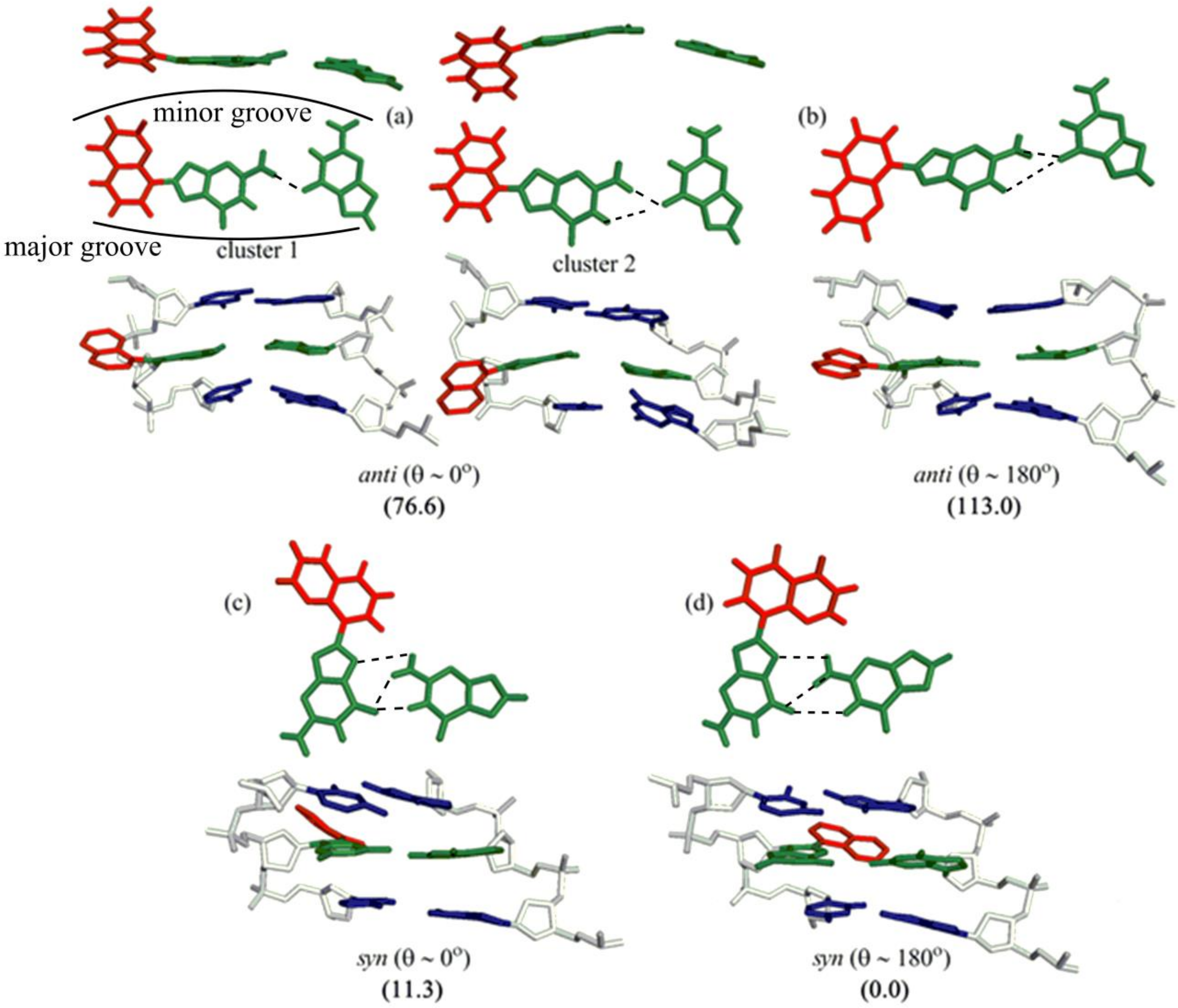

Figure S27. Portions of representative structures showing the orientation of the QG adduct placed at G^3 position and paired against G in the *Nar*I recognition sequence. The hydrogen bonding pattern of the adduct is also shown explicitly for each of the structures. The corresponding free energy rankings are given in parentheses (bold) against each structure.

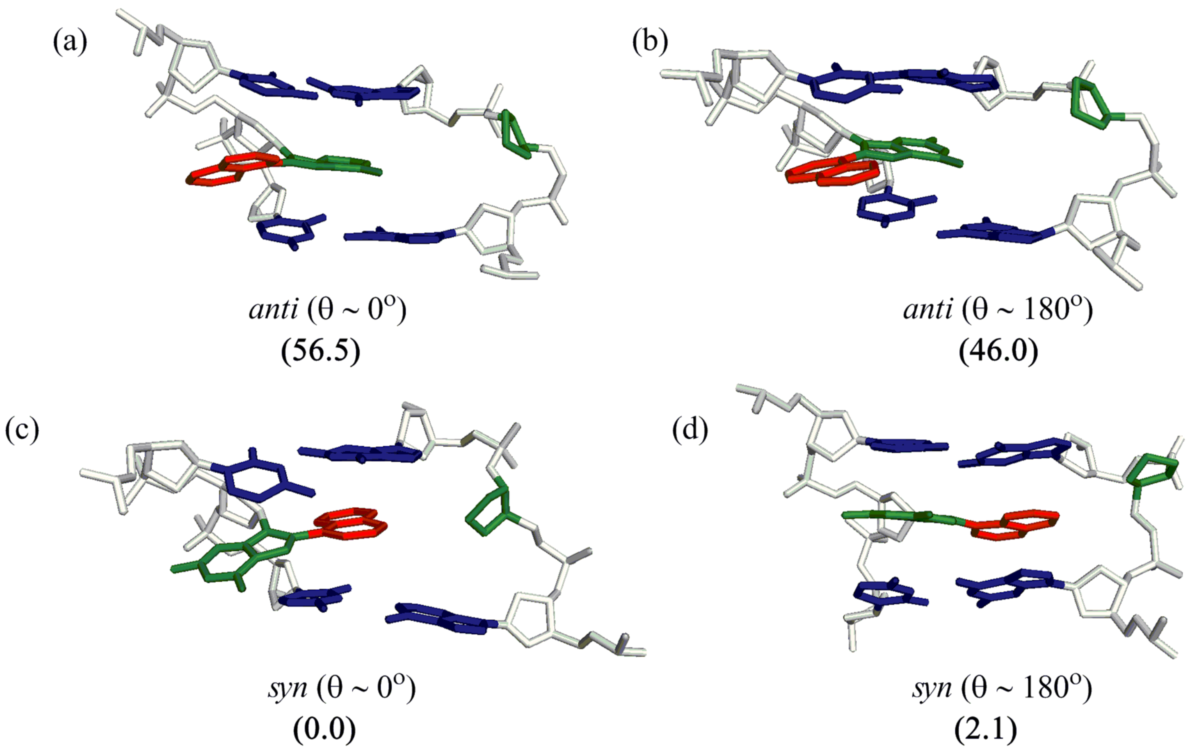

Figure S28. Portions of representative structures showing the orientation of the QG adduct placed at G^3 position and paired against THF in the *Nar*I recognition sequence. The corresponding free energy rankings are given in parentheses (bold) against each structure.

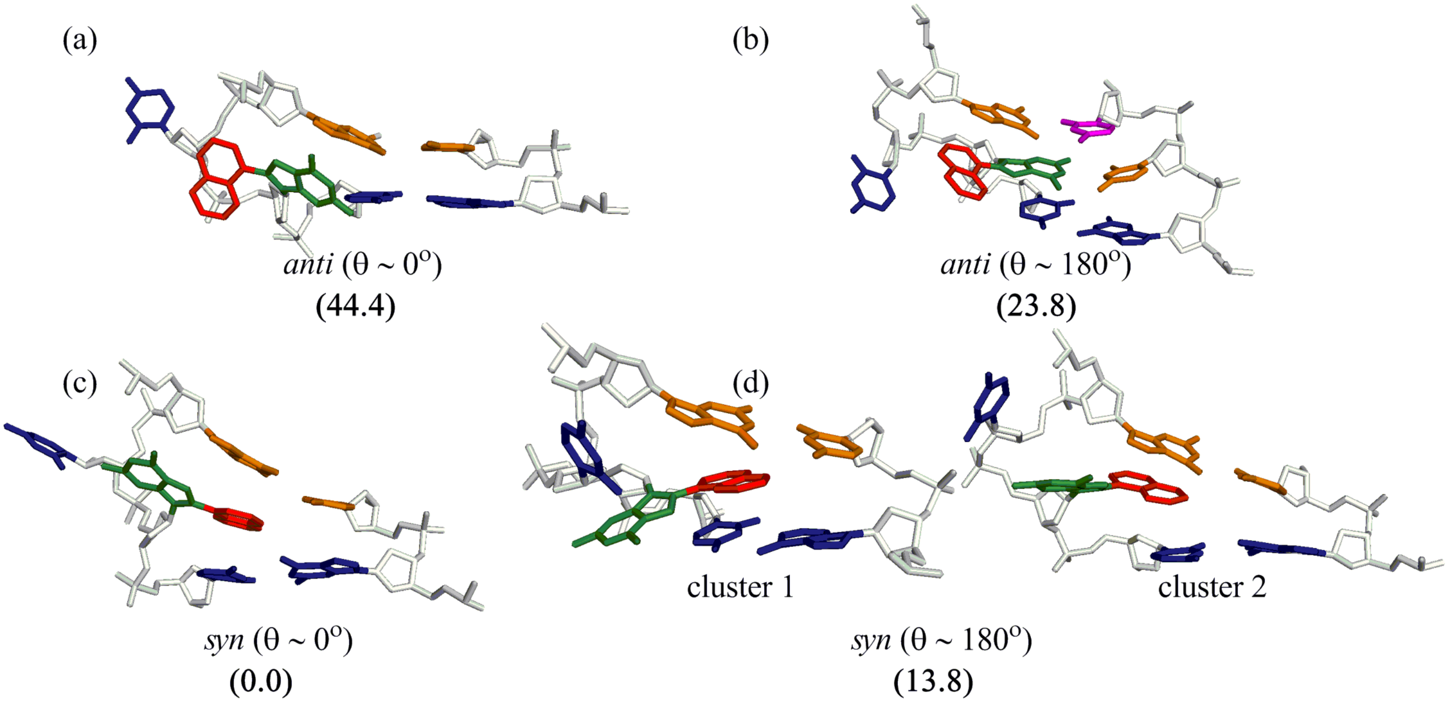

Figure S29. Portions of representative structures showing the orientation of the QG adduct placed at G^3 position and paired against -2 base deletion in the *Nar*I recognition sequence. The corresponding free energy rankings are given in parentheses (bold) against each structure.

Table S3. Free energy analysis of MD simulations of different adduct conformations.

adduct conformation	E[a] (kcal mol^{-1})	–TS[b] (kcal mol^{-1})	G[c] (kcal mol^{-1})	G_{rel}[d] (kcal mol^{-1})	G_{rel}[d] (kJ mol^{-1})
**FurG against C**					
anti (θ~0°)	-5027.4	-622.7	-5650.1	0.0	0.0
anti (θ~180°)	-5017.5	-621.3	-5638.8	11.3	47.3
syn (θ~0°)	-5024.4	-622.3	-5646.7	3.4	14.2
syn (θ~180°)	-5005.9	-622.4	-5628.3	21.8	91.2
**PhG against C**					
anti	-5027.4	-622.9	-5650.3	0.0	0.0
syn	-5021.9	-622.1	-5644.0	6.3	26.4
**CNPhG against C**					
anti	-5030.0	-623.0	-5653.0	0.0	0.0
syn	-5023.0	-622.7	-5645.7	7.3	30.5
**QG against C**					
anti (θ~0°)	-5013.6	-622.0	-5635.6	8.7	36.4
anti (θ~180°)	-5018.1	-626.2	-5644.3	0.0	0.0
syn (θ~0°)	-5015.7	-622.6	-5638.3	6.0	25.1
syn (θ~180°)	-5010.9	-621.8	-5632.7	11.6	48.5
**FurG against G**					
anti (θ~0°)	-4986.8	-622.4	-5609.2	5.7	23.8
anti (θ~180°)	-4981.0	-624.2	-5605.2	9.7	40.6
syn (θ~0°)	-4991.7	-623.2	-5614.9	0.0	0.0
syn (θ~180°)	-4987.4	-622.6	-5610.0	4.9	20.5
**PhG against G**					
anti	-4985.1	-622.9	-5608.0	8.9	37.3
syn	-4994.1	-622.8	-5616.9	0.0	0.0
**CNPhG against G**					
anti	-4985.2	-625.6	-5610.8	5.2	21.8
syn	-4988.8	-627.2	-5616.0	0.0	0.0
**QG against G**					
anti (θ~0°)	-4970.1	-625.0	-5595.1	18.3	76.6
anti (θ~180°)	-4965.1	-621.3	-5586.4	27.0	113.0
syn (θ~0°)	-4986.0	-624.7	-5610.7	2.7	11.3
syn (θ~180°)	-4989.4	-624.0	-5613.4	0.0	0.0
**FurG against THF**					
anti (θ~0°)	-4870.8	-618.2	-5489.0	14.8	62.0
anti (θ~180°)	-4874.3	-617.5	-5491.8	12.0	50.2
syn (θ~0°)	-4884.2	-619.6	-5503.8	0.0	0.0
syn (θ~180°)	-4878.6	-617.6	-5496.2	7.6	31.8
**PhG against THF**					
anti	-4985.1	-622.9	-5608.0	8.9	37.2
Syn	-4994.1	-622.8	-5616.9	0.0	0.0
**CNPhG against THF**					
anti	-4851.4	-617.1	-5468.5	3.7	15.5

Syn	-4856.5	-615.6	-5472.1	0.0	0.0
**QG against THF**					
anti (θ~0°)	-4832.9	-615.2	-5448.1	13.5	56.5
anti (θ~180°)	-4835.3	-615.2	-5450.5	11.0	46.0
syn (θ~0°)	-4843.3	-618.2	-5461.5	0.0	0.0
syn (θ~180°)	-4843.6	-617.4	-5461.0	0.5	2.1
**FurG against -2**					
anti (θ~0°)	-4551.5	-577.0	-5128.5	0.0	0.0
anti (θ~180°)	-4550.6	-576.9	-5127.5	1.0	4.3
syn (θ~0°)	-4550.4	-574.7	-5125.1	3.4	14.2
syn (θ~180°)	-4545.2	-573.7	-5118.9	9.6	40.2
**PhG against -2**					
Anti	-4527.1	-568.3	-5095.4	0.0	0.0
Syn	-4524.6	-567.2	-5091.8	3.6	15.1
**CNPhG against -2**					
anti	-4524.7	-571.0	-5095.7	3.3	13.8
syn	-4527.8	-571.2	-5099.0	0.0	0.0
**QG against -2**					
anti (θ~0°)	-4506.0	-576.8	-5082.8	10.6	44.4
anti (θ~180°)	-4511.9	-575.8	-5087.7	5.7	23.8
syn (θ~0°)	-4519.3	-574.1	-5093.4	0.0	0.0
syn (θ~180°)	-4515.2	-574.9	-5090.1	3.3	13.8

[a]total energy calculated using the molecular mechanics force field and Poisson-Boltzmann's approach. [b]entropy term calculated using normal mode analysis. [c]Total free energy, G= E – TS. [d]Relative free energy indicating the lowest energy structure from simulations with the particular adduct paired opposite a particular nucleobase (C or G).

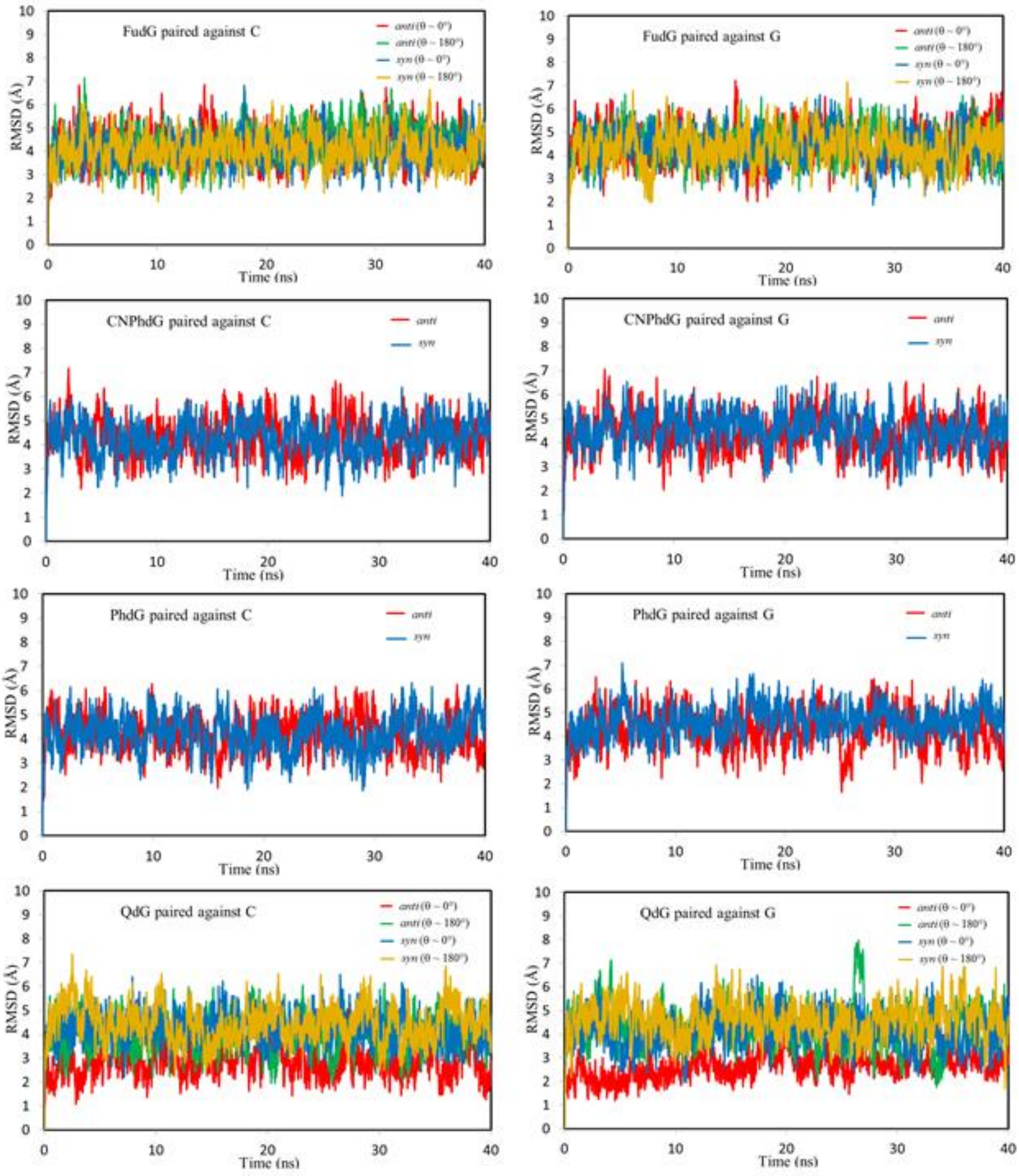

Figure S30. Backbone RMSD for MD simulations on the studied adducts in different conformations in the *Nar*I duplex against cytosine and guanine.

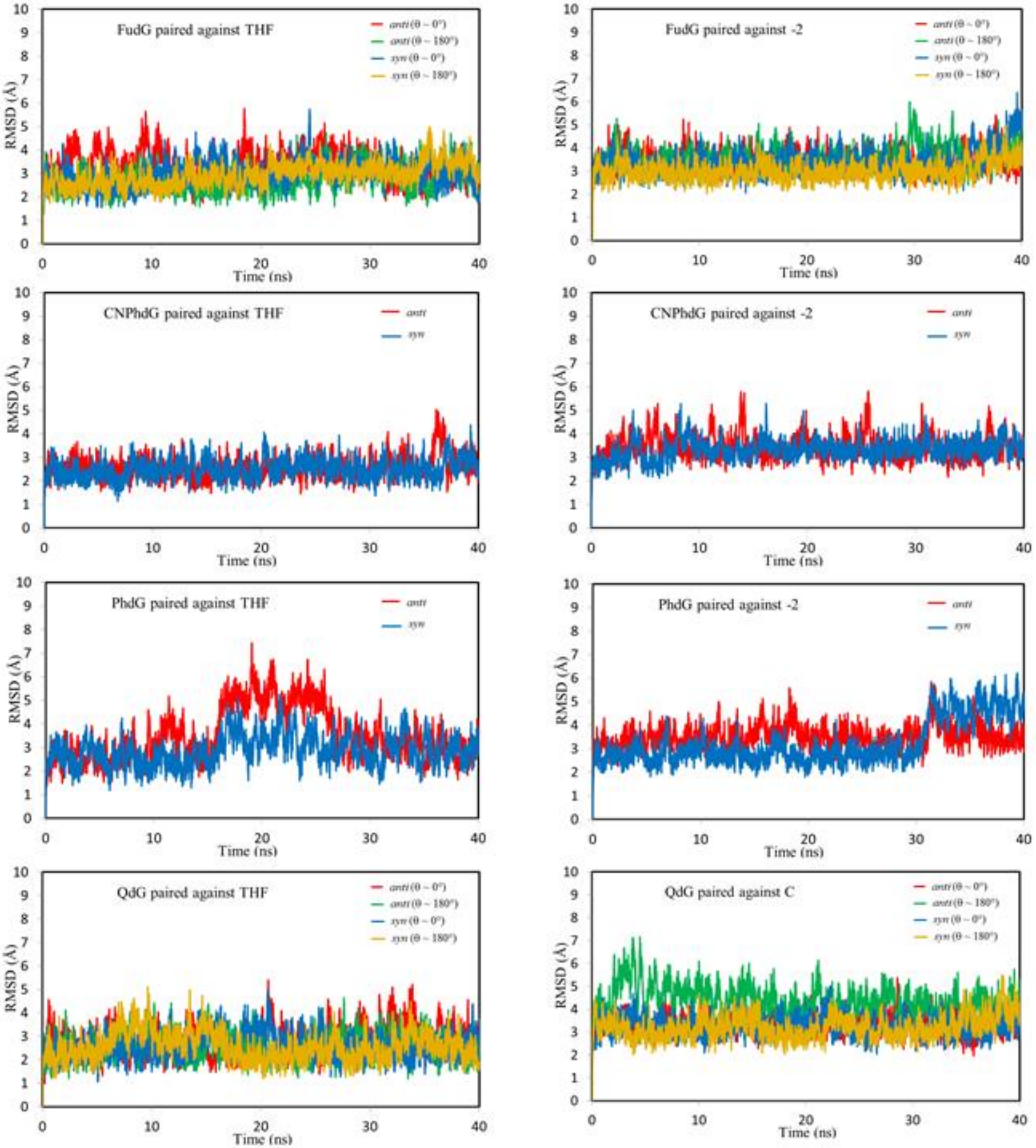

Figure S31. Backbone RMSD for MD simulations on the studied adducts in different conformations in the *Nar*I duplex against THF and -2 base deletion.

S32. Mol2 file for the QG nucleoside adduct.

```
@<TRIPOS>MOLECULE
LIG
   48    52     1     0     0
SMALL
No Charge or Current Charge
@<TRIPOS>ATOM
      1 P          4.6560   -0.0070   -1.1820 P         1 LIG      1.166100
      2 O          4.1660    1.0880    0.0000 OS        1 LIG     -0.497400
      3 O1         4.5600    0.6490   -2.5250 O2        1 LIG     -0.767700
      4 O2         5.8700   -0.6860   -0.6260 O2        1 LIG     -0.767700
      5 O3         0.0000    0.0000    0.0000 OS        1 LIG     -0.548200
      6 C5*        3.0950    1.9470   -0.3460 CI        1 LIG     -0.016400
      7 H5*1       3.3920    2.6860   -1.1090 H1        1 LIG      0.082800
      8 H5*2       2.8160    2.5000    0.5600 H1        1 LIG      0.082800
      9 C4*        1.8700    1.1960   -0.8680 CT        1 LIG      0.141700
     10 H4*        1.0270    1.8920   -0.9630 H1        1 LIG      0.107900
     11 O4*        2.1730    0.6600   -2.1750 OS        1 LIG     -0.404300
     12 C1*        1.9230   -0.7410   -2.2090 CT        1 LIG      0.144600
     13 H1*        0.9310   -0.9380   -2.6210 H2        1 LIG      0.090700
     14 C3*        1.4300   -0.0000   -0.0000 CT        1 LIG      0.155200
     15 H3*        1.8240    0.0800    1.0210 H1        1 LIG      0.072300
     16 C2*        2.0070   -1.2060   -0.7500 CT        1 LIG     -0.080700
     17 H2*1       3.0490   -1.3650   -0.4620 HC        1 LIG      0.050200
     18 H2*2       1.4490   -2.1310   -0.5880 HC        1 LIG      0.050200
     19 N          2.8810   -1.3580   -3.1220 N*        1 LIG     -0.041700
     20 C          2.6170   -2.0280   -4.3240 CK        1 LIG      0.219100
     21 C6         4.2450   -1.2000   -3.0780 CB        1 LIG      0.142100
     22 N1         3.7140   -2.2900   -5.0010 NB        1 LIG     -0.536700
     23 C7         4.7400   -1.7860   -4.2380 CB        1 LIG      0.209600
     24 N2         4.9500   -0.5640   -2.1040 NC        1 LIG     -0.487900
     25 C8         6.1600   -1.7790   -4.4840 C         1 LIG      0.447200
     26 C9         6.2480   -0.5310   -2.3100 CA        1 LIG      0.570800
     27 O5         6.8190   -2.2300   -5.4030 O         1 LIG     -0.534500
     28 N3         6.8370   -1.0990   -3.4060 NA        1 LIG     -0.429300
     29 H          7.8460   -1.1010   -3.4980 H         1 LIG      0.345500
     30 N4         7.0560    0.0580   -1.3690 N2        1 LIG     -0.844200
     31 H6         7.9200    0.4760   -1.6870 H         1 LIG      0.383900
     32 H7         6.5460    0.6180   -0.6910 H         1 LIG      0.383900
     33 C10        1.2780   -2.3670   -4.8490 CA        1 LIG     -0.023600
     34 C11        0.3070   -3.1180   -4.1080 CA        1 LIG      0.460300
     35 C12        1.0050   -2.0320   -6.1660 CA        1 LIG     -0.146700
     36 H8         1.7610   -1.4950   -6.7290 HA        1 LIG      0.179900
     37 C13       -0.9280   -3.4720   -4.7430 CA        1 LIG     -0.012100
     38 C14       -0.2160   -2.3790   -6.7820 CA        1 LIG     -0.176400
     39 H9        -0.3940   -2.0870   -7.8130 HA        1 LIG      0.163800
     40 C15       -1.8580   -4.2350   -3.9920 CA        1 LIG     -0.071900
     41 H10       -2.8050   -4.5140   -4.4470 HA        1 LIG      0.149900
     42 C16       -1.1720   -3.0800   -6.0840 CA        1 LIG     -0.232600
     43 H11       -2.1160   -3.3530   -6.5500 HA        1 LIG      0.176400
```

44 C17	-0.2930	-4.2240	-2.1790	CA	1	LIG	0.237600
45 H12	-0.0210	-4.5230	-1.1660	H4	1	LIG	0.091900
46 C18	-1.5450	-4.6180	-2.7100	CA	1	LIG	-0.281800
47 H13	-2.2290	-5.2100	-2.1090	HA	1	LIG	0.152900
48 N5	0.5970	-3.5040	-2.8320	NC	1	LIG	-0.557200

S33. Detailed Results from MD Simulations

Adducts paired against C. Against C, initial structures for the *anti*-conformations of the adducts were built to allow the Watson-Crick base pairing. Two possible orientations of the C8-moiety with respect to the nucleobase were considered for FurG and QG, which roughly correspond to θ ~ 0° or 180° and exhibit deviations from planarity of up to 20° to eliminate steric clashes with the DNA backbone (Table S1 and Figures S3−S6). Thus, the starting *anti*-orientations position the C8-moiety in the major groove, but differ in solvent exposure of the bulky substituent. Since the C8-moiety is symmetrical in the case of PhG and CNPhG (i.e., θ ~ 0° and θ ~180° correspond to the same structure), only one θ orientation was considered.

The initial models for the *syn* conformations of the adducts were also built by considering two orientations of the C8-moiety (θ ~0° and ~180°), with deviations from planarity ranging between 0 and 41° in order to eliminate steric clashes with the backbone (Table S1 and Figures S5-S8). Adducts in the *syn* orientation can give rise to two major conformations, namely the stacked (S) and the wedged (W) form. The W-type conformation positions the C8-moiety in the minor groove, while the S-type conformation intercalates the C8-moiety between the neighboring base pairs. The two θ-orientations used in the initial models of the *syn* conformations ensured that both W- and S-type structures were considered.

Analysis of the MD trajectories reveals that the *anti*-orientation of all adducts maintains Watson-Crick H-bonding with the complementary C (more than 98% occupancy of H-bonds;

Table S2), which places the C8-moiety in the major groove (Figures S18a,b, S22a,b, S24a,b and S26a,b). Over the course of the simulations, the χ dihedral angle of the adducts remains in the range of an *anti* conformation ($\chi \sim 220 - 250°$ for FurG, PhG and CNPhG, and $\chi \sim 200 - 250°$ for QG), whereas θ deviates from planarity by a maximum of 40° (Figures S10, S12, S14 and S16). However, depending on the size of the C8-moiety and the orientation of the θ-dihedral angle, the *syn*-conformations adopt different structural characteristics.

In the case of FurG, the ($\theta \sim 0°$) *syn* conformation displays a bifurcated Hoogsteen H-bond involving the amino group of the opposing C (more than 70% occupancy, Table S2), which locates the C8-moiety in the minor groove (Figure S18c). The χ value for this *syn* conformation remains in the range of 40 – 80°, and θ ranges between 0 and 10° (Figure S10). In the second ($\theta \sim 180°$) *syn* conformation, such Hoogsteen H-bonds are prevented by H-bonding between N4 of C and the oxygen atom of the furyl ring (~32% occupancy; Table S2). Despite the loss of Hoogsteen H-bonding, the C8-moiety stacks poorly with the neighboring bases, and remains in an extrahelical position in the minor groove (Figure S18d). The χ values remain between 30 and 50°, and θ deviates from planarity by a maximum of 20° over the course of the simulation (Figure S10d).

In contrast to FurG, the *syn*-conformations of PhG and CNPhG exhibit bimodal distributions with respect to χ, with two peaks at ~ 40° and ~ 80° (Figures S12a and S14a). Although the peak at 80° dominates over that at 40° in case of PhG, structures corresponding to both the peaks are almost equally probable in case of CNPhG. The representative structure corresponding to the cluster at $\chi \sim 40°$ displays weaker Hoogsteen H-bonding (as indicated by larger donor-acceptor distances) compared to the cluster at $\chi \sim 80°$ (Figures S22b and S24b). Overall, the Hoogsteen H-bonds persist for greater simulation time in case of PhG (both H-bonds remain intact for at

least 59% of the simulation time compared to CNPhG (both H-bonds remain intact for at least 44% of the simulation time, Table S2). In addition, the structure mostly remains in a W-type conformation with poor stacking between the phenyl or cyanophenyl moiety and the neighboring base pairs.

The bulkier QG adduct can adopt both W- and S-type structures in the *syn* orientation. When *syn*-QdG adopts θ ~ 0°, a W-type structure is found that contains two Hoogsteen H-bonds between N7 and O6 of QG and the amino group of the opposing C (77% and 53% occupancy respectively; Table S2 and Figure S26c). On the other hand, *syn*-QG with θ ~ 180° yields a base-displaced intercalated structure (Figure S26d). Both the W- and S-structures exhibit a unimodal distribution with respect to the χ and θ dihedral angles of QG, where the W-structure mostly adopts χ values ranging from 60 – 80° and the S-type structures adopt χ values between 30 and 60° throughout the simulation (Figure S16). However, the C8-moiety is more planar in the S-conformation (θ deviates from planarity by less than 10°; Figure S16d) in order to maximize stacking with the neighboring bases. In contrast, θ deviates from planarity by up to 20 – 30° in the W-structure (Figure S16b), where the increased flexibility arises since the C8-moiety is located in the minor groove.

The calculated free energies (Table S3) suggest that PhG, CNPhG and QG favor the *anti* conformation by at least 25 kJ mol^{-1} compared to the *syn* conformation(s). This indicates that the loss of Watson-Crick H-bonding upon adopting the *syn* conformation is not adequately compensated by relatively weaker Hoogsteen H-bonding and/or stacking interactions with the C8-moiety.

Adducts paired against G. In order to understand the structural characteristics of guanine mismatch stabilization by the C-linked adducts, adducts in the *syn* orientation were paired against

anti-G, while the *anti*-orientations of the adducts were paired against *syn*-G. Initial structures of the adducts paired against G were prepared from the corresponding initial structures of the adducts paired against C by replacing C with G and slightly adjusting θ and χ of the adduct to allow H-bonding interactions with the opposing G. Details of the initial structures used in the simulations are provided (Table S1 and Figures S3b, S4b, S5b and S6b).

In the (θ ~ 0° and 180°) *anti* conformations, the FurG adduct forms Hoogsteen H-bonds with the opposing *syn*-G, where the N2-H(FurG)...N7 H-bond persists for ~97% of the simulation time (Figure S19a,b and Table S2). In addition, two relatively flexible H-bonds between N1 of FurG and the O6 and N7 atoms of G (45–62% occupancy) stabilize these structures. Overall, the χ dihedral angle remains in the range of an *anti* conformation, while θ deviates from planarity by less than 10° in both simulations (Figures S10e-h). Similarly, the *anti* conformations of the PhG and CNPhG adducts also displays Hoogsteen H-bonding (see Table S2 for occupancies and Figures S22c, S23c for representative structures), where χ ranges from 220 – 240°, and θ deviates from planarity by up to 20° (Figures S12c,d and S14c,d). However, in its *anti* conformation, the QG adduct exhibits a bimodal distribution with respect to χ and θ when θ ~ 0°, and a unimodal distribution with respect to both dihedral angles when θ ~ 180° (Figure S16). The representative structures from the bimodal distribution at θ ~ 0° are related by a 20° flip of θ from planarity in opposite orientations, and differ with respect to χ by ~50° (Figures S16e,f and S27a). However, both structures display only one interbase H-bond involving N2 of QG and O6 of G, which persists for ~90% of the total simulation time (Table S2). On the other hand, the structure at θ ~ 180° displays a bifurcated Hoogsteen H-bonding between O6 of G, and the N2 and N1 donors of QG (~93% and ~56% occupancy respectively, Table S2). In this structure, χ ranges from 260 – 280°, and θ ranges from 160 – 180° (Figures S16g,h and S27b).

The *syn* conformation of FurG with initial θ ~ 0° displays a unimodal distribution with respect to θ and χ, while the conformation with initial θ ~ 180° yields a bimodal distribution with respect to χ (where one of the peaks is dominant) and a unimodal distribution with respect to θ (Figure S10e-h). The θ ~ 0° structure possesses (two) bifurcated Hoogsteen H-bonds involving O6 of guanine and N1 and N2 donors of FurG (~72% occupancy, Table S2) and a weaker interaction between the amino group of G and N7 of FurG (~55% occupancy, Table S2) due to steric hindrance of the CH groups of the furyl moiety and the amino group of opposing G (Figure S19c). On the other hand, the simulation with initial θ ~ 180° displays two persistent Hoogsteen H-bonds (more than 81% occupancy, Table S2). One of the clusters corresponding to bimodal χ distribution obtained from this simulation exhibits greater buckling of the FurG:G Hoogsteen base pair, which is responsible for the difference in χ between the two clusters (Figure S19d).

On the other hand, PhG adopts two *syn* conformations that differ in their glycosidic torsion angle (χ ~ 40° and χ ~ 80°) and H-bonding interactions. The conformation at χ ~ 80° maintains Hoogsteen H-bonding between PhG or CNPhG and the opposing *anti*-G. In contrast, the orientation at χ ~ 40° weakens H-bonding between the amino group of G and N7 of PhG or CNPhG, which locates the C8-phenyl group towards the interior of the helix and enhances stacking interactions with the 5′-neighboring base pair (Figure S22d). Whereas the conformation at χ ~ 80° is dominant in case of PhG, CNPhG prefers the conformation at χ ~ 40° (Figures S12c and S14c). This is the reason why the occupancies of Hoogsteen H-bonds are greater in PhG than CNPhG (Table S2), which directly affects their *anti*/*syn* free energy differences (Table S3).

The QG adduct also acquires two *syn* conformations (corresponding to θ ~ 0° and θ ~180°), which have different H-bonding patterns (Figure S27), but a similar range of χ values and

unimodal distributions with respect to θ and χ (Figure S16e-h). The structure at θ ~ 0° displays a similar Hoogsteen H-bonding pattern as FurG, where the bifurcated H-bonding between O6 of the QG and N2 and N1 of G persists for more than 72% of the simulation time and the H-bond between the amino group of G and N7 of the adduct persists for ~56% of the simulation time (Table S2 and Figure S27c). However, an additional H-bond between the nitrogen atom of the quinolyl ring and the amino group of the opposing G is formed when θ ~ 180° (Figure S27d), which persists for ~93% of the simulation time (Table S2). This weakens the N1-H(G)...O6(QG) H-bond (~31% occupancy), and leads to a bifurcated H-bonding pattern between the amino group of the opposing G (~93% and ~99% occupancy of H-bonds involving O6 and nitrogen atom of quinolyl ring of QG, respectively). The calculated free energies indicate all four adducts prefer a *syn* conformation when paired opposite a G mismatch by 24 to 113 kJ mol^{-1} depending on the identity of the C8-substituent (Table S3).

Adducts paired against THF. In order to understand the tendency of the adducts to intercalate within the DNA helices, the adducts in their *syn* and *anti* conformations were paired against an abasic site. The tetrahydrofuran moiety, which excludes the –OH group present in the abasic site, was used as a model for mimicking the abasic site residue. Initial structures of the adducts paired against abasic site were prepared from the corresponding initial structures of the adducts paired against C by removing cytosine nucleobase moiety from the nucleotide opposing the adduct and substituting it with a hydrogen atom. In addition, wherever required, χ and θ dihedral angles were adjusted to allow optimal interactions. Details of the initial structures used in the simulations are provided in Tables S1 and Figures S7 and S9a.

In the *anti* conformations of all the adducts (which include both θ ~ 0° and 180° conformations of FurG and QG), the C8-subsituent remains in the major groove, and has little possibility of

forming additional stacking interactions with the flanking bases (Figures S20a,b, S23a, S25a and S28a,b). Over the course of simulations, all the adducts acquire unimodal distributions with respect to χ and θ, indicating that only one conformation is dominant for all the adducts (Figures S11a-d, S13a,b, S15a,b and S17a-d). On the other hand, when paired in their *syn* conformations against THF, the adducts have greater propensity to intercalate their C8-substituents between the flanking base pairs within the DNA helix (Figures S20c,d, S23b, S25b and S28a,b). All the adducts acquire unimodal distribution with respect to χ and θ over the course of simulations (Figures S11a-d, S13a,b, S15a,b and S17a-d). The calculated relative free energies for different conformations indicate that all the adducts prefer *syn* conformations against THF, where the *syn*-preference mainly arises due to additional stacking interactions with the flanking bases.

Adducts paired against −2. In order to analyse the tendency of the adducts to stabilize -2 base mutations within the DNA helices, the DNA strands containing the adducts in their *syn* and *anti* conformations were paired against 10-mer strands, thereby introducing a 2-base bulge. The initial structures for the simulations were built based on the NMR structure of C8-bonded aminofluorene-dG adduct (PDB code: 1AX6) by removing the C8-moiety are replacing it with Fur, Ph, CNPh or Q. Details of the initial structures used in the simulations are provided in Table S1 and Figures S8 and S9b.

In one of its *anti*-conformations ($\theta \sim 0°$), FurG is stabilized through H-bonding between the cytosine present on the opposite strand and 5′ to the bulge. However, this interaction disrupts the H-bonding between this cytosine and its complementary guanine (orange bases in Figure S21a). On the other hand, the structure at $\theta \sim 180°$ retains the base pair 5′ to the bulge (orange base pair in Figure S21b), and stabilizes the adduct through additional stacking interactions. Similarly, stabilizing stacking interactions are also observed in one of the *syn* conformations ($\theta \sim 0°$) of the

adduct (Figure S21c). However, the adducted nucleotide stacks poorly in the *syn* conformation at θ ~ 180° (Figure S21d). Overall, the *anti* conformation (θ ~ 0°) which stabilize the adduct through H-bonding interactions rank lower in energy than the *anti*- (θ ~ 180°) and *syn*- (θ ~ 0°) conformations (by 4-14 kJ mol^{-1}), where the adduct is stabilized through stacking interactions. However, with poor stacking interactions, the *syn* conformation at θ ~ 180° ranks highest in energy (Table S3).

The *anti* conformation of PhG adduct against -2 deletion is similar to the *anti* conformation of FurG at θ ~ 0°, where the H-bonding interactions are observed between the adduct and the cytosine present on the opposite strand and 5′ to the bulge (Figure 6). Similarly, the *syn* conformation of PhdG against deletion is similar to the *syn* conformation of FurG at θ ~ 0°, where the adduct is stabilized through stacking interaction with the flanking base pairs. Overall, the *anti* conformation of PhG is more stable against the -2 deletion, where *anti*/*syn* energy difference is smaller than FurG (Table S3). Although the *anti* conformation of the CNPhG adduct is also stabilized through H-bonding with the cytosine at the same position, the *syn* conformation attains greater stability due to penetration of the unpaired cytosine present 5′ to the adduct into the helix. This provides additional stacking stabilization to the adduct, which results in the *syn* conformation being 14 kJ mol^{-1} more stable than the corresponding *anti* conformation.

In case of QG, the *anti* conformation of the adduct at θ ~ 0° neither forms H-bonding interactions with any of the base on the opposite strand nor stacks with the bases on the same strand (Figure S29a). On the other hand, the *anti* conformation at θ ~ 180° is stabilized through Watson-Crick H-bonding between the adduct and the cytosine present on the opposite strand and 5′ to the bulge (Figure S29b). However, such a pairing also disrupts the other base pairing interaction 5′ to the lesion site (e.g. see interaction between orange and pink bases in Figure

S29b, which disrupts the normal Watson-Crick pairing between two pink bases). In contrast, both the *syn* conformations ($\theta \sim 0°$ and $\theta \sim 180°$) stabilize the adduct by stacking the quinolyl moiety within the helix. Whereas the simulation at $\theta \sim 0°$ exhibit a unimodal distribution with respect to θ and χ dihedral angles, the structure at $\theta \sim 180°$ exhibits a bimodal distribution with respect to χ and θ (Figure S17). The two clusters of the bimodal distribution differ in the extent of stacking between the quinolyl moiety and the flanking bases. Overall, the *syn* conformations of QG are lower in energy than the *anti* conformations due to additional stacking interactions provided by the bulky C8-substituent. Specifically, the *syn* conformation at $\theta \sim 0°$ is the overall lowest energy conformation, due to most optimal stacking interaction between the adduct and the DNA helix.

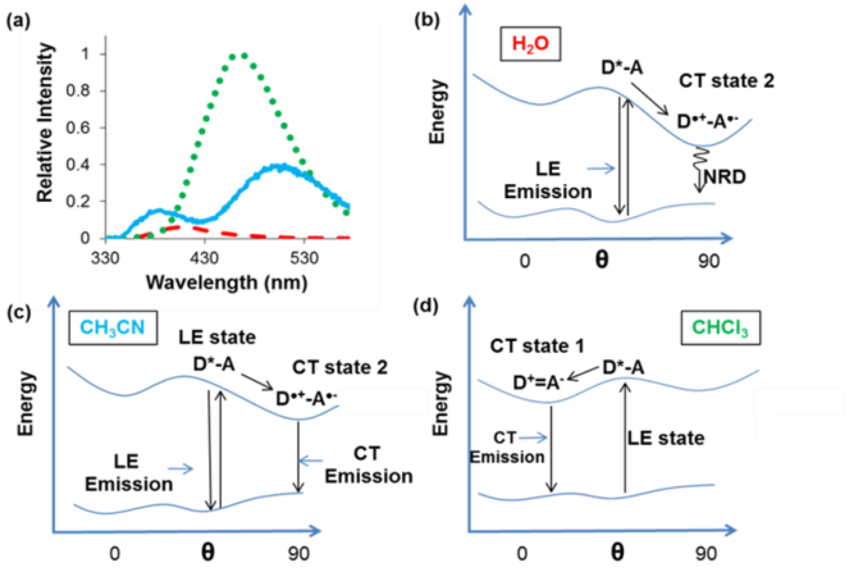

Figure S34. (a) Emission spectra of QG in H_2O (dashed red trace), CH_3CN (solid blue trace) and $CHCl_3$ (dotted green trace). Proposed energy-coordinate diagrams for the emissive properties of QG in: (b) H_2O, (c) CH_3CN and (d) $CHCl_3$.

Rationale: In H_2O, QG displays quenched emission at 407 nm (Φ_{fl} = 0.03) following excitation at 313 nm (Table 2 in manuscript). In CH_3CN, dual emission was observed at 384 and 510 nm (Φ_{fl} = 0.05), while in $CHCl_3$ relatively intense emission was detected at 468 nm (Φ_{fl} = 0.19). We propose that excitation of QG results in a biradicaloid ($D^{\bullet+}$-$A^{\bullet-}$) CT state being readily populated. This CT state is not emissive in water as it undergoes dynamic quenching. Therefore, locally excited (LE) emission is observed at 407 nm, which is attributed to the relatively small population of excited state QG that did not undergo CT processes and equilibrate to the lower energy CT state (Figure S34b). In polar aprotic CH_3CN the perpendicular geometry of $D^{\bullet+}$-$A^{\bullet-}$ is stabilized in the polar solvent; the CT emission is not quenched in aprotic solvents as it was in water, and dual emission is observed from LE and CT (Figure S34c). When QG was excited in $CHCl_3$ an emissive CT state at 468 nm was observed (dotted green trace in Figure S34a). We

propose that the emission resulted from a charge-separated planar $D^{+}=A^{-}$ CT state that is lower in energy than either the perpendicular CT or the LE state.

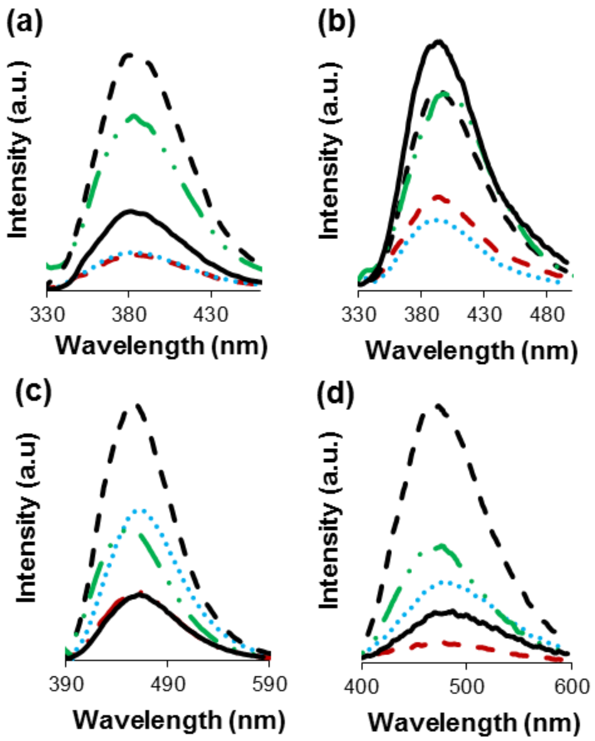

Figure S35. Fluorescence emission spectra of C8-aryl-G modified *Nar*I(12), in the single-strand (solid black trace), or hybridized to its complementary strand, N = C (dashed red trace), N = G (dotted blue trace), N = THF (dashed-dotted green trace) and N = −2 (dashed black trace), with (a) X = FurG, (b) X = PhG, (c) X = CNPhG, and (d) X = QG. All spectra of single-strand and duplex (6 μM) were recorded in 50 mM sodium phosphate buffer, pH 7, with 0.1 M NaCl at 10ºC.

S35. Crystal structure analysis of QG monohydrate

A crystal of QG (pale-yellow thick plate, 0.4x0.4x0.25 mm) was selected from a bulk crystalline product isolated from water-acetonitrile. The crystal was mounted on a Mitegen probe and studied at 150K on a SuperNova Agilent single-crystal diffractometer equipped with a microfocus CuK_α (λ = 1.54184 Å) radiation source and Atlas CCD detector. Diffraction intensity data were collected using ω-scan to the maximum 2θ angle of 148.7^o (resolution of 0.8Å), with the redundancy factor of 16. The unit cell parameters were refined using the entire data set.

The data were processed using CrysAlisPro software [A]. Absorption corrections were applied using the multiscan method. The structure was solved (direct methods) and refined (full-matrix least-squares on F^2) using SHELXL-97 [B]. Non-hydrogen atoms were refined anisotropically. Hydrogen atoms were refined isotropically; initially they were introduced at calculated positions but later their coordinates were refined. Geometric calculations were carried out using the WinGX [C] and Olex [D] software packages.

Crystal data and experimental parameters are summarized in Table S4. Full data for the studied structure, including a CIF-file, have been deposited with the Cambridge Crystallographic Data Centre (no. 1013599) and a copy of these data are available free of charge upon request from the CCDC web-site: http://www.ccdc.cam.ac.uk/data_request/cif or by e-mail: deposit@ccdc.cam.ac.uk.

References

[A] Agilent Technologies (2011) *Agilent Technologies, Xcalibur CCD system, CrysAlisPro Software system*; Version 1.171.35.8.
[B] Sheldrick, G.M. (1997) *SHELXL-97, Program for refinement of crystal structures*; University of Göttingen, Germany.
[C] Farrugia, L.J. *J. Appl. Crystallogr.* **1999**, *32*, 837-838.
[D] Dolomanov, O.V.; Bourhis, L.J.; Gildea, R.J.; Howard, J.A.K.; Puschmann, H. *J. Appl. Crystallogr.* **2009**, 42, 339-341.

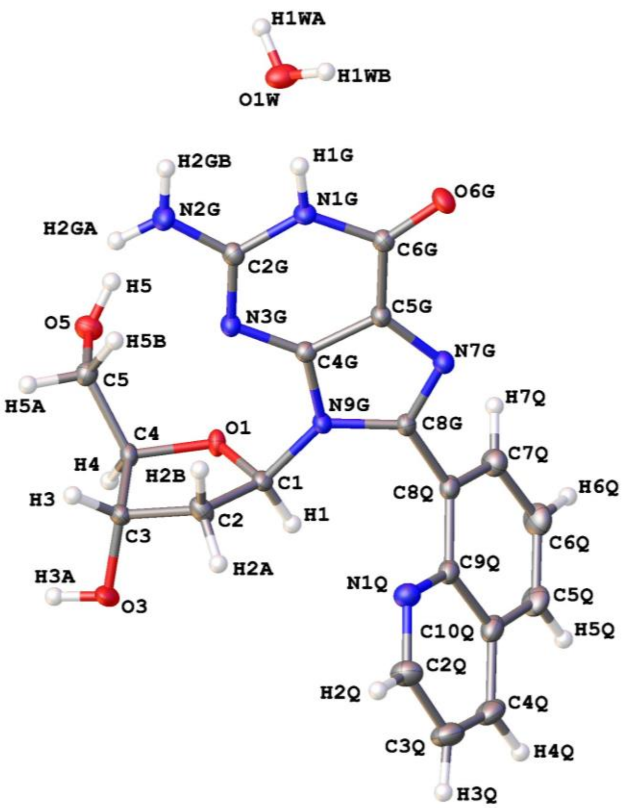

Table S4. Crystal data and structure refinement for QG•$H_2$0.

Empirical formula	C19 H20 N6 O5
Formula weight	412.41
Temperature	150 K
Wavelength	1.54184 A
Crystal system, space group	Orthorhombic, P 21 21 21
Unit cell dimensions	a = 6.68426(5) A
	b = 9.28108(8) A
	c = 29.6696(2) A
Volume	1840.62(2) A^3
Z, Calculated density	4, 1.488 Mg/m^3
Absorption coefficient	0.930 mm^-1
F(000)	864
Crystal size	0.40 x 0.40 x 0.25 mm
Theta range for data collection	4.99 to 75.84 deg.
Limiting indices	-8<=h<=8, -11<=k<=11, -37<=l<=37
Reflections collected / unique	36075 / 3837 [R(int) = 0.0221]
Completeness to theta = 75.84	99.6 %
Absorption correction	Semi-empirical from equivalents
Max. and min. transmission	0.8008 and 0.7074
Refinement method	Full-matrix least-squares on F^2
Data / restraints / parameters	3837 / 0 / 331
Goodness-of-fit on F^2	1.105
Final R indices [I>2sigma(I)]	R1 = 0.0251, wR2 = 0.0647
R indices (all data)	R1 = 0.0251, wR2 = 0.0647
Absolute structure parameter	0.06(11)
Largest diff. peak and hole	0.198 and -0.196 e.A^-3

Table S5. Atomic coordinates (x 10^4) and equivalent isotropic displacement parameters (A^2 x 10^3) for QG•$H_2$0.
U(eq) is defined as one third of the trace of the orthogonalized Uij tensor.

	x	y	z	U(eq)
C(1)	8682(2)	-350(1)	1249(1)	15(1)
C(2)	7103(2)	-1350(1)	1049(1)	20(1)
C(2G)	8734(2)	1223(1)	-131(1)	15(1)
C(2Q)	4230(2)	729(1)	2172(1)	26(1)
C(3)	8150(2)	-2790(1)	994(1)	18(1)
C(3Q)	4335(2)	801(2)	2649(1)	30(1)
C(4)	10381(2)	-2401(1)	961(1)	16(1)
C(4G)	8617(2)	1483(1)	613(1)	14(1)
C(4Q)	5934(2)	1463(1)	2839(1)	28(1)
C(5)	11219(2)	-2441(1)	488(1)	17(1)
C(5G)	8605(2)	2973(1)	605(1)	15(1)
C(5Q)	9173(2)	2746(1)	2739(1)	26(1)
C(6G)	8606(2)	3684(1)	178(1)	15(1)
C(6Q)	10577(2)	3324(2)	2457(1)	28(1)
C(7Q)	10357(2)	3194(1)	1984(1)	23(1)
C(8G)	8608(2)	2389(1)	1303(1)	15(1)
C(8Q)	8745(2)	2485(1)	1799(1)	17(1)
C(9Q)	7239(2)	1908(1)	2088(1)	17(1)
C(10Q)	7465(2)	2042(1)	2563(1)	22(1)
N(1G)	8696(1)	2697(1)	-176(1)	16(1)
N(1Q)	5602(2)	1262(1)	1899(1)	20(1)
N(2G)	8844(2)	451(1)	-513(1)	20(1)
N(3G)	8666(1)	555(1)	263(1)	15(1)
N(7G)	8602(2)	3529(1)	1039(1)	16(1)
N(9G)	8592(1)	1107(1)	1064(1)	14(1)
O(1)	10595(1)	-963(1)	1156(1)	16(1)
O(3)	7758(2)	-3673(1)	1378(1)	28(1)
O(5)	13342(1)	-2270(1)	505(1)	20(1)
O(6G)	8540(1)	4986(1)	99(1)	20(1)
O(1W)	8429(1)	3609(1)	-1068(1)	25(1)

Table S6. Bond lengths [A] and angles [deg] for QG•$H_2$0.

C(1)-O(1)	1.4266(13)
C(1)-N(9G)	1.4603(12)
C(1)-C(2)	1.5256(15)
C(1)-H(1)	0.957(15)
C(2)-C(3)	1.5175(15)
C(2)-H(2A)	0.984(16)
C(2)-H(2B)	0.982(16)
C(2G)-N(3G)	1.3245(13)
C(2G)-N(2G)	1.3416(14)
C(2G)-N(1G)	1.3743(13)
C(2Q)-N(1Q)	1.3191(16)
C(2Q)-C(3Q)	1.4179(17)

C(2Q)-H(2Q)	0.974(17)
C(3)-O(3)	1.4267(13)
C(3)-C(4)	1.5376(15)
C(3)-H(3)	1.021(15)
C(3Q)-C(4Q)	1.357(2)
C(3Q)-H(3Q)	0.989(18)
C(4)-O(1)	1.4615(12)
C(4)-C(5)	1.5093(14)
C(4)-H(4)	0.998(15)
C(4G)-N(3G)	1.3497(13)
C(4G)-C(5G)	1.3828(14)
C(4G)-N(9G)	1.3835(13)
C(4Q)-C(10Q)	1.4176(18)
C(4Q)-H(4Q)	0.987(16)
C(5)-O(5)	1.4292(13)
C(5)-H(5A)	1.005(15)
C(5)-H(5B)	1.000(15)
C(5G)-N(7G)	1.3893(13)
C(5G)-C(6G)	1.4274(14)
C(5Q)-C(6Q)	1.3671(19)
C(5Q)-C(10Q)	1.4148(18)
C(5Q)-H(5Q)	0.943(16)
C(6G)-O(6G)	1.2321(13)
C(6G)-N(1G)	1.3954(13)
C(6Q)-C(7Q)	1.4152(16)
C(6Q)-H(6Q)	1.022(17)
C(7Q)-C(8Q)	1.3770(16)
C(7Q)-H(7Q)	0.955(16)
C(8G)-N(7G)	1.3150(13)
C(8G)-N(9G)	1.3857(12)
C(8G)-C(8Q)	1.4767(13)
C(8Q)-C(9Q)	1.4267(15)
C(9Q)-N(1Q)	1.3672(15)
C(9Q)-C(10Q)	1.4235(15)
N(1G)-H(1G)	0.912(15)
N(2G)-H(2GA)	0.885(16)
N(2G)-H(2GB)	0.886(16)
O(3)-H(3A)	0.89(2)
O(5)-H(5)	0.859(18)
O(1W)-H(1WA)	0.88(2)
O(1W)-H(1WB)	0.90(2)

O(1)-C(1)-N(9G)	109.47(8)
O(1)-C(1)-C(2)	107.55(8)
N(9G)-C(1)-C(2)	112.87(9)
O(1)-C(1)-H(1)	105.4(9)
N(9G)-C(1)-H(1)	107.5(8)
C(2)-C(1)-H(1)	113.7(9)
C(3)-C(2)-C(1)	105.02(9)
C(3)-C(2)-H(2A)	111.0(9)
C(1)-C(2)-H(2A)	109.8(9)
C(3)-C(2)-H(2B)	110.9(9)
C(1)-C(2)-H(2B)	109.1(9)
H(2A)-C(2)-H(2B)	110.9(13)
N(3G)-C(2G)-N(2G)	119.78(10)
N(3G)-C(2G)-N(1G)	123.42(9)
N(2G)-C(2G)-N(1G)	116.80(9)

N(1Q)-C(2Q)-C(3Q)	124.04(12)
N(1Q)-C(2Q)-H(2Q)	118.5(10)
C(3Q)-C(2Q)-H(2Q)	117.5(10)
O(3)-C(3)-C(2)	109.56(9)
O(3)-C(3)-C(4)	111.37(9)
C(2)-C(3)-C(4)	104.30(9)
O(3)-C(3)-H(3)	110.9(9)
C(2)-C(3)-H(3)	112.4(9)
C(4)-C(3)-H(3)	108.2(9)
C(4Q)-C(3Q)-C(2Q)	118.45(12)
C(4Q)-C(3Q)-H(3Q)	122.4(10)
C(2Q)-C(3Q)-H(3Q)	119.2(10)
O(1)-C(4)-C(5)	110.75(8)
O(1)-C(4)-C(3)	106.49(8)
C(5)-C(4)-C(3)	114.43(9)
O(1)-C(4)-H(4)	104.6(8)
C(5)-C(4)-H(4)	110.1(8)
C(3)-C(4)-H(4)	110.0(9)
N(3G)-C(4G)-C(5G)	128.69(10)
N(3G)-C(4G)-N(9G)	125.68(9)
C(5G)-C(4G)-N(9G)	105.63(9)
C(3Q)-C(4Q)-C(10Q)	119.96(11)
C(3Q)-C(4Q)-H(4Q)	121.5(10)
C(10Q)-C(4Q)-H(4Q)	118.6(10)
O(5)-C(5)-C(4)	109.49(9)
O(5)-C(5)-H(5A)	108.5(9)
C(4)-C(5)-H(5A)	108.4(8)
O(5)-C(5)-H(5B)	107.8(9)
C(4)-C(5)-H(5B)	111.4(8)
H(5A)-C(5)-H(5B)	111.2(11)
C(4G)-C(5G)-N(7G)	110.80(9)
C(4G)-C(5G)-C(6G)	118.52(9)
N(7G)-C(5G)-C(6G)	130.67(9)
C(6Q)-C(5Q)-C(10Q)	120.62(11)
C(6Q)-C(5Q)-H(5Q)	120.6(11)
C(10Q)-C(5Q)-H(5Q)	118.7(11)
O(6G)-C(6G)-N(1G)	120.17(9)
O(6G)-C(6G)-C(5G)	128.46(10)
N(1G)-C(6G)-C(5G)	111.37(9)
C(5Q)-C(6Q)-C(7Q)	120.11(12)
C(5Q)-C(6Q)-H(6Q)	123.5(9)
C(7Q)-C(6Q)-H(6Q)	116.4(9)
C(8Q)-C(7Q)-C(6Q)	121.19(11)
C(8Q)-C(7Q)-H(7Q)	119.1(9)
C(6Q)-C(7Q)-H(7Q)	119.6(9)
N(7G)-C(8G)-N(9G)	112.75(9)
N(7G)-C(8G)-C(8Q)	122.97(9)
N(9G)-C(8G)-C(8Q)	124.17(9)
C(7Q)-C(8Q)-C(9Q)	119.43(10)
C(7Q)-C(8Q)-C(8G)	118.35(10)
C(9Q)-C(8Q)-C(8G)	122.19(10)
N(1Q)-C(9Q)-C(10Q)	121.93(10)
N(1Q)-C(9Q)-C(8Q)	118.88(10)
C(10Q)-C(9Q)-C(8Q)	119.18(10)
C(5Q)-C(10Q)-C(4Q)	123.00(11)
C(5Q)-C(10Q)-C(9Q)	119.42(11)
C(4Q)-C(10Q)-C(9Q)	117.58(11)

C(2G)-N(1G)-C(6G)	125.54(9)
C(2G)-N(1G)-H(1G)	115.8(9)
C(6G)-N(1G)-H(1G)	118.6(9)
C(2Q)-N(1Q)-C(9Q)	118.02(10)
C(2G)-N(2G)-H(2GA)	118.8(10)
C(2G)-N(2G)-H(2GB)	118.8(10)
H(2GA)-N(2G)-H(2GB)	121.6(14)
C(2G)-N(3G)-C(4G)	112.40(9)
C(8G)-N(7G)-C(5G)	104.67(8)
C(4G)-N(9G)-C(8G)	106.13(8)
C(4G)-N(9G)-C(1)	126.68(8)
C(8G)-N(9G)-C(1)	127.05(8)
C(1)-O(1)-C(4)	110.71(8)
C(3)-O(3)-H(3A)	102.7(12)
C(5)-O(5)-H(5)	108.2(12)
H(1WA)-O(1W)-H(1WB)	109.2(16)

Table S7. Anisotropic displacement parameters (A^2 x 10^3) for QG•H_2O. The anisotropic displacement factor exponent takes the form: -2 pi^2 [h^2 a*^2 U11 + ... + 2 h k a* b* U12]

	U11	U22	U33	U23	U13	U12
C(1)	20(1)	11(1)	14(1)	2(1)	2(1)	1(1)
C(2)	20(1)	14(1)	26(1)	2(1)	0(1)	-2(1)
C(2G)	15(1)	14(1)	16(1)	0(1)	-1(1)	-1(1)
C(2Q)	24(1)	30(1)	24(1)	3(1)	3(1)	-3(1)
C(3)	25(1)	13(1)	17(1)	-1(1)	5(1)	-4(1)
C(3Q)	33(1)	33(1)	23(1)	4(1)	10(1)	0(1)
C(4)	22(1)	10(1)	15(1)	-1(1)	0(1)	1(1)
C(4G)	15(1)	13(1)	13(1)	1(1)	0(1)	1(1)
C(4Q)	38(1)	29(1)	16(1)	1(1)	6(1)	5(1)
C(5)	17(1)	16(1)	16(1)	-1(1)	0(1)	1(1)
C(5G)	19(1)	12(1)	14(1)	0(1)	0(1)	0(1)
C(5Q)	37(1)	27(1)	15(1)	-3(1)	-5(1)	1(1)
C(6G)	15(1)	14(1)	16(1)	1(1)	-1(1)	0(1)
C(6Q)	34(1)	27(1)	22(1)	-4(1)	-7(1)	-4(1)
C(7Q)	26(1)	21(1)	20(1)	-1(1)	-1(1)	-2(1)
C(8G)	17(1)	12(1)	15(1)	-1(1)	1(1)	0(1)
C(8Q)	22(1)	13(1)	15(1)	-1(1)	0(1)	3(1)
C(9Q)	23(1)	14(1)	15(1)	-1(1)	0(1)	3(1)
C(10Q)	31(1)	19(1)	16(1)	-1(1)	0(1)	5(1)
N(1G)	21(1)	15(1)	12(1)	2(1)	0(1)	0(1)
N(1Q)	21(1)	22(1)	18(1)	1(1)	2(1)	0(1)
N(2G)	29(1)	17(1)	15(1)	-2(1)	1(1)	-2(1)
N(3G)	21(1)	12(1)	14(1)	-1(1)	0(1)	0(1)
N(7G)	22(1)	13(1)	15(1)	0(1)	0(1)	-1(1)
N(9G)	19(1)	10(1)	13(1)	1(1)	0(1)	0(1)
O(1)	19(1)	11(1)	18(1)	-4(1)	-2(1)	2(1)
O(3)	43(1)	13(1)	27(1)	5(1)	17(1)	1(1)
O(5)	18(1)	20(1)	22(1)	3(1)	1(1)	-1(1)
O(6G)	29(1)	12(1)	20(1)	3(1)	-1(1)	0(1)
O(1W)	27(1)	30(1)	20(1)	7(1)	4(1)	5(1)

Table S8. Hydrogen coordinates (x 10^4) and isotropic displacement parameters (A^2 x 10^3) for QG•$H_2$0.

	x	y	z	U(eq)
H(1)	8620(20)	-270(15)	1570(5)	18
H(2A)	5960(20)	-1431(16)	1257(5)	24
H(2B)	6670(20)	-968(17)	756(5)	24
H(2Q)	3050(20)	279(18)	2040(6)	31
H(3)	7750(20)	-3304(17)	703(5)	22
H(3Q)	3260(30)	356(19)	2830(6)	35
H(4)	11180(20)	-3039(16)	1162(5)	19
H(4Q)	6070(30)	1550(18)	3169(6)	33
H(5A)	10910(20)	-3409(16)	353(5)	20
H(5B)	10660(20)	-1644(16)	299(5)	20
H(5Q)	9320(30)	2819(17)	3054(6)	32
H(6Q)	11840(30)	3838(18)	2568(5)	33
H(7Q)	11370(20)	3569(17)	1789(5)	27
H(1G)	8710(20)	3037(15)	-464(5)	19
H(2GA)	8950(20)	-497(18)	-494(5)	24
H(2GB)	9000(20)	911(17)	-772(5)	24
H(3A)	8100(30)	-4540(20)	1282(6)	41
H(5)	13660(30)	-1545(19)	340(6)	30
H(1WA)	9270(30)	3620(20)	-1294(6)	38
H(1WB)	7710(30)	4420(20)	-1069(6)	38

Table S9. Weighted least-squares planes through the starred atoms (Nardelli, Musatti, Domiano & Andreetti Ric.Sci.(1965),15(II-A),807).
Equation of the plane: m1*X+m2*Y+m3*Z=d

Plane 1
m1 = -0.48823(0.00021)
m2 = 0.87243(0.00011)
m3 = -0.02252(0.00036)
D = -0.94404(0.00287)

Atom		d	s	d/s	(d/s)**2
N1Q	*	0.0106	0.0010	10.426	108.709
C2Q	*	0.0085	0.0013	6.583	43.340
C3Q	*	0.0010	0.0014	0.735	0.541
C4Q	*	0.0021	0.0013	1.588	2.523
C5Q	*	-0.0095	0.0013	-7.176	51.494
C6Q	*	0.0196	0.0014	14.201	201.668
C7Q	*	0.0176	0.0012	14.389	207.038
C8Q	*	-0.0180	0.0010	-17.121	293.140
C9Q	*	-0.0127	0.0011	-11.336	128.501
C10Q	*	-0.0097	0.0012	-8.406	70.665
					============
		Sum((d/s)**2) for starred atoms			1107.618

Chi-squared at 95% for 7 degrees of freedom: 14.10
The group of atoms deviates significantly from planarity

```
Plane  2
m1 =  -0.99982(0.00000)
m2 =  -0.00898(0.00032)
m3 =  -0.01661(0.00022)
D  =  -5.81638(0.00075)
Atom              d           s           d/s       (d/s)**2
N1G   *        -0.0088     0.0009      -9.365       87.710
C2G   *        -0.0243     0.0010     -24.228      586.985
N3G   *         0.0070     0.0009       7.506       56.346
C4G   *         0.0152     0.0011      14.206      201.810
C5G   *         0.0111     0.0011      10.349      107.092
C6G   *         0.0257     0.0011      24.034      577.617
N7G   *        -0.0132     0.0010     -13.156      173.072
C8G   *        -0.0204     0.0010     -20.373      415.078
N9G   *         0.0124     0.0009      13.250      175.568
O6G             0.0625     0.0009      71.942     5175.589
N2G            -0.0724     0.0011     -67.739     4588.575
C1             -0.0445     0.0011     -41.582     1729.076
C8Q            -0.1371     0.0011    -120.702    14568.961
C5Q            -0.4722     0.0014    -336.380   113151.609
O1W             0.2057     0.0009     219.868    48341.855
                                                ============
              Sum((d/s)**2) for starred atoms     2381.277
Chi-squared at 95% for  6 degrees of freedom: 12.60
The group of atoms deviates significantly from planarity

Dihedral angles formed by LSQ-planes
Plane - plane            angle (s.u.)             angle (s.u.)
  1      2               61.27( 0.02)             118.73( 0.02)
```

Table S10. Possible hydrogen bonds

Donor-H	Donor...Acceptor	H...Acceptor	Donor-H......Acceptor
C1 -H1	C1 ...N1Q (0)	H1 ...N1Q (0)	C1 -H1 ...N1Q (0)
0.957(.015)	3.193(.001)	2.652(.014)	116.30(1.05)
1.080		2.600	113.87 (**)
C2 -H2B	C2 ...N3G (0)	H2B ...N3G (0)	C2 -H2B ...N3G (0)
0.982(.016)	3.108(.001)	2.432(.016)	125.59(1.16)
1.080		2.377	123.67 (**)
C5 -H5B	C5 ...N3G (0)	H5B ...N3G (0)	C5 -H5B ...N3G (0)
1.000(.015)	3.330(.001)	2.441(.015)	147.83(1.18)
1.080		2.374	146.81 (**)
C7Q -H7Q	C7Q ...N7G (0)	H7Q ...N7G (0)	C7Q -H7Q ...N7G (0)
0.955(.016)	3.054(.002)	2.893(.016)	90.47(0.99)
1.080		2.895	87.98 (**)
**N1G -H1G**	**N1G ...O1W (0)**	**H1G ...O1W (0)**	**N1G -H1G ...O1W (0)**
**0.912(.015)**	**2.783(.001)**	**1.876(.015)**	**172.86(1.36)**
**1.030**		**1.759**	**172.38 (**)**
N2G -H2GB	N2G ...O1W (0)	H2GB ...O1W (0)	N2G -H2GB ...O1W (0)
0.886(.016)	3.373(.001)	2.682(.016)	135.79(1.29)
1.030		2.580	133.55 (**)

```
C2    -H2A      C2    ...O5    ( 1)  H2A   ...O5    ( 1)  C2    -H2A   ...O5    ( 1)
0.984(.016)     3.107(.001)          2.942(.016)           90.31( 0.98)
1.080                                2.943                 88.44       (**)

C2    -H2B      C2    ...O5    ( 1)  H2B   ...O5    ( 1)  C2    -H2B   ...O5    ( 1)
0.982(.016)     3.107(.001)          2.640(.015)          109.37( 1.05)
1.080                                2.609                107.34       (**)

C3    -H3       C3    ...O6G   ( 2)  H3    ...O6G   ( 2)  C3    -H3    ...O6G   ( 2)
1.021(.015)     3.373(.001)          2.452(.015)          149.52( 1.14)
1.080                                2.402                148.81       (**)

C5    -H5A      C5    ...O6G   ( 2)  H5A   ...O6G   ( 2)  C5    -H5A   ...O6G   ( 2)
1.006(.015)     3.200(.001)          2.301(.015)          148.31( 1.17)
1.080                                2.238                147.31       (**)

O3    -H3A      O3    ...N7G   ( 2)  H3A   ...N7G   ( 2)  O3    -H3A   ...N7G   ( 2)
0.888(.020)     2.841(.001)          1.956(.020)          174.17( 1.84)
0.938                                1.906                174.02       (**)

C3Q   -H3Q      C3Q   ...O3    ( 3)  H3Q   ...O3    ( 3)  C3Q   -H3Q   ...O3    ( 3)
0.989(.018)     3.247(.002)          2.608(.018)          122.41( 1.27)
1.080                                2.560                120.69       (**)

C4Q   -H4Q      C4Q   ...O3    ( 3)  H4Q   ...O3    ( 3)  C4Q   -H4Q   ...O3    ( 3)
0.986(.016)     3.392(.002)          2.895(.017)          112.07( 1.13)
1.080                                2.862                110.34       (**)

C5    -H5A      C5    ...N2G   ( 4)  H5A   ...N2G   ( 4)  C5    -H5A   ...N2G   ( 4)
1.006(.015)     3.300(.001)          2.768(.015)          113.44( 1.01)
1.080                                2.740                112.01       (**)

C5    -H5B      C5    ...O6G   ( 5)  H5B   ...O6G   ( 5)  C5    -H5B   ...O6G   ( 5)
1.000(.015)     3.261(.001)          2.732(.015)          113.44( 1.02)
1.080                                2.701                111.88       (**)

O5    -H5       O5    ...O6G   ( 5)  H5    ...O6G   ( 5)  O5    -H5    ...O6G   ( 5)
0.859(.017)     2.779(.001)          1.947(.017)          162.43( 1.65)
0.938                                1.872                161.71       (**)

O1W   -H1WA     O1W   ...N1Q   ( 5)  H1WA  ...N1Q   ( 5)  O1W   -H1WA  ...N1Q   ( 5)
0.879(.019)     2.865(.001)          2.004(.019)          166.12( 1.76)
0.938                                1.947                165.70       (**)

O1W   -H1WA     O1W   ...N9G   ( 5)  H1WA  ...N9G   ( 5)  O1W   -H1WA  ...N9G   ( 5)
0.879(.019)     3.461(.001)          2.976(.020)          116.70( 1.53)
0.938                                2.950                115.67       (**)

C5Q   -H5Q      C5Q   ...O3    ( 6)  H5Q   ...O3    ( 6)  C5Q   -H5Q   ...O3    ( 6)
0.943(.016)     3.580(.002)          2.930(.017)          127.18( 1.22)
1.080                                2.850                125.00       (**)

C5Q   -H5Q      C5Q   ...O1    ( 6)  H5Q   ...O1    ( 6)  C5Q   -H5Q   ...O1    ( 6)
0.943(.016)     3.495(.001)          2.603(.016)          158.02( 1.33)
1.080                                2.477                156.84       (**)

N2G   -H2GA     N2G   ...O5    ( 7)  H2GA  ...O5    ( 7)  N2G   -H2GA  ...O5    ( 7)
0.885(.016)     2.972(.001)          2.112(.016)          163.66( 1.49)
1.030                                1.973                162.48       (**)

O1W   -H1WB     O1W   ...N9G   ( 8)  H1WB  ...N9G   ( 8)  O1W   -H1WB  ...N9G   ( 8)
0.897(.019)     3.244(.001)          2.794(.020)          112.39( 1.45)
0.938                                2.779                111.61       (**)

O1W   -H1WB     O1W   ...O1    ( 8)  H1WB  ...O1    ( 8)  O1W   -H1WB  ...O1    ( 8)
0.897(.019)     2.904(.001)          2.025(.019)          166.01( 1.75)
0.938                                1.985                165.72       (**)

Number of possible hydrogen bonds  23
```

(**) Values normalized following G.A.Jeffrey & L.Lewis, Carbohydr.Res. (1978).60,179; R.Taylor, O.Kennard, Acta Cryst.(1983).B39,133.

Equivalent positions:

```
( 0)    x,y,z
( 1)    x-1,+y,+z
( 2)    x,+y-1,+z
( 3)   -x+1,+y+1/2,-z+1/2
( 4)    x+1/2,-y-1/2,-z
( 5)    x+1/2,-y+1/2,-z
( 6)   -x+2,+y+1/2,-z+1/2
( 7)    x-1/2,-y-1/2,-z
( 8)    x-1/2,-y+1/2,-z
```
